# Sickle Cell Trait Induces Oxidative Damage on *Plasmodium falciparum* Proteome at Erythrocyte Stages

**DOI:** 10.3390/ijms20225769

**Published:** 2019-11-16

**Authors:** Alber Díaz-Castillo, Neyder Contreras-Puentes, Ciro Alvear-Sedán, Carlos Moneriz-Pretell, Erika Rodríguez-Cavallo, Darío Mendez-Cuadro

**Affiliations:** 1 Analytical Chemistry and Biomedicine Group, Exacts and Natural Sciences Faculty, University of Cartagena, 130015 Cartagena, Colombia; alberdiazcastillo96@gmail.com; 2 Analytical Chemistry and Biomedicine Group, Pharmaceuticals Sciences Faculty, University of Cartagena, 130015 Cartagena, Colombia; neidercontreras11@gmail.com; 3 Biochemistry and disease Group, Medicine School, University of Cartagena, 130015 Cartagena, Colombia; calvears@unicartagena.edu.co (C.A.-S.); cmonerizp@unicartagena.edu.co (C.M.-P.)

**Keywords:** *Plasmodium falciparum*, asexual stages, sickle cell trait, protein carbonylation, carbonyl index

## Abstract

The presence of hemoglobin A-S (HbAS) in erythrocytes has been related to the high production of reactive oxygen species (ROS) and an increased in intracellular oxidative stress that affects the progress of *Plasmodium* erythrocytic cycle life and attenuates its serious clinical symptoms. Nevertheless, oxidative effects on *P. falciparum* proteome across the intraerythrocytic cycle in the presence of HbAS traits have not been described yet. Here, an immune dot-blot assay was used to quantify the carbonyl index (C.I) on *P. falciparum* 3D7 proteome at the different asexual erythrocytic stages. Protein carbonylation on parasites cultivated in erythrocytes from two donors with HbAS increased 5.34 ± 1.42 folds at the ring stage compared to control grown in hemoglobin A-A (HbAA) red blood cells. Whereas at trophozoites and schizonts stages were augmented 2.80 ± 0.52 and 3.05 ± 0.75 folds, respectively. Besides proteins involved in processes of the stress response, recognition and invasion were identified from schizonts carbonylated bands by combining SDS-PAGE with MALDI-TOF-TOF analysis. Our results reinforce the hypothesis that such oxidative modifications do not appear to happen randomly, and the sickle cell trait affects mainly a small fraction of parasite proteins particularly sensitive to ROS.

## 1. Introduction

Malaria is one of the oldest diseases in the world, during the last 70,000 years of co-evolution with humans it has generated a selective pressure on the human genome and produces the appearance of erythrocyte polymorphisms, such as structural and quantitative hemoglobinopathies [1]. These constitute an important health problem, mainly in malaria-endemic areas with a high frequency of individual carriers, not only in their countries of tropical and subtropical origin but globally as a result of the augment of massive human migrations [2].

It has been estimated that 5% of the world population has genes related to hemoglobinopathies, being sickle cell anemia the most common hemoglobinopathy in the United States, Latin America and the Caribbean [3,4] and the individuals affected can be homozygous (HbSS) or heterozygotes (HbAS). The highest prevalence of the sickle cell trait occurs in equatorial and western Africa reaching around 40%. In Latin America, countries as Brazil and Colombia reach a global prevalence between 5–6% and 6–10%, respectively. Nevertheless, in some geographical regions with a great number of Afro-American populations prevalence exceeds 10% [3].

Although the beneficial effects of HbAS against clinical symptoms of severe malaria are well accepted, the mechanisms of protection are not completely clarified [5]. Among the biochemical and immune-mediated mechanisms proposed are reduced adherence of parasitized erythrocytes [6], the improvement of the host immune response [7], the arresting of parasite growth [8] and the overexpression of the enzyme heme-oxygenase 1 [9].

Many of these metabolic pathways somehow affect the fine redox balance between parasite and the host [10,11] and could act on specific protein targets involved in several various metabolic processes, such as the remodeling of the host cell membrane and export of proteins parasite Cytoskeletal membrane proteins of *P. falciparum* infected erythrocytes seem particularly susceptible to oxidative damage during infection under glucose-6-phosphate dehydrogenase (G6PD) deficiency and HbAS trait conditions [11,12].

Nevertheless, the oxidative effects caused on the proteome of *P. falciparum* through the intraerythrocytic cycle in the presence of HbAS trait have not been described yet. Protein carbonylation is considered a major hallmark of oxidative stress-related disorders, and it is one of the most harmful irreversible oxidative protein modifications. Its measurements are often performed to assess the extent of oxidative stress under different contexts of cellular damage [13]. Therefore, the degree of oxidation of the *P. falciparum* 3D7 proteome was quantified, and potentially carbonylated proteins were identified as a consequence of the presence of the sickle cell trait.

## 2. Results

### 2.1. Cultures of P. falciparum 3D7 on HbAS Carriers and Control Donors

**Figure 1:**
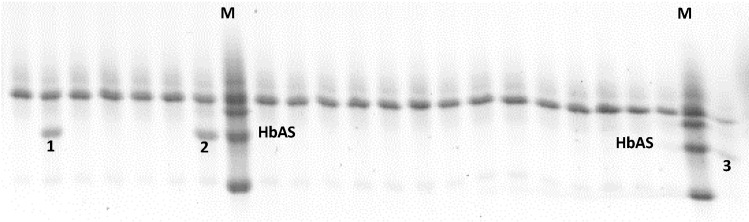
Horizontal hemoglobin electropherogram for the identification of HbAS carriers. Numbered bands show the presence of hemoglobin S (HbAS) for JT (1), HH (2) and MJ (3) donors, respectively. M: Hb marker.

A random sample of 50 healthy donors was assayed to identify carriers of the sickle cell trait. Horizontal hemoglobin electrophoresis was positive for three donors, reaching 6% prevalence and the HbAS percentages obtained by the densitometric analysis were 36.4% (JT-donor), 41.7% (HH-donor) and 43.0% (MJ-donor; Figure 1).

**Figure 2:**
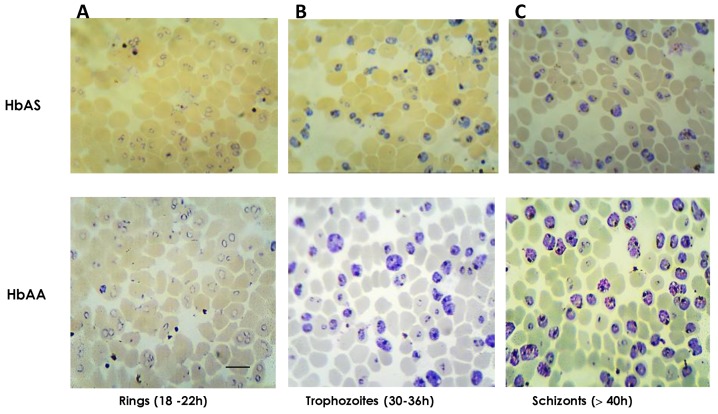
Synchronous cultures of *P. falciparum* 3D7 asexual stages. Parasites were grown in HbAA and HbAS red blood cells (RBCs). Asexual typical forms were harvested for different stages. (**A**). Late rings (14–20 h), (**B**). mature trophozoites (30–36 h) and (**C**). schizonts (>40 h). Smears were stained with Giemsa 10% and magnified 100×. Scale bar = 10 μm.

Synchronous cultures of *P. falciparum* were harvested at rings, trophozoites and schizonts stages for each donor, three HbAS and a control HbAA. Around 800 μL were collected with parasitemias in a range of 34–40% as showed in Figure 2. Details about harvested cultures are summarized in Supplementary Table S1. Any morphological differences between HbAA and HbAS cultures were not observed during the progression of the parasite erythrocytic cycle.

Next, cultures were lysed with a sorbitol solution to obtain the fraction of infected red blood cells (iRBCs) membrane proteins, which were used in other studies [11]. Then, pellets were treated with sample buffer (Tris-HCl 50 mM pH 8, NaCl 50 mM and SDS 1%) to obtain parasite proteins in a range of 0.6–2.3 μg/μL, which were used to subsequently assays (see Supplementary Table S2).

### 2.2. Quantitation of Carbonyl Index by Dot-Blot

**Figure 3:**
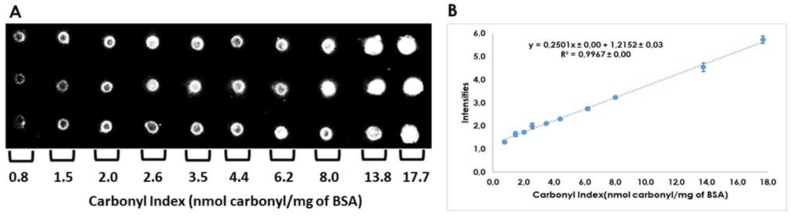
Calibration curve of carbonylated proteins for quantitation of carbonyl indexes by dot-blot. (**A**). 2 μL of DNPH derivatized bovine serum albumin (BSA; 100 ng/μL) from standard working solutions were spotted by triplicate on PVDF membranes. (**B**). Mean from three calibration curves analyzed in two different days. The calibration curve was derived from reproducibility assay.

Linearity, repeatability, and reproducibility were assayed for the carbonylated proteins calibration curve. Calibration curve of bovine serum albumin (BSA) shown linearity for a range of carbonyl index values between 0.8 to 17.7 nmol carbonyl/mg proteins (R^2^ > 0.997, see Figure 3). Repeatability was assayed with curves built on the same day, whereas reproducibility was calculated with data of two different days. RSDs obtained were lower than 1.3% for slope values of the curves constructed, establishing that no statistical differences were found (*p* < 0.05). Raw data used to evaluate repeatability, and reproducibility is provided in Supplementary Table S3, and Supplementary Figure S1.

Next, carbonyl indexes were determined for *P. falciparum* proteins from parasites grown in HbAA and HbAS red blood cells (RBCs). Oxy dot-blots obtained are showed in Supplementary Figure S2 and carbonyl index data are summarized in Table 1.

For parasites grown in control HbAA erythrocytes, protein carbonylation increased with the progress of parasite cycle life, being greater at trophozoites and schizonts than in the ring stages; whereas parasites grown in HbAS cells showed a significant increase of carbonyl indexes from early ring stage.

To measure the variation of oxidative damage suffered by proteome parasites, the carbonyl index quotient HbAS/HbAA was calculated. Thus, for example, carbonyl index for normal HbAA iRBCs at ring stage was 12.7 ± 1.07 nmol of carbonyls/mg of parasite proteins; whereas HbAS donor with code ST reached 88.0 ± 2.10 nmol of carbonyls/mg of protein. The quotient 88.0/12.7 indicates an increment of 6.92 folds on the irreversible oxidative damage suffered by parasite proteins when it is cultured in HbAS erythrocytes. Individual quotients for all samples are summarized in Table 2, and the behavior of these data appears plotted in Supplementary Figure S3.

### 2.3. Determination of P. falciparum Protein Carbonylation Patterns on HbAA and HbAS Carriers

**Figure 4:**
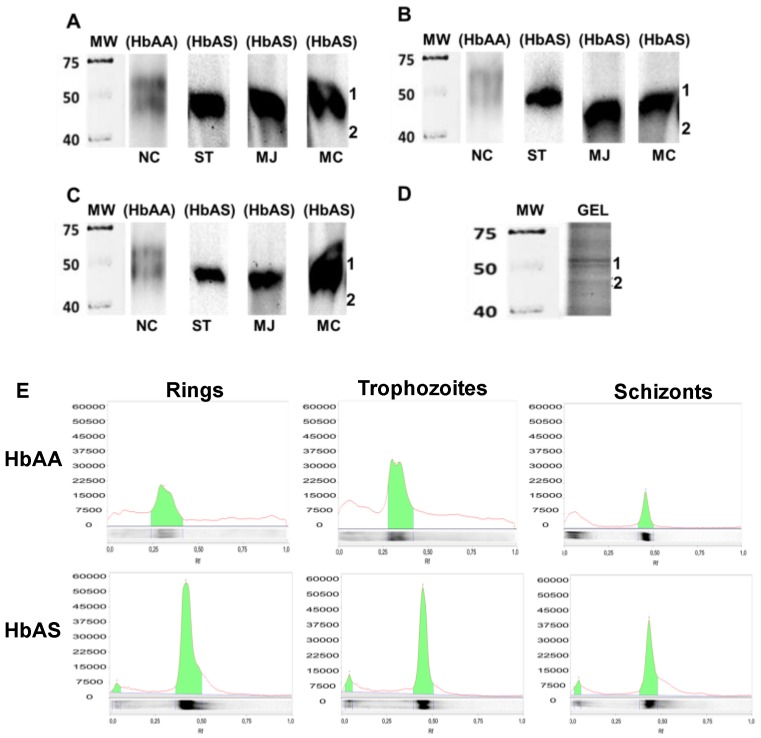
Profile of *P. falciparum* protein carbonylated bands at different asexual stages. Panels show protein oxidized profiles obtained at rings (**A**), trophozoites (**B**) and schizonts (**C**) from HbAS control and HbAS carriers. NC: HbAA control. ST, MC, and MJ: HbAS donors. Panel **D** shows the preparative polyacrylamide gel stained with Coomassie Blue Brilliant (CBB) for schizont’s proteins that match with the oxyblots. Of parasite proteins 2.5 μg derivatized with DNPH were used in oxyblots, while 50 μg were electrophoresed in preparative SDS-PAGE. Panel **E** displays densitograms of carbonylated protein bands detected in oxyblots across the erythrocytic cycle for HbAA control and ST HbAS donor. Densitograms were analyzed with ImageLab software^®^ to determine variations in the intensity of the signal for the detected carbonylated bands (panel E, Figure 4). For parasite proteins from erythrocytes, HbAA recorded the trophozoites stage as the most oxidizing, while the ring stage was the more carbonylated phase in HbAS proteins. This behavior reinforces the result obtained previously by dot-blot.

Qualitative profiles of carbonylated proteins of *P. falciparum* were obtained at the rings, trophozoites, and schizonts stages in samples of control (HbAA) and HbAS carriers. Results obtained show a wide and intense protein carbonylated band around 50 kDa in all donors with HbAS that appears as a weak double band for HbAA control (Figure 4). This pattern was kept constant across the different parasite asexual stages.

**Figure 5:**
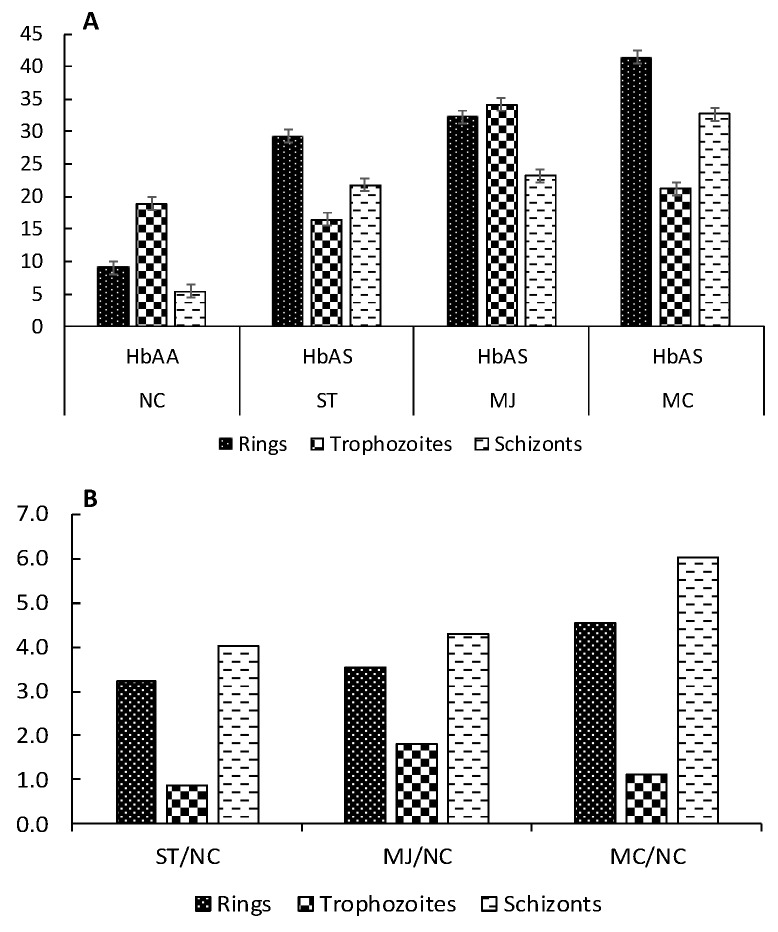
Intensities of *P. falciparum* proteins carbonylated bands across asexual stages. In panel **A** is shown individual intensity values for carbonylated bands by donors and erythrocytic stage; while in panel **B** is plotted the quotients HbAS/HbAA.

In a similar way to carbonyl indexes, the quotients HbAS/HbAA were calculated for carbonylated bands and plotted in Figure 5. Data of adjusted intensities are listed in Supplementary Tables S5 and S6. Results obtained, again support the presence of increased oxidative damage from the ring stage, associate with the presence of the sickle cell trait.

### 2.4. Identification of Proteins in Carbonylated Bands

Finally, three protein bands in Coomassie Blue Brilliant (CBB) stained gel matched with the carbonylated bands observed in oxyblots (Figure 4). The first and second bands appeared as double and were numbered 1.1 and 1.2; while the last one was numbered 2. Bands were excised from rings and schizonts lanes gel and processed as was above mentioned. Mixtures of parasite and human proteins were identified by MS/MS analysis only in schizonts bands, and they are listed in Table 3. No parasite protein bands were identified from rings, and the human ones detected were coincident with those identified in schizonts.

## 3. Discussion

Erythrocyte redox balance is found permanently disturbed in erythrocytes harboring hemoglobin S. In the case of sickle cell disease (HbSS), clinical cumulative effects are intimately linked to a chronic and systemic oxidative stress [14]. While in its heterozygous form (HbAS), the altered status redox is part of the uncomfortable environmental conditions for the free development of *Plasmodium* gender parasites and involves the activation of multiple cellular mechanisms of resistance to clinical severe symptoms of the infection [15].

In this sense, Archer has proposed a protector mechanism, based on HbAS polymerization in infected RBCs with low O_2_ concentrations, due to its capability to arrest in vitro parasite growing [15]. Nevertheless, HbAS polymerization is a reversible process by agents as CO and CO_2_ and hence the growth of the parasite could be restored in the presence of these gases [15,16]. In our culture conditions, parasites were maintained at 5% CO_2_ and the hematocrit kept low by medium changes according to the method of Radfar [17], which may explain why we do not observe alterations in the progression of the parasite cycle life in HbAS erythrocytes.

Oxidative stress, on the other hand, is one of the body’s important defensive immune mechanisms during malaria infection, affecting its progress and clinical outcome. Thus, for example, the high and rapid production of ROS during the acute phase of infection may be beneficial to reduce parasitemia [18]. Oxidative stress induced by antimalarial agents, as chloroquine and artemisinin, is also important in malaria parasite clearance. Nevertheless, chronic stimulation of ROS or its excessive production, are deleterious to the host cells and favors massive hemolysis with eventual metabolic acidosis and respiratory distress [18,19].

Despite this background, the oxidative damage suffered by *P. falciparum* parasites as a consequence of metabolic conditions resident in erythrocytes with HbAS has not been quantified and characterized yet.

Thus, we calculated the degree of protein oxidation on the *P. falciparum* proteome in vitro during their asexual phase in erythrocytes carrying the sickle trait employing a quantitative dot-blot assay. For this, donors with 36.4–43.0% of HbAS were identified by horizontal hemoglobin electrophoresis. A 6% prevalence was calculated in our random sample, establishing in the range of 5–10% reported for some regions of the Colombian territory [20,21]. Next, parasites were cultured and processed to obtain proteomes at ring, trophozoites and schizonts stages. Repeatability and reproducibility of the protein carbonylated calibration curve proved to be suitable for the quantification of carbonyl indexes and the evaluation of the oxidative damage suffered by the *P. falciparum* proteome throughout its asexual life cycle. The behavior of the calibration curve was like those reported previously [11,22].

Results obtained for control HbAA showed low carbonyl indexes during the ring stage, a maximum of oxidative response in trophozoites, followed by a little decrease during the schizont stage. This behavior is congruent with the development of the parasite, which shows maximum metabolic activity during the trophozoite stage. In this period the parasite consumes hemoglobin and releases pro-oxidant products derived from free heme [23,24]; however, *P. falciparum* prevents its deleterious effects by increasing of their efficient antioxidant systems, such as the GluPho enzyme that corresponds to the combination of the G6PD of the parasite with the second enzyme of the pathway of the pentoses, 6-phosphogluconolactonase (PfG6PD-6PGL) [25,26].

Under conditions of HbAS trait, the behavior of the carbonyl indexes of the proteome of *P. falciparum* evidenced substantial changes. In all stages, the oxidative damage increased compared to control, being largest at the ring stage where it was 5.34 times larger than in control, while it was only almost twice as large in trophozoites and schizonts. We have previously measured the particular increase in oxidative stress occurring in the ring stage and how it mainly affects the host cell membrane proteins in protective red cell polymorphisms of severe malaria, such as G6PD deficiency, blood group O and HbAS [11,12,27]. Susceptibility of host proteins to oxidative stress during the ring stage would be related to the low expression described for the main antioxidant enzymes and heat shock proteins of the parasite [28]. These sets of evidence indicate that the biochemical mechanisms operate through irreversible oxidative damage, and confer advantages to HbAS carrier concerning severe malaria symptoms, occurring or beginning at the early ring stage and affecting the host cell proteins.

Although at trophozoites and schizonts stages, carbonyl indexes decreased in parasites grown in HbAS erythrocytes compared to rings stage, the oxidative damage on parasite proteome is still larger than those observed in parasites cultured in control HbAA RBCs. This behavior is consistent with some antioxidant mechanisms associated with the pentose phosphate pathway that increases its activity during trophozoites, decreases in the schizont stage but maintains an activity increased 10 times greater than during the early stages of the parasite [29]. This route is fundamental due to its function in the synthesis of nucleotides and the reduction of nicotinamide adenine dinucleotide phosphate (NADP) to nicotinamide adenine dinucleotide phosphate reduced (NADPH).

Therefore, quantitation of the carbonyl index of the parasite proteome seems to be a good marker to measure the oxidative changes on *P. falciparum* proteome across the intraerythrocytic cycle under different genetic backgrounds or cultured conditions.

On the other hand, analysis of protein carbonylated profiles obtained by Western blot in our conditions showed oxidative damage focused, constant and limited to a reduced size zone for the parasite proteome during all asexual stages. This finding is similar to the one found by Radfar, who described a series of carbonylated proteins that remained nearly constant throughout the life cycle of *P. falciparum* clone Dd2 in control samples not exposed to chloroquine [19] and reinforce that such modifications do not appear to happen randomly [30]. Herein, our results indicate that the pro-oxidant environment residing in HbAS erythrocytes only increases the intensity of the oxidative damage for those small fractions of parasite proteins particularly sensitive to ROS.

Five schizonts parasite proteins were identified into this group along with the erythroid band-3 and hemoglobin, which indicates a good performance for the parasite protein obtention method applied due to a reduced presence of host cell proteins. Two identified parasite proteins, heat shock 70 and disulfide isomerase, are chaperones involved in the metabolic pathways of the stress response; while beta-galactosidase is a glycolytic enzyme involved in the process of catabolic galactose. These parasite proteins make part of the macromolecule networks involved in the adaptation of *P. falciparum* to hyperoxic stress and were classified by Radfard as highly susceptible to oxidative damage [19,30]. Besides, it should be taken into account that 2% of the parasite genome encodes chaperone proteins playing diverse important roles in the development of the parasite [31].

In the case of rhoptry-associated protein 2 (RAP-2), it is ubicated within the rhoptries of *Plasmodium falciparum* forming a low molecular weight complex along to RAP-1 and RAP-3. These merozoite proteins play an important role in erythrocyte invasion [32]. They have functions of protease, integral membrane protein, erythrocyte-binding protein and domain oxidoreductase and participate in the initial recognition and generation of the parasitophorous vacuole and the modification of the host cell [33]. Other protein involved in erythrocyte invasion was identified in carbonylated bands, the *P. falciparum* surface protein, which is related to the heat shock 70 KDa [34]. Composition and function of merozoite surface proteins (MSPs) and rhoptries has been of great interest either role in RBC invasion and potential as vaccine candidates [33] but how they are affected by oxidative posttranslational modifications is something not revealed yet.

Finally, host cell protein band 3 is a glycoprotein in the erythrocyte membrane, which carries out the exchange of chloride/bicarbonate, a process necessary for cellular respiration [35]. The oxidation of band-3 causes its clusterization and disruption of their complexes with other proteins as cytoskeletal ankyrin [36]. Clusterization can be also promoted by binding of hemoglobin by-products (hemichromes) to the cytoplasmic domain of band-3. Thus, eliciting the humoral immune response to remove the affected red blood cells from the circulation [10,37]. In fact, Band 3 had been identified as carbonylated in *P. falciparum* infected erythrocytes with other protector polymorphism of severe malaria [11,27].

Overall, any irreversible posttranslational modification as carbonylation could affect the structure and functionality of proteins. In particular, it has been demonstrated that direct carbonylation of lateral chains of lysine and arginine residues is equivalent to replacing a hydrophilic residue with a hydrophobic one, which by itself significantly increases the intrinsic aggregation propensity of the protein [38]. More recently, we showed how the indirect carbonylation with 4-hydroxy-2-nonenal induces structural local changes and disturbs the conformational stability, folding and flexibility of oxidized proteins, increasing its lipophilic potential and altering their electrostatic potential [39]. Although we did not discriminate between direct and indirect carbonylation, our results reinforced the participation of protein carbonylation as mediators in mechanisms of resistance to severe malaria in the sickle cell trait.

## 4. Materials and Methods

### 4.1. Identification of Donor’s Hemoglobin S Carriers

Hemoglobin S (HbAS) from voluntary donors was identified by horizontal hemoglobin electrophoresis. All donors gave their informed consent for inclusion before they participated in the study, and the protocol was approved by the Ethics Committee of the University of Cartagena (Project Actas 013-2017/ 011-2017, 16-05-2017. First, the capillary blood samples were placed on filter paper (Whatman 1), deposited in a 96 well microplate, with the addition of an elution solution for 30 min. Then, the electrophoresis cabinet (Migele Gel Electrophoresis Unit, PerkinElmer, 940 Winter Street, Waltham, MA, USA) was prepared by placing a strip in the center, and two strips on the sides (right and left); the strips were previously embedded in anodic, and cathodic solutions, respectively. Subsequently, 1% agarose (Sigma, St Louis, MO, USA) gel was added, samples were loaded and electrophoresed at 120 V for 90 min. After electrophoresis, the gel was submerged in 30% TCA (Sigma) for 5 min, followed by washing with Mili-Q water and drying overnight. The identification of HbAS carriers was based on the Rf with the Hb marker provided in the commercial kit of neonatal hemoglobin RESOLVE^®^ (PerkinElmer, 940 Winter Street, Waltham, MA, USA). The percentage of Hb expression was measured by density analysis with the Quantity One software (BioRad, Hercules, CA, USA).

### 4.2. Plasmodium falciparum 3D7 Cultures and Parasite Protein Obtention

*P. falciparum* clone 3D7 was gifted by Dr. Sara Robledo from the PECET group of the University of Antioquia. Synchronous cultures of *P. falciparum* 3D7 with high parasitemia were grown by triplicates in RBCs from control and three HbAS donors, following strictly the protocol previously described by Radfar [17]. Synchronization was based on sorbitol 5% (Sigma) and Percoll 90% (GE Healthcare, Pittsburgh, PA, USA) methods; the progress of asexual stages was monitored by stained smears with Giemsa 10% (Merck, St Louis, MO, USA). Infected red blood cells (iRBCs) at rings, trophozoites and, schizonts stages were harvested from each donor and finally stored at −40 °C until processing. Samples were treated individually for all subsequent assays.

The parasite proteins were obtained by combining two protocols previously described. First, iRBCs at trophozoites and schizonts stages were lysed with 5% sorbitol solution for 10 min at 37 °C and subsequently centrifuged at 2300 rpm and 4 °C. This procedure was repeated several times until obtaining a colorless supernatant [27]. Second, ring iRBCs were incubated 5 min with saponin 0.05% (Sigma) at room temperature, centrifuged 14,000× *g* for 10 min at 4 °C and washed three times with ice-cold PBS, followed by one wash in 10 mM Tris-HCl, pH 7.4 (Sigma) [19]. Finally, parasite pellets were homogenized in sample buffer (Tris-HCl 50 mM pH 8, NaCl 50 mM and SDS 1%) using four cycles of freezing and thawing steps. Samples were centrifuged at 4 °C to obtain supernatants rich in parasite proteins, which were quantified and stored at −40 °C [40].

### 4.3. Carbonyl Index Quantitation of Parasite Protein Fractions

Spectrophotometry and immune dot-blot methods were applied to measure the carbonyl index. For this purpose, an aliquot of the BSA fatty acid-free solution (5 μg/μL, Sigma) was derivatized with a DNPH probe, and their carbonyl index was measured in alkaline medium at 450 nm [41]. Its value corresponds to the basal carbonyl index in our conditions (non-oxidized BSA). In parallel, an aliquot of the BSA solution was oxidized with 10 mM FeSO_4_ by incubation at 37 °C for 2 h. In both cases, basal and oxidized derived BSA was precipitated with 10% TCA for 15 min and the pellets obtained were washed with 85% acetone for 30 min and centrifuged at 4 °C, 7000 rpm for 5 min. Supernatants were discarded, and the pellets were dissolved in PBS. Subsequently, oxidized and non-oxidized (basal) BSA solutions were quantified with the Bradford assay for normalization at 1 µg/µL [40], and quantification of the carbonyl index values by the alkaline medium at 450 nm.

Next, stoichiometric mixtures of the standard proteins (oxidized and basal) were completed to obtain standards solutions with intermediates carbonyl index values. These standard solutions were used to build the protein carbonylated calibration curve of the dot-blot assay. For its purpose, all standards solutions were derivatized using 6% SDS for 2 min and 10 mM DNPH in 2 N HCl for 10 min, and the reaction was stopped by employing Tris/Glycerol supplemented with β-mercaptoethanol 85:15 [22,42] ( all reagents from Sigma).

Afterward, 200 ng of each standard were spotted by triplicate on the PVDF membrane (GE Healthcare, Pittsburgh, PA, USA), blocked with PBS-5% milk for 2 h, incubated with Anti-DNP primary antibody (Merck) (1:5000) for 2 h, followed by incubations with Anti-Rabbit secondary antibody (Thermo Fischer Scientific, Burlington, ONT, Canada) (1:5000) for 1 h.

Membranes were washed with PBS-Milk-Tween 20, PBS-milk and PBS for 5 min and revealed by chemiluminescence using Kit-Nova for 3 min [11]. The images were captured by a ChemiDoc System (Biorad, Hercules, CA, USA) and the intensity of the spots was analyzed using the Image Lab 6.0 software (Biorad). Replicates were built at different days to evaluate repeatability, reproducibility and linearity of carbonylated protein calibration curves.

To quantify carbonyl index values in samples from parasite proteins at different stages (rings, trophozoites and schizonts), they were DNPH-derivatized, diluted ten folds in PBS, spotted on PVDF membranes, and incubated with the antibodies replicating the methodology described above.

Data were analyzed with GraphPad Prism 6.0 (GraphPad, San Diego, CA, USA) and presented as the mean ± standard error. C.I.s data, repeatability and reproducibility were analyzed by Student’s *t*-test using the parametric test in one-tailed. Statistical significance between groups was established at *p* < 0.05.

### 4.4. Determination of Carbonylation Patterns on P. falciparum 3D7 Proteins

To study the oxidative damage suffered by *P. falciparum* 3D7 across the intraerythrocytic cycle in HbAS RBCs, parasite proteins DNPH-derivatized (2.5 μg) were electrophoresed on 10% SDS–PAGE at 90 and 120 V for 150 min (Miniprotean tetracell, Biorad, Hercules, CA, USA). Gels were transferred 30 min on PVDF membranes employing a semi-dry Trans-Blot turbo transfer apparatus (Biorad, Hercules, CA, USA). PVDF membranes were blocked for 2 h at room temperature with 5% nonfat dry milk in PBS. Then, membranes were incubated with rabbit polyclonal anti-DNPH antibodies (Merck, St Louis, MO, USA) at 1:5000 in PBS-milk 5%, for 2 h at room temperature with gentle rocking, followed by incubation with peroxidase-linked anti-rabbit IgG antibody (Thermo Fischer Scientific, Burlington, ONT, Canada) at 1:5000 for 1 h at room temperature. Chemiluminescence signals were developed as was mentioned for the dot-blot assay.

Preparative SDS-PAGE, loaded with 50 μg by lane, were run at the same conditions and stained with Coomassie Blue Brilliant (CBB) following a general protocol [12]. Next, they were matched against oxyblots to select protein carbonylated bands.

### 4.5. Identification of P. falciparum 3D7 Proteins by Mass Spectrometry

The carbonylated protein bands selected for MS analysis were reduced in gel, alkylated and digested with trypsin according to Sechi et al. [43]. Briefly, samples were reduced with 10 mM DTT in 25 mM NH_4_HCO_3_ for 30 min at 56 °C and subsequently alkylated with 55 mM iodoacetamide in 25 mM ammonium bicarbonate for 15 min in the dark. Finally, samples were digested with 12.5 ng/µL sequencing grade Bovine Trypsin (Roche Molecular Biochemicals, Penzberg, Germany) in 25 mM NH_4_HCO_3_ (pH 8.5) overnight at 37 °C.

After digestion, the supernatant was collected, and 1 µL was spotted onto a MALDI target plate and allowed to air-dry at room temperature. Then, 0.8 µL of a 3 mg/mL of an α-cyano-4-hydroxy-cinnamic acid matrix (Sigma) in 50% acetonitrile 0.1% TFA were added to the dried peptide and allowed again to air-dry at room temperature.

MALDI-TOF-TOF analysis, was performed at the Proteomics Unit at Complutense University of Madrid, employing a tandem mass spectrometer, 4800 Plus Proteomics Analyzer MALDI-TOF-TOF (Applied Biosystems, MDS Sciex, Toronto, Canada) equipped with a Nitrogen laser diode-pumped Nd: YAD 355 nm, delayed extraction and reflector, in positive-ion reflector mode (the ion acceleration voltage was 20 kV to MS acquisition and 1 kV to MS/MS). Obtained spectra were stored into the ABI 4000 Series Explorer Spot Set Manager, peptide mass fingerprint (PMF) and MS/MS fragment ion spectra were smoothed and corrected to zero baselines. Protein identification was performed by peptide fingerprint combined with MS/MS using the MASCOT version 2.3 search engine via Global Protein Server (GPS) version 3.6 (ABSCIEX), using SwissProt (553,231 sequences; 197,953,409 residues) and NCBInr databases (17,919,084 sequences; 6,150,218,869 residues) with taxonomic restriction to Homo sapiens (19,179 sequences) and *Plasmodium falciparum* (19,026 sequences), respectively. Search parameters included: trypsin and carbamidomethylation of cysteines as a fixed modification, oxidation of methionines as a variable modification, error tolerance for the precursor mass of 50 to 100 ppm and tolerance in the masses of the MS (MS) of 0.3 Da.

The identified proteins exceeded the score estimated by the MASCOT MOWSE algorithm so that matches are not due to chance with a probability lower than 5% and, therefore, be statistically significant with a *p*-value < 0.05. The fragmented peptides also exceeded the individual ion score that determines MASCOT as significance with a *p*-value < 0.05 (See Supplementary Material 2).

## 5. Conclusions

Oxidative damage to the proteome of the 3D7 *P. falciparum* clone cultivated in vitro with erythrocytes harboring the sickle cell trait has been quantified. Protein carbonyl index was used as a marker to demonstrate that irreversible oxidative damage increase with the progressing of asexual cycle life in HbAA RBCs and how it is augmented remarkable at the ring stage for parasites cultured in HbAS erythrocytes. The data obtained were added to others previously described and indicate that the biochemical protective mechanisms operating through irreversible oxidative damage in the parasite proteome and confer advantages to HbAS occurred or began from the early ring stage. Finally, our results reinforced the hypothesis that such oxidative modifications did not appear to happen randomly, and the sickle cell trait affected mainly a small fraction of parasite proteins particularly sensitive to ROS, such as those identified and involved in processes of recognition, invasion of erythrocytes and stress response.

## Figures and Tables

**Table 1:** Carbonyl index of *P. falciparum* proteins at different asexual stages in HbAA and HbAS erythrocytes.

Condition	Patients	C.I. (nmol of Carbonyls/mg of Protein)		
		Rings	Trophozoites	Schizonts
HbAA	NC	12.7 ± 1.07	24.9 ± 0.83	21.4 ± 0.95
HbAS	ST	88.0 ± 2.10	84.9 ± 2.15	83.9 ± 2.23
	MC	62.6 ± 1.04	59.7 ± 0.92	55.6 ± 1.29
	MJ	53.1 ± 1.99	66.3 ± 2.56	56.3 ± 3.00

Carbonyl index (C.I.) values calculated and expressed as means ± SD. Dilutions 1/10 in PBS of parasite proteins were required in those samples with C.I.s upper than 18 nmol of carbonyls/mg of protein.

**Table 2:** Relative increasing in carbonyl index on the proteome of *P. falciparum* cultured in HbAS RBCs.

Condition	HbAS/HbAA	C.I. HbAS/CI HbAA		
		Rings	Trophozoites	Schizonts
HbAS	ST/NC	6.92	3.38	3.93
	MC/NC	4.92	2.38	2.60
	MJ/NC	4.18	2.64	2.64
Means ± SD		5.34 ± 1.42	2.80 ± 0.52	3.05 ± 0.75

C.I.s: Carbonyl indexes can be observed there, in all cases, carbonyl indexes of parasites proteome were augmented in erythrocytes with the sickle cell trait. Furthermore, during the ring stage was recorded the largest oxidative increment being 1.9 folds and 1.75 folds greater than reached in trophozoites and schizonts stages. Therefore, our data show a particular susceptibility to protein oxidative stress at an early asexual stage of rings in the presence of this severe malaria protector polymorphism.

**Table 3:** Proteins identified in carbonylated bands.

Band	Identification	Mass (Da)	Score	Name Submission	Biology Process
1.1	B3AT_HUMAN	102,013	130	Anion transport protein, band 3	Anion and ion transport
	BB_HUMAN	16,102	82	Hemoglobin subunit beta	Oxygen transport
	PF3D7_0501600	43,672	112	Rhoptry-associated protein 2	Entrance to the host cell
	gi|11125364	55,782	56	Protein disulfide isomerase (*Plasmodium falciparum*)	Protein folding, Cell redox homeostasis, and response to endoplasmic reticulum stress
1.2	B3AT_HUMAN	102,013	274	Anion transport protein, Band 3	Anion and ion transport
2	HBB_HUMAN	16,102	201	Hemoglobin subunit beta	Oxygen transport
	gi|160112	33,774	62	β-galactosidase, partial (*P. falciparum*)	Metabolic process of cellular carbohydrates Catabolic galactose process
	gi|160537	27,813	64	Surface protein (*P. falciparum*)	Pathogenesis
	gi|124512406	74,382	57	Heat shock 70 kDa protein (*P. falciparum* 3D7)	Stress response, ATP metabolic process, cellular response to oxidative stress

## Supplementary Materials

Supplementary Materials can be found at https://www.mdpi.com/1422-0067/20/22/5769/s1.
