# Supplementary material for: Sickle Cell Trait Induces Oxidative Damage on Plasmodium falciparum Proteome at Erythrocyte Stages
Source: Int J Mol Sci. 2019 Nov 16;20(22):5769. doi: 10.3390/ijms20225769 (PMC6888313; doi:10.3390/ijms20225769)
Supplement: Supplementary file 1 [file ijms-20-05769-s001.zip › ijms-590016 suppl for final/ijms-590016 suppl2 proof done.pdf]

Supplementary Material 2
Results of search in Mascot for Peptide Mass Fingerprint (PMF) and MS/MS Data

Sickle cell trait induce oxidative damage on *Plasmodium falciparum* proteome at erythrocyte stages.

Alber Díaz-Castillo[a], Neyder Contreras-Puentes[b], Ciro Alvear-Sedán[c], Carlos Moneriz-Pretell[c], Erika Rodríguez-Cavallo[b*] and Darío Méndez-Cuadro[a*]

[a] Analytical Chemistry and Biomedicine Group. Exacts and Natural Sciences Faculty. University of Cartagena. Cartagena-Colombia.
[b] Analytical Chemistry and Biomedicine Group. Pharmaceuticals Sciences Faculty. University of Cartagena.
[c] Biochemistry and disease Group. Medicine School. University of Cartagena.

* Corresponding authors:
Darío Mendez-Cuadro. **Mailing address**: University of Cartagena, Faculty of Exact and Natural Sciences, Campus of San Pablo. Analytical Chemistry and Biomedicine Group, first floor No.109. Cra. 50 #24120, Cartagena, Colombia, AP 130015. **Phone number:** + 57 3015584887. **E-mail**: dmendezc@unicartagena.edu.co
Erika Rodríguez-Cavallo. **Mailing address**: University of Cartagena, Faculty of Pharmaceutical Sciences, Campus of Zaragocilla ancient building Cread, Analytical Chemistry and Biomedicine Group, first floor No.103. Cra. 50 #24120, Cartagena, Colombia, AP 130015. **Phone number:** + 57 3015588298. **E-mail**: erodriguezc1@unicartagena.edu.co

RUNNING HEAD

Oxidative damage on *Plasmodium falciparum* proteome

Mascot Search Results

**Band**	: **1.1**
User	:
Email	:
Search title	: SampleSetID: 759, AnalysisID: 6308, MaldiWellID: 66567, SpectrumID: 144105, Path=\171004\MSMS\17-112 NCBI alveolata run 79 y 72
Database	: NCBInr 20120508 (17919084 sequences; 6150218869 residues)
Taxonomy	: Plasmodium falciparum (malaria parasite) (19026 sequences)
Timestamp	: 30 Nov 2017 at 07:08:39 GMT
Warning	: A Peptide summary report will usually give a much clearer picture of MS/MS search results.
Top Score	: 112 for gi|237665430, rhoptry-associated protein 2 [Plasmodium falciparum]

Mascot Score Histogram

Protein score is -10*Log(P), where P is the probability that the observed match is a random event.
Protein scores greater than 55 are significant (p<0.05).
Protein scores are derived from ions scores as a non-probabilistic basis for ranking protein hits.

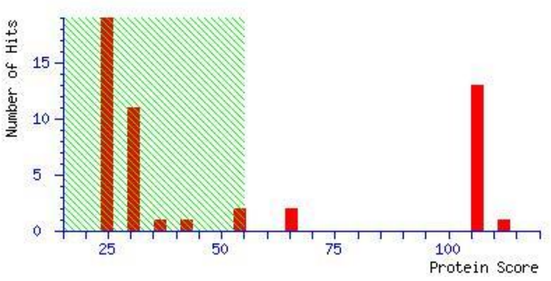

Protein Summary Report

Format As | Protein Summary (deprecated) | Help

Significance threshold p< 0.05 Max. number of hits 20

Re-Search All | Search Unmatched

Index

Accession	Mass	Score	Description
1. gi|237665430	43672	112	rhoptry-associated protein 2 [Plasmodium falciparum]
2. gi|221326632	41710	105	rhoptry-associated protein 2 [Plasmodium falciparum]
3. gi|221326634	41683	105	rhoptry-associated protein 2 [Plasmodium falciparum]
4. gi|221326636	41687	105	rhoptry-associated protein 2 [Plasmodium falciparum]
5. gi|221326638	41660	105	rhoptry-associated protein 2 [Plasmodium falciparum]
6. gi|237665418	41751	105	rhoptry-associated protein 2 [Plasmodium falciparum]
7. gi|237665432	41919	105	rhoptry-associated protein 2 [Plasmodium falciparum]
8. gi|237665420	42515	105	rhoptry-associated protein 2 [Plasmodium falciparum]
9. gi|237665422	42939	105	rhoptry-associated protein 2 [Plasmodium falciparum]
10. gi|237665426	43018	105	rhoptry-associated protein 2 [Plasmodium falciparum]
11. gi|237665424	43619	105	rhoptry-associated protein 2 [Plasmodium falciparum]
**12. gi|6118349**	**47017**	**104**	**rhoptry-associated protein 2 [Plasmodium falciparum]**
13. gi|124505955	46994	104	rhoptry-associated protein 2, RAP2 [Plasmodium falciparum 3D7]
14. gi|6683937	47051	104	rhoptry-associated protein 2 [Plasmodium falciparum]
15. gi|10961	47186	66	RAP-2 [Plasmodium falciparum]
16. gi|237665428	42421	64	rhoptry-associated protein 2 [Plasmodium falciparum]
**17. gi|11125364**	**55782**	**56**	**protein disulfide isomerase [Plasmodium falciparum]**
18. gi|296005096	55821	56	protein disulfide isomerase [Plasmodium falciparum 3D7]
**19. gi|3036869**	**354977**	**42**	**NAD(P)H-dependent glutamate synthase [Plasmodium falciparum]**
20. gi|124512690	195125	35	conserved Plasmodium protein, unknown function [Plasmodium falciparum 3D7]

Results List

1. gi|237665430 **Mass:** 43672 **Score:** 112 **Expect:** 1.2e-007 **Matches:** 6

rhoptry-associated protein 2 [Plasmodium falciparum]

Observed	Mr(expt)	Mr(calc)	ppm	Start		End	Miss	Ions	Peptide
1139.5815	1138.5742	1138.5771	-2.53	155	-	164	0	---	K.SNPYFIVGSR.V
1139.5815	1138.5742	1138.5771	-2.53	155	-	164	0	38	K.SNPYFIVGSR.V
1318.6443	1317.6370	1317.6353	1.29	190	-	200	0	---	R.DYNFLIYAGSR.E
1318.6443	1317.6370	1317.6353	1.29	190	-	200	0	57	R.DYNFLIYAGSR.E
2196.0725	2195.0652	2195.0674	-1.00	255	-	273	0	---	K.SEYYGTPDDLITSFFSIIK.T
2566.2903	2565.2830	2565.3339	-19.85	1	-	23	1	---	-.SKLYPESNSLTGLIYAHTAHVHK.L

No match to: 1034.1162, 1050.0874, 1267.6982, 1267.6982, 1274.7203, 1274.7317, 1384.7184, 1384.7184, 1445.7601, 1479.7841, 1529.7374, 1563.7852, 1563.7852, 1567.7542, 1575.8081, 1638.8492, 1759.9475, 1759.9475, 1787.9674, 1790.9246, 1790.9246, 1815.9518, 1858.9628, 1877.0386, 1877.0386, 1881.0592, 1905.0511, 1906.8796, 1951.9500, 1971.0089, 1993.0059, 1993.0059, 1994.0392, 1998.9767, 2008.9969, 2042.1071, 2074.9429, 2107.2236, 2117.0906, 2138.1675, 2139.1616, 2185.0957, 2194.0574, 2202.1440, 2204.1467, 2219.1174, 2225.1460, 2228.1523, 2235.2080, 2251.1221, 2268.1711, 2282.1650, 2286.2263, 2286.2263, 2314.1519, 2325.2527, 2327.2517, 2330.1921, 2332.1497, 2501.2480, 2586.2400, 2612.1943, 2705.4258, 2726.3000, 2925.5957, 3052.6226, 3136.4932, 3153.4534, 3206.5691, 3211.4873, 3224.5742

2. gi|221326632 **Mass:** 41710 **Score:** 105 **Expect:** 6e-007 **Matches:** 5

rhoptry-associated protein 2 [Plasmodium falciparum]

Observed	Mr(expt)	Mr(calc)	ppm	Start	End	Miss	Ions	Peptide
1139.5815	1138.5742	1138.5771	-2.53	151	160	0	---	K.SNPYFIVGSR.V
1139.5815	1138.5742	1138.5771	-2.53	151	160	0	38	K.SNPYFIVGSR.V
1318.6443	1317.6370	1317.6353	1.29	186	196	0	---	R.DYNFLIYAGSR.E
1318.6443	1317.6370	1317.6353	1.29	186	196	0	57	R.DYNFLIYAGSR.E
2196.0725	2195.0652	2195.0674	-1.00	251	269	0	---	K.SEYYGTPDDLITSFFSIIK.T

No match to: 1034.1162, 1050.0874, 1267.6982, 1267.6982, 1274.7203, 1274.7317, 1384.7184, 1384.7184, 1445.7601, 1479.7841, 1529.7374, 1563.7852, 1563.7852, 1567.7542, 1575.8081, 1638.8492, 1759.9475, 1759.9475, 1787.9674, 1790.9246, 1790.9246, 1815.9518, 1858.9628, 1877.0386, 1877.0386, 1881.0592, 1905.0511, 1906.8796, 1951.9500, 1971.0089, 1993.0059, 1993.0059, 1994.0392, 1998.9767, 2008.9969, 2042.1071, 2074.9429, 2107.2236, 2117.0906, 2138.1675, 2139.1616, 2185.0957, 2194.0574, 2202.1440, 2204.1467, 2219.1174, 2225.1460, 2228.1523, 2235.2080, 2251.1221, 2268.1711, 2282.1650, 2286.2263, 2286.2263, 2314.1519, 2325.2527, 2327.2517, 2330.1921, 2332.1497, 2501.2480, 2566.2903, 2586.2400, 2612.1943, 2705.4258, 2726.3000, 2925.5957, 3052.6226, 3136.4932, 3153.4534, 3206.5691, 3211.4873, 3224.5742

3. gi|221326634 **Mass:** 41683 **Score:** 105 **Expect:** 6e-007 **Matches:** 5

rhoptry-associated protein 2 [Plasmodium falciparum]

Observed	Mr(expt)	Mr(calc)	ppm	Start	End	Miss	Ions	Peptide
1139.5815	1138.5742	1138.5771	-2.53	151	160	0	---	K.SNPYFIVGSR.V
1139.5815	1138.5742	1138.5771	-2.53	151	160	0	38	K.SNPYFIVGSR.V
1318.6443	1317.6370	1317.6353	1.29	186	196	0	---	R.DYNFLIYAGSR.E
1318.6443	1317.6370	1317.6353	1.29	186	196	0	57	R.DYNFLIYAGSR.E
2196.0725	2195.0652	2195.0674	-1.00	251	269	0	---	K.SEYYGTPDDLITSFFSIIK.T

No match to: 1034.1162, 1050.0874, 1267.6982, 1267.6982, 1274.7203, 1274.7317, 1384.7184, 1384.7184, 1445.7601, 1479.7841, 1529.7374, 1563.7852, 1563.7852, 1567.7542, 1575.8081, 1638.8492, 1759.9475, 1759.9475, 1787.9674, 1790.9246, 1790.9246, 1815.9518, 1858.9628, 1877.0386, 1877.0386, 1881.0592, 1905.0511, 1906.8796, 1951.9500, 1971.0089, 1993.0059, 1993.0059, 1994.0392, 1998.9767, 2008.9969, 2042.1071, 2074.9429, 2107.2236, 2117.0906, 2138.1675, 2139.1616, 2185.0957, 2194.0574, 2202.1440, 2204.1467, 2219.1174, 2225.1460, 2228.1523, 2235.2080, 2251.1221, 2268.1711, 2282.1650, 2286.2263, 2286.2263, 2314.1519, 2325.2527, 2327.2517, 2330.1921, 2332.1497, 2501.2480, 2566.2903, 2586.2400, 2612.1943, 2705.4258, 2726.3000, 2925.5957, 3052.6226, 3136.4932, 3153.4534, 3206.5691, 3211.4873, 3224.5742

4. gi|221326636 **Mass:** 41687 **Score:** 105 **Expect:** 6e-007 **Matches:** 5

rhoptry-associated protein 2 [Plasmodium falciparum]

Observed	Mr(expt)	Mr(calc)	ppm	Start	End	Miss	Ions	Peptide
1139.5815	1138.5742	1138.5771	-2.53	151	160	0	---	K.SNPYFIVGSR.V
1139.5815	1138.5742	1138.5771	-2.53	151	160	0	38	K.SNPYFIVGSR.V
1318.6443	1317.6370	1317.6353	1.29	186	196	0	---	R.DYNFLIYAGSR.E
1318.6443	1317.6370	1317.6353	1.29	186	196	0	57	R.DYNFLIYAGSR.E
2196.0725	2195.0652	2195.0674	-1.00	251	269	0	---	K.SEYYGTPDDLITSFFSIIK.T

No match to: 1034.1162, 1050.0874, 1267.6982, 1267.6982, 1274.7203, 1274.7317, 1384.7184, 1384.7184, 1445.7601, 1479.7841, 1529.7374, 1563.7852, 1563.7852, 1567.7542, 1575.8081, 1638.8492, 1759.9475, 1759.9475, 1787.9674, 1790.9246, 1790.9246, 1815.9518, 1858.9628, 1877.0386, 1877.0386, 1881.0592, 1905.0511, 1906.8796, 1951.9500, 1971.0089, 1993.0059, 1993.0059, 1994.0392, 1998.9767, 2008.9969, 2042.1071, 2074.9429, 2107.2236, 2117.0906, 2138.1675, 2139.1616, 2185.0957, 2194.0574, 2202.1440, 2204.1467, 2219.1174, 2225.1460, 2228.1523, 2235.2080, 2251.1221, 2268.1711, 2282.1650, 2286.2263, 2286.2263, 2314.1519, 2325.2527, 2327.2517, 2330.1921, 2332.1497, 2501.2480, 2566.2903, 2586.2400, 2612.1943, 2705.4258, 2726.3000, 2925.5957, 3052.6226, 3136.4932, 3153.4534, 3206.5691, 3211.4873, 3224.5742

5. gi|221326638 **Mass:** 41660 **Score:** 105 **Expect:** 6e-007 **Matches:** 5

rhoptry-associated protein 2 [Plasmodium falciparum]

Observed	Mr(expt)	Mr(calc)	ppm	Start	End	Miss	Ions	Peptide
1139.5815	1138.5742	1138.5771	-2.53	151	160	0	---	K.SNPYFIVGSR.V
1139.5815	1138.5742	1138.5771	-2.53	151	160	0	38	K.SNPYFIVGSR.V
1318.6443	1317.6370	1317.6353	1.29	186	196	0	---	R.DYNFLIYAGSR.E
1318.6443	1317.6370	1317.6353	1.29	186	196	0	57	R.DYNFLIYAGSR.E
2196.0725	2195.0652	2195.0674	-1.00	251	269	0	---	K.SEYYGTPDDLITSFFSIIK.T

No match to: 1034.1162, 1050.0874, 1267.6982, 1267.6982, 1274.7203, 1274.7317, 1384.7184, 1384.7184, 1445.7601, 1479.7841, 1529.7374, 1563.7852, 1563.7852, 1567.7542, 1575.8081, 1638.8492, 1759.9475, 1759.9475, 1787.9674, 1790.9246, 1790.9246, 1815.9518, 1858.9628, 1877.0386, 1877.0386, 1881.0592, 1905.0511, 1906.8796, 1951.9500, 1971.0089, 1993.0059, 1993.0059, 1994.0392, 1998.9767, 2008.9969, 2042.1071, 2074.9429, 2107.2236, 2117.0906, 2138.1675, 2139.1616, 2185.0957, 2194.0574, 2202.1440, 2204.1467, 2219.1174, 2225.1460, 2228.1523, 2235.2080, 2251.1221, 2268.1711, 2282.1650, 2286.2263, 2286.2263, 2314.1519, 2325.2527, 2327.2517, 2330.1921, 2332.1497, 2501.2480, 2566.2903, 2586.2400, 2612.1943, 2705.4258, 2726.3000, 2925.5957, 3052.6226, 3136.4932, 3153.4534, 3206.5691, 3211.4873, 3224.5742

6. gi|237665418 **Mass:** 41751 **Score:** 105 **Expect:** 6e-007 **Matches:** 5

rhoptry-associated protein 2 [Plasmodium falciparum]

Observed	Mr(expt)	Mr(calc)	ppm	Start	End	Miss	Ions	Peptide
1139.5815	1138.5742	1138.5771	-2.53	147	156	0	---	K.SNPYFIVGSR.V
1139.5815	1138.5742	1138.5771	-2.53	147	156	0	38	K.SNPYFIVGSR.V
1318.6443	1317.6370	1317.6353	1.29	182	192	0	---	R.DYNFLIYAGSR.E
1318.6443	1317.6370	1317.6353	1.29	182	192	0	57	R.DYNFLIYAGSR.E
2196.0725	2195.0652	2195.0674	-1.00	247	265	0	---	K.SEYYGTPDDLITSFFSIIK.T

No match to: 1034.1162, 1050.0874, 1267.6982, 1267.6982, 1274.7203, 1274.7317, 1384.7184, 1384.7184, 1445.7601, 1479.7841, 1529.7374, 1563.7852, 1563.7852, 1567.7542, 1575.8081, 1638.8492, 1759.9475, 1759.9475, 1787.9674, 1790.9246, 1790.9246, 1815.9518, 1858.9628, 1877.0386, 1877.0386, 1881.0592, 1905.0511, 1906.8796, 1951.9500, 1971.0089, 1993.0059, 1993.0059, 1994.0392, 1998.9767, 2008.9969, 2042.1071, 2074.9429, 2107.2236, 2117.0906, 2138.1675, 2139.1616, 2185.0957, 2194.0574, 2202.1440, 2204.1467, 2219.1174, 2225.1460, 2228.1523, 2235.2080, 2251.1221, 2268.1711, 2282.1650, 2286.2263, 2286.2263, 2314.1519, 2325.2527, 2327.2517, 2330.1921, 2332.1497, 2501.2480, 2566.2903, 2586.2400, 2612.1943, 2705.4258, 2726.3000, 2925.5957, 3052.6226, 3136.4932, 3153.4534, 3206.5691, 3211.4873, 3224.5742

7. gi|237665432 **Mass:** 41919 **Score:** 105 **Expect:** 6e-007 **Matches:** 5

rhoptry-associated protein 2 [Plasmodium falciparum]

Observed	Mr(expt)	Mr(calc)	ppm	Start	End	Miss	Ions	Peptide
1139.5815	1138.5742	1138.5771	-2.53	146	155	0	---	K.SNPYFIVGSR.V

1139.5815	1138.5742	1138.5771	-2.53	146	- 155	0	38	K.SNPYFIVGSR.V
1318.6443	1317.6370	1317.6353	1.29	181	- 191	0	---	R.DYNFLIYAGSR.E
1318.6443	1317.6370	1317.6353	1.29	181	- 191	0	57	R.DYNFLIYAGSR.E
2196.0725	2195.0652	2195.0674	-1.00	246	- 264	0	---	K.SEYYGTPDDLITSFFSIIK.T

No match to: 1034.1162, 1050.0874, 1267.6982, 1267.6982, 1274.7203, 1274.7317, 1384.7184, 1384.7184, 1445.7601, 1479.7841, 1529.7374, 1563.7852, 1563.7852, 1567.7542, 1575.8081, 1638.8492, 1759.9475, 1759.9475, 1787.9674, 1790.9246, 1790.9246, 1815.9518, 1858.9628, 1877.0386, 1877.0386, 1881.0592, 1905.0511, 1906.8796, 1951.9500, 1971.0089, 1993.0059, 1993.0059, 1994.0392, 1998.9767, 2008.9969, 2042.1071, 2074.9429, 2107.2236, 2117.0906, 2138.1675, 2139.1616, 2185.0957, 2194.0574, 2202.1440, 2204.1467, 2219.1174, 2225.1460, 2228.1523, 2235.2080, 2251.1221, 2268.1711, 2282.1650, 2286.2263, 2286.2263, 2314.1519, 2325.2527, 2327.2517, 2330.1921, 2332.1497, 2501.2480, 2566.2903, 2586.2400, 2612.1943, 2705.4258, 2726.3000, 2925.5957, 3052.6226, 3136.4932, 3153.4534, 3206.5691, 3211.4873, 3224.5742

8. gi|237665420 **Mass:** 42515 **Score:** 105 **Expect:** 6e-007 **Matches:** 5

rhoptry-associated protein 2 [Plasmodium falciparum]

Observed	Mr(expt)	Mr(calc)	ppm	Start	End	Miss	Ions	Peptide
1139.5815	1138.5742	1138.5771	-2.53	149-	158	0	---	K.SNPYFIVGSR.V
1139.5815	1138.5742	1138.5771	-2.53	149-	158	0	38	K.SNPYFIVGSR.V
1318.6443	1317.6370	1317.6353	1.29	184-	194	0	---	R.DYNFLIYAGSR.E
1318.6443	1317.6370	1317.6353	1.29	184-	194	0	57	R.DYNFLIYAGSR.E
2196.0725	2195.0652	2195.0674	-1.00	249-	267	0	---	K.SEYYGTPDDLITSFFSIIK.T

No match to: 1034.1162, 1050.0874, 1267.6982, 1267.6982, 1274.7203, 1274.7317, 1384.7184, 1384.7184, 1445.7601, 1479.7841, 1529.7374, 1563.7852, 1563.7852, 1567.7542, 1575.8081, 1638.8492, 1759.9475, 1759.9475, 1787.9674, 1790.9246, 1790.9246, 1815.9518, 1858.9628, 1877.0386, 1877.0386, 1881.0592, 1905.0511, 1906.8796, 1951.9500, 1971.0089, 1993.0059, 1993.0059, 1994.0392, 1998.9767, 2008.9969, 2042.1071, 2074.9429, 2107.2236, 2117.0906, 2138.1675, 2139.1616, 2185.0957, 2194.0574, 2202.1440, 2204.1467, 2219.1174, 2225.1460, 2228.1523, 2235.2080, 2251.1221, 2268.1711, 2282.1650, 2286.2263, 2286.2263, 2314.1519, 2325.2527, 2327.2517, 2330.1921, 2332.1497, 2501.2480, 2566.2903, 2586.2400, 2612.1943, 2705.4258, 2726.3000, 2925.5957, 3052.6226, 3136.4932, 3153.4534, 3206.5691, 3211.4873, 3224.5742

9. gi|237665422 **Mass:** 42939 **Score:** 105 **Expect:** 6e-007 **Matches:** 5

rhoptry-associated protein 2 [Plasmodium falciparum]

Observed	Mr(expt)	Mr(calc)	ppm	Start	End	Miss	Ions	Peptide
1139.5815	1138.5742	1138.5771	-2.53	152-	161	0	---	K.SNPYFIVGSR.V
1139.5815	1138.5742	1138.5771	-2.53	152-	161	0	38	K.SNPYFIVGSR.V
1318.6443	1317.6370	1317.6353	1.29	187-	197	0	---	R.DYNFLIYAGSR.E
1318.6443	1317.6370	1317.6353	1.29	187-	197	0	57	R.DYNFLIYAGSR.E
2196.0725	2195.0652	2195.0674	-1.00	252-	270	0	---	K.SEYYGTPDDLITSFFSIIK.T

No match to: 1034.1162, 1050.0874, 1267.6982, 1267.6982, 1274.7203, 1274.7317, 1384.7184, 1384.7184, 1445.7601, 1479.7841, 1529.7374, 1563.7852, 1563.7852, 1567.7542, 1575.8081, 1638.8492, 1759.9475, 1759.9475, 1787.9674, 1790.9246, 1790.9246, 1815.9518, 1858.9628, 1877.0386, 1877.0386, 1881.0592, 1905.0511, 1906.8796, 1951.9500, 1971.0089, 1993.0059, 1993.0059, 1994.0392, 1998.9767, 2008.9969, 2042.1071, 2074.9429, 2107.2236, 2117.0906, 2138.1675, 2139.1616, 2185.0957, 2194.0574, 2202.1440, 2204.1467, 2219.1174, 2225.1460, 2228.1523, 2235.2080, 2251.1221, 2268.1711, 2282.1650, 2286.2263, 2286.2263, 2314.1519, 2325.2527, 2327.2517, 2330.1921, 2332.1497, 2501.2480, 2566.2903, 2586.2400, 2612.1943, 2705.4258, 2726.3000, 2925.5957, 3052.6226, 3136.4932, 3153.4534, 3206.5691, 3211.4873, 3224.5742

10. gi|237665426 **Mass:** 43018 **Score:** 105 **Expect:** 6e-007 **Matches:** 5

rhoptry-associated protein 2 [Plasmodium falciparum]

Observed	Mr(expt)	Mr(calc)	ppm	Start	End	Miss	Ions	Peptide
1139.5815	1138.5742	1138.5771	-2.53	152-	161	0	---	K.SNPYFIVGSR.V
1139.5815	1138.5742	1138.5771	-2.53	152-	161	0	38	K.SNPYFIVGSR.V
1318.6443	1317.6370	1317.6353	1.29	187-	197	0	---	R.DYNFLIYAGSR.E
1318.6443	1317.6370	1317.6353	1.29	187-	197	0	57	R.DYNFLIYAGSR.E
2196.0725	2195.0652	2195.0674	-1.00	252-	270	0	---	K.SEYYGTPDDLITSFFSIIK.T

No match to: 1034.1162, 1050.0874, 1267.6982, 1267.6982, 1274.7203, 1274.7317, 1384.7184, 1384.7184, 1445.7601, 1479.7841, 1529.7374, 1563.7852, 1563.7852, 1567.7542, 1575.8081, 1638.8492, 1759.9475, 1759.9475, 1787.9674, 1790.9246, 1790.9246, 1815.9518, 1858.9628, 1877.0386, 1877.0386, 1881.0592, 1905.0511, 1906.8796, 1951.9500, 1971.0089, 1993.0059, 1993.0059, 1994.0392, 1998.9767, 2008.9969, 2042.1071, 2074.9429, 2107.2236, 2117.0906, 2138.1675, 2139.1616, 2185.0957, 2194.0574, 2202.1440, 2204.1467, 2219.1174, 2225.1460, 2228.1523, 2235.2080, 2251.1221, 2268.1711, 2282.1650, 2286.2263, 2286.2263, 2314.1519, 2325.2527, 2327.2517, 2330.1921, 2332.1497, 2501.2480, 2566.2903, 2586.2400, 2612.1943, 2705.4258, 2726.3000, 2925.5957, 3052.6226, 3136.4932, 3153.4534, 3206.5691, 3211.4873, 3224.5742

11. gi|237665424 **Mass:** 43619 **Score:** 105 **Expect:** 6e-007 **Matches:** 5

rhoptry-associated protein 2 [Plasmodium falciparum]

Observed	Mr(expt)	Mr(calc)	ppm	Start	End	Miss	Ions	Peptide
1139.5815	1138.5742	1138.5771	-2.53	154-	163	0	---	K.SNPYFIVGSR.V
1139.5815	1138.5742	1138.5771	-2.53	154-	163	0	38	K.SNPYFIVGSR.V
1318.6443	1317.6370	1317.6353	1.29	189-	199	0	---	R.DYNFLIYAGSR.E
1318.6443	1317.6370	1317.6353	1.29	189-	199	0	57	R.DYNFLIYAGSR.E
2196.0725	2195.0652	2195.0674	-1.00	254-	272	0	---	K.SEYYGTPDDLITSFFSIIK.T

No match to: 1034.1162, 1050.0874, 1267.6982, 1267.6982, 1274.7203, 1274.7317, 1384.7184, 1384.7184, 1445.7601, 1479.7841, 1529.7374, 1563.7852, 1563.7852, 1567.7542, 1575.8081, 1638.8492, 1759.9475, 1759.9475, 1787.9674, 1790.9246, 1790.9246, 1815.9518, 1858.9628, 1877.0386, 1877.0386, 1881.0592, 1905.0511, 1906.8796, 1951.9500, 1971.0089, 1993.0059, 1993.0059, 1994.0392, 1998.9767, 2008.9969, 2042.1071, 2074.9429, 2107.2236, 2117.0906, 2138.1675, 2139.1616, 2185.0957, 2194.0574, 2202.1440, 2204.1467, 2219.1174, 2225.1460, 2228.1523, 2235.2080, 2251.1221, 2268.1711, 2282.1650, 2286.2263, 2286.2263, 2314.1519, 2325.2527, 2327.2517, 2330.1921, 2332.1497, 2501.2480, 2566.2903, 2586.2400, 2612.1943, 2705.4258, 2726.3000, 2925.5957, 3052.6226, 3136.4932, 3153.4534, 3206.5691, 3211.4873, 3224.5742

12. gi|6118349 **Mass:** 47017 **Score:** 104 **Expect:** 7.6e-007 **Matches:** 5

rhoptry-associated protein 2 [Plasmodium falciparum]

Observed	Mr(expt)	Mr(calc)	ppm	Start		End	Miss	Ions	Peptide
1139.5815	1138.5742	1138.5771	-2.53	183	-	192	0	---	K.SNPYFIVGSR.V
1139.5815	1138.5742	1138.5771	-2.53	183	-	192	0	38	K.SNPYFIVGSR.V
1318.6443	1317.6370	1317.6353	1.29	218	-	228	0	---	R.DYNFLIYAGSR.E
1318.6443	1317.6370	1317.6353	1.29	218	-	228	0	57	R.DYNFLIYAGSR.E

2196.0725	2195.0652	2195.0674	-1.00	283	301	0	---	K.SEYYGTPDDLITSFFSIIK.T

No match to: 1034.1162, 1050.0874, 1267.6982, 1267.6982, 1274.7203, 1274.7317, 1384.7184, 1384.7184, 1445.7601, 1479.7841, 1529.7374, 1563.7852, 1563.7852, 1567.7542, 1575.8081, 1638.8492, 1759.9475, 1759.9475, 1787.9674, 1790.9246, 1790.9246, 1815.9518, 1858.9628, 1877.0386, 1877.0386, 1881.0592, 1905.0511, 1906.8796, 1951.9500, 1971.0089, 1993.0059, 1993.0059, 1994.0392, 1998.9767, 2008.9969, 2042.1071, 2074.9429, 2107.2236, 2117.0906, 2138.1675, 2139.1616, 2185.0957, 2194.0574, 2202.1440, 2204.1467, 2219.1174, 2225.1460, 2228.1523, 2235.2080, 2251.1221, 2268.1711, 2282.1650, 2286.2263, 2286.2263, 2314.1519, 2325.2527, 2327.2517, 2330.1921, 2332.1497, 2501.2480, 2566.2903, 2586.2400, 2612.1943, 2705.4258, 2726.3000, 2925.5957, 3052.6226, 3136.4932, 3153.4534, 3206.5691, 3211.4873, 3224.5742

13. gi|124505955 **Mass:** 46994 **Score:** 104 **Expect:** 7.6e-007 **Matches:** 5

rhoptry-associated protein 2, RAP2 [Plasmodium falciparum 3D7]

Observed	Mr(expt)	Mr(calc)	ppm	Start	End	Miss	Ions	Peptide
1139.5815	1138.5742	1138.5771	-2.53	183	192	0	---	K.SNPYFIVGSR.V
1139.5815	1138.5742	1138.5771	-2.53	183	192	0	38	K.SNPYFIVGSR.V
1318.6443	1317.6370	1317.6353	1.29	218	228	0	---	R.DYNFLIYAGSR.E
1318.6443	1317.6370	1317.6353	1.29	218	228	0	57	R.DYNFLIYAGSR.E
2196.0725	2195.0652	2195.0674	-1.00	283	301	0	---	K.SEYYGTPDDLITSFFSIIK.T

No match to: 1034.1162, 1050.0874, 1267.6982, 1267.6982, 1274.7203, 1274.7317, 1384.7184, 1384.7184, 1445.7601, 1479.7841, 1529.7374, 1563.7852, 1563.7852, 1567.7542, 1575.8081, 1638.8492, 1759.9475, 1759.9475, 1787.9674, 1790.9246, 1790.9246, 1815.9518, 1858.9628, 1877.0386, 1877.0386, 1881.0592, 1905.0511, 1906.8796, 1951.9500, 1971.0089, 1993.0059, 1993.0059, 1994.0392, 1998.9767, 2008.9969, 2042.1071, 2074.9429, 2107.2236, 2117.0906, 2138.1675, 2139.1616, 2185.0957, 2194.0574, 2202.1440, 2204.1467, 2219.1174, 2225.1460, 2228.1523, 2235.2080, 2251.1221, 2268.1711, 2282.1650, 2286.2263, 2286.2263, 2314.1519, 2325.2527, 2327.2517, 2330.1921, 2332.1497, 2501.2480, 2566.2903, 2586.2400, 2612.1943, 2705.4258, 2726.3000, 2925.5957, 3052.6226, 3136.4932, 3153.4534, 3206.5691, 3211.4873, 3224.5742

14. gi|6683937 **Mass:** 47051 **Score:** 104 **Expect:** 7.6e-007 **Matches:** 5

rhoptry-associated protein 2 [Plasmodium falciparum]

Observed	Mr(expt)	Mr(calc)	ppm	Start	End	Miss	Ions	Peptide
1139.5815	1138.5742	1138.5771	-2.53	183	192	0	---	K.SNPYFIVGSR.V
1139.5815	1138.5742	1138.5771	-2.53	183	192	0	38	K.SNPYFIVGSR.V
1318.6443	1317.6370	1317.6353	1.29	218	228	0	---	R.DYNFLIYAGSR.E
1318.6443	1317.6370	1317.6353	1.29	218	228	0	57	R.DYNFLIYAGSR.E
2196.0725	2195.0652	2195.0674	-1.00	283	301	0	---	K.SEYYGTPDDLITSFFSIIK.T

No match to: 1034.1162, 1050.0874, 1267.6982, 1267.6982, 1274.7203, 1274.7317, 1384.7184, 1384.7184, 1445.7601, 1479.7841, 1529.7374, 1563.7852, 1563.7852, 1567.7542, 1575.8081, 1638.8492, 1759.9475, 1759.9475, 1787.9674, 1790.9246, 1790.9246, 1815.9518, 1858.9628, 1877.0386, 1877.0386, 1881.0592, 1905.0511, 1906.8796, 1951.9500, 1971.0089, 1993.0059, 1993.0059, 1994.0392, 1998.9767, 2008.9969, 2042.1071, 2074.9429, 2107.2236, 2117.0906, 2138.1675, 2139.1616, 2185.0957, 2194.0574, 2202.1440, 2204.1467, 2219.1174, 2225.1460, 2228.1523, 2235.2080, 2251.1221, 2268.1711, 2282.1650, 2286.2263, 2286.2263, 2314.1519, 2325.2527, 2327.2517, 2330.1921, 2332.1497, 2501.2480, 2566.2903, 2586.2400, 2612.1943, 2705.4258, 2726.3000, 2925.5957, 3052.6226, 3136.4932, 3153.4534, 3206.5691, 3211.4873, 3224.5742

15. gi|10961 **Mass:** 47186 **Score:** 66 **Expect:** 0.0046 **Matches:** 5

RAP-2 [Plasmodium falciparum]

Observed	Mr(expt)	Mr(calc)	ppm	Start	End	Miss	Ions	Peptide
1274.7203	1273.7130	1273.6567	44.2	183	192	1	---	K.YNRYFIVGSR.V
1274.7317	1273.7244	1273.6567	53.1	183	192	1	---	K.YNRYFIVGSR.V
1318.6443	1317.6370	1317.6353	1.29	218	228	0	---	R.DYNFLIYAGSR.E
1318.6443	1317.6370	1317.6353	1.29	218	228	0	57	R.DYNFLIYAGSR.E
2196.0725	2195.0652	2195.0674	-1.00	283	301	0	---	K.SEYYGTPDDLITSFFSIIK.T

No match to: 1034.1162, 1050.0874, 1139.5815, 1139.5815, 1267.6982, 1267.6982, 1384.7184, 1384.7184, 1445.7601, 1479.7841, 1529.7374, 1563.7852, 1563.7852, 1567.7542, 1575.8081, 1638.8492, 1759.9475, 1759.9475, 1787.9674, 1790.9246, 1790.9246, 1815.9518, 1858.9628, 1877.0386, 1877.0386, 1881.0592, 1905.0511, 1906.8796, 1951.9500, 1971.0089, 1993.0059, 1993.0059, 1994.0392, 1998.9767, 2008.9969, 2042.1071, 2074.9429, 2107.2236, 2117.0906, 2138.1675, 2139.1616, 2185.0957, 2194.0574, 2202.1440, 2204.1467, 2219.1174, 2225.1460, 2228.1523, 2235.2080, 2251.1221, 2268.1711, 2282.1650, 2286.2263, 2286.2263, 2314.1519, 2325.2527, 2327.2517, 2330.1921, 2332.1497, 2501.2480, 2566.2903, 2586.2400, 2612.1943, 2705.4258, 2726.3000, 2925.5957, 3052.6226, 3136.4932, 3153.4534, 3206.5691, 3211.4873, 3224.5742

16. gi|237665428 **Mass:** 42424 **Score:** 64 **Expect:** 0.0085 **Matches:** 3

rhoptry-associated protein 2 [Plasmodium falciparum]

Observed	Mr(expt)	Mr(calc)	ppm	Start	End	Miss	Ions	Peptide
1318.6443	1317.6370	1317.6353	1.29	182	192	0	---	R.DYNFLIYAGSR.E
1318.6443	1317.6370	1317.6353	1.29	182	192	0	57	R.DYNFLIYAGSR.E
2196.0725	2195.0652	2195.0674	-1.00	247	265	0	---	K.SEYYGTPDDLITSFFSIIK.T

No match to: 1034.1162, 1050.0874, 1139.5815, 1139.5815, 1267.6982, 1267.6982, 1274.7203, 1274.7317, 1384.7184, 1384.7184, 1445.7601, 1479.7841, 1529.7374, 1563.7852, 1563.7852, 1567.7542, 1575.8081, 1638.8492, 1759.9475, 1759.9475, 1787.9674, 1790.9246, 1790.9246, 1815.9518, 1858.9628, 1877.0386, 1877.0386, 1881.0592, 1905.0511, 1906.8796, 1951.9500, 1971.0089, 1993.0059, 1993.0059, 1994.0392, 1998.9767, 2008.9969, 2042.1071, 2074.9429, 2107.2236, 2117.0906, 2138.1675, 2139.1616, 2185.0957, 2194.0574, 2202.1440, 2204.1467, 2219.1174, 2225.1460, 2228.1523, 2235.2080, 2251.1221, 2268.1711, 2282.1650, 2286.2263, 2286.2263, 2314.1519, 2325.2527, 2327.2517, 2330.1921, 2332.1497, 2501.2480, 2566.2903, 2586.2400, 2612.1943, 2705.4258, 2726.3000, 2925.5957, 3052.6226, 3136.4932, 3153.4534, 3206.5691, 3211.4873, 3224.5742

17. gi|11125364 **Mass:** 55782 **Score:** 56 **Expect:** 0.047 **Matches:** 12

protein disulfide isomerase [Plasmodium falciparum]

Observed	Mr(expt)	Mr(calc)	ppm	Start	End	Miss	Ions	Peptide
1267.6982	1266.6909	1266.6972	-4.97	450	460	1	---	R.SLKGFVDFLNK.H
1267.6982	1266.6909	1266.6972	-4.97	450	460	1	---	R.SLKGFVDFLNK.H
1318.6443	1317.6370	1317.6928	-42.36	390	400	1	---	K.LEPVYEDLGRK.L
1318.6443	1317.6370	1317.6928	-42.36	389	399	1	---	K.KLEPVYEDLGR.K
1877.0386	1876.0313	1875.9400	48.7	66	81	1	---	R.LIPEYNEAANMLNEKK.S
1877.0386	1876.0313	1875.9400	48.7	66	81	1	---	R.LIPEYNEAANMLNEKK.S
1905.0511	1904.0438	1903.9461	51.3	65	80	1	---	K.RLIPEYNEAANMLNEK.K
1994.0392	1993.0319	1992.9905	20.8	292	310	0	---	K.ASLGLTEFPGLAFQSNEGR.Y
1998.9767	1997.9694	1998.0686	-49.66	315	331	1	---	K.NPKESLLNHNAIINFFK.D

2282.1650	2281.1577	2281.0595	43.1	48 -	65	1	---	K.NDIVLVMFYAPWCGHCKR.L + Oxidation (M)
2325.2527	2324.2454	2324.1212	53.4	157 -	175	1	---	K.INVAFYLEYTSEDNDLYKK.F
2566.2903	2565.2830	2565.2322	19.8	247 -	266	1	---	K.ELVWVCATYEQYNEIKEHVR.L

No match to: 1034.1162, 1050.0874, 1139.5815, 1139.5815, 1274.7203, 1274.7317, 1384.7184, 1384.7184, 1445.7601, 1479.7841, 1529.7374, 1563.7852, 1563.7852, 1567.7542, 1575.8081, 1638.8492, 1759.9475, 1759.9475, 1787.9674, 1790.9246, 1790.9246, 1815.9518, 1858.9628, 1881.0592, 1906.8796, 1951.9500, 1971.0089, 1993.0059, 1993.0059, 2008.9969, 2042.1071, 2074.9429, 2107.2236, 2117.0906, 2138.1675, 2139.1616, 2185.0957, 2194.0574, 2196.0725, 2202.1440, 2204.1467, 2219.1174, 2225.1460, 2228.1523, 2235.2080, 2251.1221, 2268.1711, 2286.2263, 2286.2263, 2314.1519, 2327.2517, 2330.1921, 2332.1497, 2501.2480, 2586.2400, 2612.1943, 2705.4258, 2726.3000, 2925.5957, 3052.6226, 3136.4932, 3153.4534, 3206.5691, 3211.4873, 3224.5742

18. gi|296005096 **Mass:** 55821 **Score:** 56 **Expect:** 0.047 **Matches:** 12

protein disulfide isomerase [Plasmodium falciparum 3D7]

Observed	Mr(expt)	Mr(calc)	ppm	Start	End	Miss	Ions	Peptide
1267.6982	1266.6909	1266.6972	-4.97	450	- 460	1	---	R.SLKGFVDFLNK.H
1267.6982	1266.6909	1266.6972	-4.97	450	- 460	1	---	R.SLKGFVDFLNK.H
1318.6443	1317.6370	1317.6928	-42.36	390	- 400	1	---	K.LEPVYEDLGRK.L
1318.6443	1317.6370	1317.6928	-42.36	389	- 399	1	---	K.KLEPVYEDLGR.K
1877.0386	1876.0313	1875.9400	48.7	66	- 81	1	---	R.LIPEYNEAANMLNEKK.S
1877.0386	1876.0313	1875.9400	48.7	66	- 81	1	---	R.LIPEYNEAANMLNEKK.S
1905.0511	1904.0438	1903.9461	51.3	65	- 80	1	---	K.RLIPEYNEAANMLNEK.K
1994.0392	1993.0319	1992.9905	20.8	292	- 310	0	---	K.ASLGLTEFPGLAFQSNEGR.Y
1998.9767	1997.9694	1998.0686	-49.66	315	- 331	1	---	K.NPKESLLNHNAIINFFK.D
2282.1650	2281.1577	2281.0595	43.1	48	- 65	1	---	K.NDIVLVMFYAPWCGHCKR.L + Oxidation (M)
2325.2527	2324.2454	2324.1212	53.4	157	- 175	1	---	K.INVAFYLEYTSEDNDLYKK.F
2566.2903	2565.2830	2565.2322	19.8	247	- 266	1	---	K.ELVWVCATYEQYNEIKEHVR.L

No match to: 1034.1162, 1050.0874, 1139.5815, 1139.5815, 1274.7203, 1274.7317, 1384.7184, 1384.7184, 1445.7601, 1479.7841, 1529.7374, 1563.7852, 1563.7852, 1567.7542, 1575.8081, 1638.8492, 1759.9475, 1759.9475, 1787.9674, 1790.9246, 1790.9246, 1815.9518, 1858.9628, 1881.0592, 1906.8796, 1951.9500, 1971.0089, 1993.0059, 1993.0059, 2008.9969, 2042.1071, 2074.9429, 2107.2236, 2117.0906, 2138.1675, 2139.1616, 2185.0957, 2194.0574, 2196.0725, 2202.1440, 2204.1467, 2219.1174, 2225.1460, 2228.1523, 2235.2080, 2251.1221, 2268.1711, 2286.2263, 2286.2263, 2314.1519, 2327.2517, 2330.1921, 2332.1497, 2501.2480, 2586.2400, 2612.1943, 2705.4258, 2726.3000, 2925.5957, 3052.6226, 3136.4932, 3153.4534, 3206.5691, 3211.4873, 3224.5742

Search Parameters

```
Type of search          :Sequence Query
Enzyme                  :Trypsin
Fixed modifications     :Carbamidomethyl (C)
Variable modifications  :Oxidation (M)
Mass values             :Monoisotopic
Protein Mass            :Unrestricted
Peptide  Mass Tolerance :± 80 ppm
Fragment Mass Tolerance:± 0.3 Da
Max Missed Cleavages    :1
Instrument type         :MALDI-TOF-TOF
 Query1 (1034.1162,1+)  :<no title>
 Query2 (1050.0874,1+)  :<no title>
 Query3 (1139.5815,1+)  :<no title>
 Query4 (1139.5815,1+)  :MaldiWellID: 66567, SpectrumID: 144114,
 Query5 (1267.6982,1+)  :<no title>
 Query6 (1267.6982,1+)  :MaldiWellID: 66567, SpectrumID: 144108,
 Query7 (1274.7203,1+)  :<no title>
 Query8 (1274.7317,1+)  :MaldiWellID: 66567, SpectrumID: 144111,
 Query9 (1318.6443,1+)  :<no title>
Query10 (1318.6443,1+)  :MaldiWellID: 66567, SpectrumID: 144115,
Query11 (1384.7184,1+)  :<no title>
Query12 (1384.7184,1+)  :MaldiWellID: 66567, SpectrumID: 144107,
Query13 (1445.7601,1+)  :<no title>
Query14 (1479.7841,1+)  :MaldiWellID: 66567, SpectrumID: 144118,
Query15 (1529.7374,1+)  :<no title>
Query16 (1563.7852,1+)  :<no title>
Query17 (1563.7852,1+)  :MaldiWellID: 66567, SpectrumID: 144112,
Query18 (1567.7542,1+)  :<no title>
Query19 (1575.8081,1+)  :<no title>
Query20 (1638.8492,1+)  :<no title>
Query21 (1759.9475,1+)  :<no title>
Query22 (1759.9475,1+)  :MaldiWellID: 66567, SpectrumID: 144109,
Query23 (1787.9674,1+)  :<no title>
Query24 (1790.9246,1+)  :<no title>
Query25 (1790.9246,1+)  :MaldiWellID: 66567, SpectrumID: 144116,
Query26 (1815.9518,1+)  :<no title>
Query27 (1858.9628,1+)  :<no title>
Query28 (1877.0386,1+)  :<no title>
Query29 (1877.0386,1+)  :MaldiWellID: 66567, SpectrumID: 144113,
Query30 (1881.0592,1+)  :<no title>
Query31 (1905.0511,1+)  :<no title>
Query32 (1906.8796,1+)  :<no title>
Query33 (1951.9500,1+)  :<no title>
Query34 (1971.0089,1+)  :<no title>
Query35 (1993.0059,1+)  :<no title>
Query36 (1993.0059,1+)  :MaldiWellID: 66567, SpectrumID: 144117,
Query37 (1994.0392,1+)  :<no title>
Query38 (1998.9767,1+)  :<no title>
Query39 (2008.9969,1+)  :<no title>
Query40 (2042.1071,1+)  :<no title>
Query41 (2074.9429,1+)  :<no title>
Query42 (2107.2236,1+)  :<no title>
Query43 (2117.0906,1+)  :<no title>
Query44 (2138.1675,1+)  :<no title>
Query45 (2139.1616,1+)  :<no title>
Query46 (2185.0957,1+)  :<no title>
Query47 (2194.0574,1+)  :<no title>
Query48 (2196.0725,1+)  :<no title>
Query49 (2202.1440,1+)  :<no title>
Query50 (2204.1467,1+)  :<no title>
Query51 (2219.1174,1+)  :<no title>
Query52 (2225.1460,1+)  :<no title>
Query53 (2228.1523,1+)  :<no title>
Query54 (2235.2080,1+)  :<no title>
Query55 (2251.1221,1+)  :<no title>
Query56 (2268.1711,1+)  :<no title>
Query57 (2282.1650,1+)  :<no title>
Query58 (2286.2263,1+)  :<no title>
Query59 (2286.2263,1+)  :MaldiWellID: 66567, SpectrumID: 144110,
Query60 (2314.1519,1+)  :<no title>
Query61 (2325.2527,1+)  :<no title>
Query62 (2327.2517,1+)  :<no title>
Query63 (2330.1921,1+)  :<no title>
Query64 (2332.1497,1+)  :<no title>
Query65 (2501.2480,1+)  :<no title>
Query66 (2566.2903,1+)  :<no title>
Query67 (2586.2400,1+)  :<no title>
Query68 (2612.1943,1+)  :<no title>
```

```
Query69 (2705.4258,1+) : <no title>
Query70 (2726.3000,1+) : <no title>
Query71 (2925.5957,1+) : <no title>
Query72 (3052.6226,1+) : <no title>
Query73 (3136.4932,1+) : <no title>
Query74 (3153.4534,1+) : <no title>
Query75 (3206.5691,1+) : <no title>
Query76 (3211.4873,1+) : <no title>
Query77 (3224.5742,1+) : <no title>
```

Mascot Search Results

Protein View

Band: 1.1

Match to: **gi|237665430** Score: **112** Expect: **1.2e-007**
rhoptry-associated protein 2 [Plasmodium falciparum]

Nominal mass (M_r): **43672**; Calculated pI value: **8.92**

NCBI BLAST search of gi|237665430 against nr
Unformatted sequence string for pasting into other applications

Taxonomy: Plasmodium falciparum

Fixed modifications: Carbamidomethyl (C)
Variable modifications: Oxidation (M)
Cleavage by Trypsin: cuts C-term side of KR unless next residue is P
Sequence Coverage: **17%**

Matched peptides shown in **Bold Red**

```
  1 SKLYPESNSL TGLIYAHTAH VHKLSMWVYF IYNHFSSADE LIKYLEKTNI
 51 NTLENSDHTC FARAVTLYLF YYYLKDIKSM LSTDDYQSFF KNKFKDINPL
101 FINDFILILN DKKFMENLDL YIMKESEREH LVIKKNPFLR VLNKASTTTH
151 ATYKSNPYFI VGSRVHTPYK DYLGDFNKYT EISVLNYVRD YNFLIYAGSR
201 ENYYNSDIAG PARSVNNVIS KNKTLGLRKR SSSLALVGTN NNDPIFAYCE
251 KDNKSEYYGT PDDLITSFFS IIKTKMLNSH KTFLRQFDYA LFHKTYSIPN
301 LKGFRFLKHL FQKKNLVNFV GMYENHVSTE INFLAEDFVE LFDVTMDCYS
351 RQYSNRAAEN FKAIRELNVL
```

Show predicted peptides also

Sort Peptides By ◉ Residue Number ○ Increasing Mass ○ Decreasing Mass

Start - End	Observed	Mr(expt)	Mr(calc)	ppm	Miss	Sequence	
1 - 23	2566.2903	2565.2830	2565.3339	-20	1	-.SKLYPESNSLTGLIYAHTAHVHK.L	(No match)
155 - 164	1139.5815	1138.5742	1138.5771	-3	0	K.SNPYFIVGSR.V	(No match)
155 - 164	1139.5815	1138.5742	1138.5771	-3	0	K.SNPYFIVGSR.V	(Ions score 38)
190 - 200	1318.6443	1317.6370	1317.6353	1	0	R.DYNFLIYAGSR.E	(No match)
190 - 200	1318.6443	1317.6370	1317.6353	1	0	R.DYNFLIYAGSR.E	(Ions score 57)
255 - 273	2196.0725	2195.0652	2195.0674	-1	0	K.SEYYGTPDDLITSFFSIIK.T	(No match)

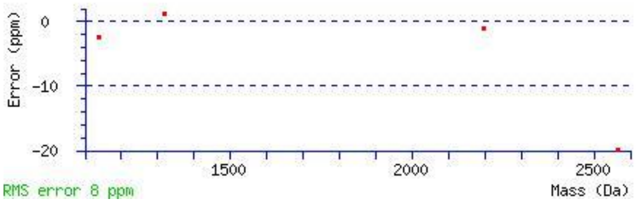

Mascot: http://www.matrixscience.com/

Mascot Search Results

**Band**	: 1.1
Email	:
Search title	: SampleSetID: 759, AnalysisID: 6312, MaldiWellID: 66567, SpectrumID: 144403, Path=\171004\MSMS\17-112
Database	: SwissProt 20170116 (553231 sequences; 197953409 residues)
Timestamp	: 30 Nov 2017 at 07:07:00 GMT
Warning	: A Peptide summary report will usually give a much clearer picture of MS/MS search results.
Top Score	: 202 for CASA1_BOVIN, Alpha-S1-casein OS=Bos taurus GN=CSN1S1 PE=1 SV=2

SP combined run 79 y 72

Mascot Score Histogram

Protein score is -10*Log(P), where P is the probability that the observed match is a random event.
Protein scores greater than 70 are significant (p<0.05).
Protein scores are derived from ions scores as a non-probabilistic basis for ranking protein hits.

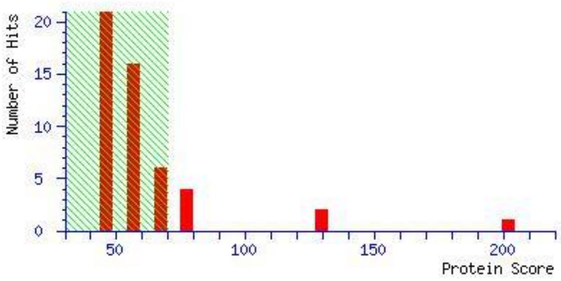

Protein Summary Report

Format As | Protein Summary (deprecated) | Help

Significance threshold p< 0.05 | Max. number of hits 20

Re-Search All | Search Unmatched

Index

Accession	Mass	Score	Description
1. CASA1_BOVIN	24570	202	Alpha-S1-casein OS=Bos taurus GN=CSN1S1 PE=1 SV=2
**2. B3AT_HUMAN**	**102013**	**130**	**Band 3 anion transport protein OS=Homo sapiens GN=SLC4A1 PE=1 SV=3**
3. CASA1_BUBBU	24368	127	Alpha-S1-casein OS=Bubalus bubalis GN=CSN1S1 PE=2 SV=2
4. HBB_HUMAN	16102	82	Hemoglobin subunit beta OS=Homo sapiens GN=HBB PE=1 SV=2
5. HBB_PANPA	16102	82	Hemoglobin subunit beta OS=Pan paniscus GN=HBB PE=1 SV=2
6. HBB_PANTR	16102	82	Hemoglobin subunit beta OS=Pan troglodytes GN=HBB PE=1 SV=2
7. HBG_TARSY	16506	76	Hemoglobin subunit gamma OS=Tarsius syrichta GN=HBG PE=2 SV=2
8. CASA1_CAPHI	24331	72	Alpha-S1-casein OS=Capra hircus GN=CSN1S1 PE=1 SV=2
9. HBB_GORGO	16074	70	Hemoglobin subunit beta OS=Gorilla gorilla gorilla GN=HBB PE=1 SV=2
10. HBG_TARBA	16388	67	Hemoglobin subunit gamma OS=Tarsius bancanus GN=HBG PE=2 SV=3
11. CLPB_MYCLE	93207	66	Chaperone protein ClpB OS=Mycobacterium leprae (strain TN) GN=clpB PE=3 SV=1
12. HBB_SCAOR	16060	65	Hemoglobin subunit beta OS=Scapanus orarius GN=HBB PE=2 SV=3
13. CASA1_SHEEP	2434764		Alpha-S1-casein OS=Ovis aries GN=CSN1S1 PE=1 SV=3
14. HBB_MARMA	15783	58	Hemoglobin subunit beta OS=Marmota marmota marmota GN=HBB PE=1 SV=1
15. HBB_SPECI	15806	58	Hemoglobin subunit beta OS=Spermophilus citellus GN=HBB PE=1 SV=1
16. HBB_UROTO	15827	58	Hemoglobin subunit beta-S/F OS=Urocitellus townsendii PE=1 SV=1
17. HBB_PROLO	16111	58	Hemoglobin subunit beta OS=Procyon lotor GN=HBB PE=1 SV=1
18. TRUB_PEPD6	34469	57	tRNA pseudouridine synthase B OS=Peptoclostridium difficile (strain 630) GN=truB PE=3 SV=1
19. HBB_CYNSP	15974	57	Hemoglobin subunit beta OS=Cynopterus sphinx GN=HBB PE=1 SV=1
20. HBB2_RAT	16086	57	Hemoglobin subunit beta-2 OS=Rattus norvegicus PE=1 SV=2

Results List

1. CASA1_BOVIN **Mass:** 24570 **Score:** 202 **Expect:** 3.5e-015 **Matches:** 8

Alpha-S1-casein OS=Bos taurus GN=CSN1S1 PE=1 SV=2

Observed	Mr(expt)	Mr(calc)	ppm	Start		End	Miss	Ions	Peptide
1267.6982	1266.6909	1266.6972	-4.95	106	-	115	0	---	R.YLGYLEQLLR.L
1267.6982	1266.6909	1266.6972	-4.95	106	-	115	0	54	R.YLGYLEQLLR.L
1384.7184	1383.7111	1383.7227	-8.39	38	-	49	0	---	R.FFVAPFPEVFGK.E
1384.7184	1383.7111	1383.7227	-8.39	38	-	49	0	56	R.FFVAPFPEVFGK.E
1759.9475	1758.9402	1758.9377	1.46	23	-	37	0	---	K.HQGLPQEVLNENLLR.F
1759.9475	1758.9402	1758.9377	1.46	23	-	37	0	62	K.HQGLPQEVLNENLLR.F
2235.2080	2234.2007	2234.2283	-12.36	19	-	37	1	---	K.HPIKHQGLPQEVLNENLLR.F
2332.1497	2331.1424	2331.1245	7.68	148	-	166	0	---	K.EPMIGVNQELAYFYPELFR.Q + Oxidation (M)

No match to: 1034.1162, 1050.0874, 1139.5815, 1139.5815, 1274.7203, 1274.7317, 1318.6443, 1318.6443, 1445.7601, 1479.7841, 1529.7374, 1563.7852, 1563.7852, 1567.7542, 1575.8081, 1638.8492, 1787.9674, 1790.9246, 1790.9246, 1815.9518, 1858.9628, 1877.0386, 1877.0386, 1881.0592, 1905.0511, 1906.8796, 1951.9500, 1971.0089, 1993.0059, 1993.0059, 1994.0392, 1998.9767, 2008.9969, 2042.1071, 2074.9429, 2107.2236, 2117.0906, 2138.1675, 2139.1616, 2185.0957, 2194.0574, 2196.0725, 2202.1440, 2204.1467, 2219.1174, 2225.1460, 2228.1523,

2251.1221, 2268.1711, 2282.1650, 2286.2263, 2286.2263, 2314.1519, 2325.2527, 2327.2517, 2330.1921, 2501.2480, 2566.2903, 2586.2400, 2612.1943, 2705.4258, 2726.3000, 2925.5957, 3052.6226, 3136.4932, 3153.4534, 3206.5691, 3211.4873, 3224.5742

2. B3AT_HUMAN **Mass:** 102013 **Score:** 130 **Expect:** 5.5e-008 **Matches:** 11

Band 3 anion transport protein OS=Homo sapiens GN=SLC4A1 PE=1 SV=3

Observed	Mr(expt)	Mr(calc)	ppm	Start	End	Miss	Ions	Peptide
1479.7841	1478.7768	1478.8609	-56.86	540	- 551	1	---	K.LIKIFQDHPLQK.T
1563.7852	1562.7779	1562.7841	-3.97	139	- 150	0	---	R.FIFEDQIRPQDR.E
1563.7852	1562.7779	1562.7841	-3.97	139	- 150	0	21	R.FIFEDQIRPQDR.E
1877.0386	1876.0313	1876.0305	0.42	247	- 263	0	---	R.LQEAAELEAVELPVPIR.F
1877.0386	1876.0313	1876.0305	0.42	247	- 263	0	50	R.LQEAAELEAVELPVPIR.F
2042.1071	2041.0998	2041.1069	-3.45	161	- 180	0	---	K.HSHAGELEALGGVKPAVLTR.S
2204.1467	2203.1394	2203.1385	0.40	139	- 155	1	---	R.FIFEDQIRPQDREELLR.A
2235.2080	2234.2007	2234.1252	33.8	57	- 74	1	---	K.VYVELQELVMDEKNQELR.W
2251.1221	2250.1148	2250.1202	-2.37	57	- 74	1	---	K.VYVELQELVMDEKNQELR.W + Oxidation (M)
2286.2263	2285.2190	2285.2208	-0.79	264	- 283	0	---	R.FLFVLLGPEAPHIDYTQLGR.A
2286.2263	2285.2190	2285.2208	-0.79	264	- 283	0	41	R.FLFVLLGPEAPHIDYTQLGR.A

No match to: 1034.1162, 1050.0874, 1139.5815, 1139.5815, 1267.6982, 1267.6982, 1274.7203, 1274.7317, 1318.6443, 1318.6443, 1384.7184, 1384.7184, 1445.7601, 1529.7374, 1567.7542, 1575.8081, 1638.8492, 1759.9475, 1759.9475, 1787.9674, 1790.9246, 1790.9246, 1815.9518, 1858.9628, 1881.0592, 1905.0511, 1906.8796, 1951.9500, 1971.0089, 1993.0059, 1993.0059, 1994.0392, 1998.9767, 2008.9969, 2074.9429, 2107.2236, 2117.0906, 2138.1675, 2139.1616, 2185.0957, 2194.0574, 2196.0725, 2202.1440, 2219.1174, 2225.1460, 2228.1523, 2268.1711, 2282.1650, 2314.1519, 2325.2527, 2327.2517, 2330.1921, 2332.1497, 2501.2480, 2566.2903, 2586.2400, 2612.1943, 2705.4258, 2726.3000, 2925.5957, 3052.6226, 3136.4932, 3153.4534, 3206.5691, 3211.4873, 3224.5742

3. CASA1_BUBBU **Mass:** 24368 **Score:** 127 **Expect:** 1.1e-007 **Matches:** 5

Alpha-S1-casein OS=Bubalus bubalis GN=CSN1S1 PE=2 SV=2

Observed	Mr(expt)	Mr(calc)	ppm	Start	End	Miss	Ions	Peptide
1267.6982	1266.6909	1266.6972	-4.95	106	- 115	0	---	R.YLGYLEQLLR.L
1267.6982	1266.6909	1266.6972	-4.95	106	- 115	0	54	R.YLGYLEQLLR.L
1384.7184	1383.7111	1383.7227	-8.39	38	- 49	0	---	R.FFVAPFPEVFGK.E
1384.7184	1383.7111	1383.7227	-8.39	38	- 49	0	56	R.FFVAPFPEVFGK.E
3206.5691	3205.5618	3205.6019	-12.49	140	- 166	1	---	K.EGIHAQQKEPMIGVNQELAYFYPQLFR.Q

No match to: 1034.1162, 1050.0874, 1139.5815, 1139.5815, 1274.7203, 1274.7317, 1318.6443, 1318.6443, 1445.7601, 1479.7841, 1529.7374, 1563.7852, 1563.7852, 1567.7542, 1575.8081, 1638.8492, 1759.9475, 1759.9475, 1787.9674, 1790.9246, 1790.9246, 1815.9518, 1858.9628, 1877.0386, 1877.0386, 1881.0592, 1905.0511, 1906.8796, 1951.9500, 1971.0089, 1993.0059, 1993.0059, 1994.0392, 1998.9767, 2008.9969, 2042.1071, 2074.9429, 2107.2236, 2117.0906, 2138.1675, 2139.1616, 2185.0957, 2194.0574, 2196.0725, 2202.1440, 2204.1467, 2219.1174, 2225.1460, 2228.1523, 2235.2080, 2251.1221, 2268.1711, 2282.1650, 2286.2263, 2286.2263, 2314.1519, 2325.2527, 2327.2517, 2330.1921, 2332.1497, 2501.2480, 2566.2903, 2586.2400, 2612.1943, 2705.4258, 2726.3000, 2925.5957, 3052.6226, 3136.4932, 3153.4534, 3211.4873, 3224.5742

4. HBB_HUMAN **Mass:** 16102 **Score:** 82 **Expect:** 0.0033 **Matches:** 8

Hemoglobin subunit beta OS=Homo sapiens GN=HBB PE=1 SV=2

Observed	Mr(expt)	Mr(calc)	ppm	Start	End	Miss	Ions	Peptide
1274.7203	1273.7130	1273.7183	-4.14	32	- 41	0	---	R.LLVVYPWTQR.F
1274.7317	1273.7244	1273.7183	4.81	32	- 41	0	25	R.LLVVYPWTQR.F
2074.9429	2073.9356	2073.9354	0.12	42	- 60	0	---	R.FFESFGDLSTPDAVMGNPK.V + Oxidation (M)
2228.1523	2227.1450	2227.1597	-6.59	10	- 31	1	---	K.SAVTALWGKVNVDEVGGEALGR.L
2286.2263	2285.2190	2285.1038	50.4	42	- 62	1	---	R.FFESFGDLSTPDAVMGNPKVK.A
2286.2263	2285.2190	2285.1038	50.4	42	- 62	1	---	R.FFESFGDLSTPDAVMGNPKVK.A
2586.2400	2585.2327	2585.2333	-0.21	84	- 105	1	---	K.GTFATLSELHCDKLHVDPENFR.L
3136.4932	3135.4859	3135.6692	-58.45	106	- 133	1	---	R.LLGNVLVCVLAHHFGKEFTPPVQAAYQK.V

No match to: 1034.1162, 1050.0874, 1139.5815, 1139.5815, 1267.6982, 1267.6982, 1318.6443, 1318.6443, 1384.7184, 1384.7184, 1445.7601, 1479.7841, 1529.7374, 1563.7852, 1563.7852, 1567.7542, 1575.8081, 1638.8492, 1759.9475, 1759.9475, 1787.9674, 1790.9246, 1790.9246, 1815.9518, 1858.9628, 1877.0386, 1877.0386, 1881.0592, 1905.0511, 1906.8796, 1951.9500, 1971.0089, 1993.0059, 1993.0059, 1994.0392, 1998.9767, 2008.9969, 2042.1071, 2107.2236, 2117.0906, 2138.1675, 2139.1616, 2185.0957, 2194.0574, 2196.0725, 2202.1440, 2204.1467, 2219.1174, 2225.1460, 2235.2080, 2251.1221, 2268.1711, 2282.1650, 2314.1519, 2325.2527, 2327.2517, 2330.1921, 2332.1497, 2501.2480, 2566.2903, 2612.1943, 2705.4258, 2726.3000, 2925.5957, 3052.6226, 3153.4534, 3206.5691, 3211.4873, 3224.5742

5. HBB_PANPA **Mass:** 16102 **Score:** 82 **Expect:** 0.0033 **Matches:** 8

Hemoglobin subunit beta OS=Pan paniscus GN=HBB PE=1 SV=2

Observed	Mr(expt)	Mr(calc)	ppm	Start	End	Miss	Ions	Peptide
1274.7203	1273.7130	1273.7183	-4.14	32	- 41	0	---	R.LLVVYPWTQR.F
1274.7317	1273.7244	1273.7183	4.81	32	- 41	0	25	R.LLVVYPWTQR.F
2074.9429	2073.9356	2073.9354	0.12	42	- 60	0	---	R.FFESFGDLSTPDAVMGNPK.V + Oxidation (M)
2228.1523	2227.1450	2227.1597	-6.59	10	- 31	1	---	K.SAVTALWGKVNVDEVGGEALGR.L
2286.2263	2285.2190	2285.1038	50.4	42	- 62	1	---	R.FFESFGDLSTPDAVMGNPKVK.A
2286.2263	2285.2190	2285.1038	50.4	42	- 62	1	---	R.FFESFGDLSTPDAVMGNPKVK.A
2586.2400	2585.2327	2585.2333	-0.21	84	- 105	1	---	K.GTFATLSELHCDKLHVDPENFR.L
3136.4932	3135.4859	3135.6692	-58.45	106	- 133	1	---	R.LLGNVLVCVLAHHFGKEFTPPVQAAYQK.V

No match to: 1034.1162, 1050.0874, 1139.5815, 1139.5815, 1267.6982, 1267.6982, 1318.6443, 1318.6443, 1384.7184, 1384.7184, 1445.7601, 1479.7841, 1529.7374, 1563.7852, 1563.7852, 1567.7542, 1575.8081, 1638.8492, 1759.9475, 1759.9475, 1787.9674, 1790.9246, 1790.9246, 1815.9518, 1858.9628, 1877.0386, 1877.0386, 1881.0592, 1905.0511, 1906.8796, 1951.9500, 1971.0089, 1993.0059, 1993.0059, 1994.0392, 1998.9767, 2008.9969, 2042.1071, 2107.2236, 2117.0906, 2138.1675, 2139.1616, 2185.0957, 2194.0574, 2196.0725, 2202.1440, 2204.1467, 2219.1174, 2225.1460, 2235.2080, 2251.1221, 2268.1711, 2282.1650, 2314.1519, 2325.2527, 2327.2517, 2330.1921, 2332.1497, 2501.2480, 2566.2903, 2612.1943, 2705.4258, 2726.3000, 2925.5957, 3052.6226, 3153.4534, 3206.5691, 3211.4873, 3224.5742

6. HBB_PANTR **Mass:** 16102 **Score:** 82 **Expect:** 0.0033 **Matches:** 8

Hemoglobin subunit beta OS=Pan troglodytes GN=HBB PE=1 SV=2

Observed	Mr(expt)	Mr(calc)	ppm	Start	End	Miss	Ions	Peptide
1274.7203	1273.7130	1273.7183	-4.14	32	- 41	0	---	R.LLVVYPWTQR.F

1274.7317	1273.7244	1273.7183	4.81	32	-	41	0	25	R.LLVVYPWTQR.F
2074.9429	2073.9356	2073.9354	0.12	42	-	60	0	---	R.FFESFGDLSTPDAVMGNPK.V + Oxidation (M)
2228.1523	2227.1450	2227.1597	-6.59	10	-	31	1	---	K.SAVTALWGKVNVDEVGGEALGR.L
2286.2263	2285.2190	2285.1038	50.4	42	-	62	1	---	R.FFESFGDLSTPDAVMGNPKVK.A
2286.2263	2285.2190	2285.1038	50.4	42	-	62	1	---	R.FFESFGDLSTPDAVMGNPKVK.A
2586.2400	2585.2327	2585.2333	-0.21	84	-	105	1	---	K.GTFATLSELHCDKLHVDPENFR.L
3136.4932	3135.4859	3135.6692	-58.45	106	-	133	1	---	R.LLGNVLVCVLAHHFGKEFTPPVQAAYQK.V

No match to: 1034.1162, 1050.0874, 1139.5815, 1139.5815, 1267.6982, 1267.6982, 1318.6443, 1318.6443, 1384.7184, 1384.7184, 1445.7601, 1479.7841, 1529.7374, 1563.7852, 1563.7852, 1567.7542, 1575.8081, 1638.8492, 1759.9475, 1759.9475, 1787.9674, 1790.9246, 1790.9246, 1815.9518, 1858.9628, 1877.0386, 1877.0386, 1881.0592, 1905.0511, 1906.8796, 1951.9500, 1971.0089, 1993.0059, 1993.0059, 1994.0392, 1998.9767, 2008.9969, 2042.1071, 2107.2236, 2117.0906, 2138.1675, 2139.1616, 2185.0957, 2194.0574, 2196.0725, 2202.1440, 2204.1467, 2219.1174, 2225.1460, 2235.2080, 2251.1221, 2268.1711, 2282.1650, 2314.1519, 2325.2527, 2327.2517, 2330.1921, 2332.1497, 2501.2480, 2566.2903, 2612.1943, 2705.4258, 2726.3000, 2925.5957, 3052.6226, 3153.4534, 3206.5691, 3211.4873, 3224.5742

7. HBG_TARSY **Mass:** 16506 **Score:** 76 **Expect:** 0.012 **Matches:** 7

Hemoglobin subunit gamma OS=Tarsius syrichta GN=HBG PE=2 SV=2

Observed	Mr(expt)	Mr(calc)	ppm	Start		End	Miss	Ions	Peptide
1274.7203	1273.7130	1273.7183	-4.14	32	-	41	0	---	R.LLVVYPWTQR.F
1274.7317	1273.7244	1273.7183	4.81	32	-	41	0	25	R.LLVVYPWTQR.F
1858.9628	1857.9555	1857.9506	2.65	67	-	83	1	---	K.KVLSSLGEAVTHMDDLK.D + Oxidation (M)
2074.9429	2073.9356	2074.0921	-75.44	1	-	18	1	---	-.MVHFTAEEKAIITSLWAK.V
2314.1519	2313.1446	2313.2328	-38.13	10	-	31	1	---	K.AIITSLWAKVNVEETGGEALGR.L
2586.2400	2585.2327	2585.3602	-49.30	19	-	41	1	---	K.VNVEETGGEALGRLLVVYPWTQR.F
2612.1943	2611.1870	2611.3064	-45.73	68	-	91	1	---	K.VLSSLGEAVTHMDDLKDAFAHLSR.L

No match to: 1034.1162, 1050.0874, 1139.5815, 1139.5815, 1267.6982, 1267.6982, 1318.6443, 1318.6443, 1384.7184, 1384.7184, 1445.7601, 1479.7841, 1529.7374, 1563.7852, 1563.7852, 1567.7542, 1575.8081, 1638.8492, 1759.9475, 1759.9475, 1787.9674, 1790.9246, 1790.9246, 1815.9518, 1877.0386, 1877.0386, 1881.0592, 1905.0511, 1906.8796, 1951.9500, 1971.0089, 1993.0059, 1993.0059, 1994.0392, 1998.9767, 2008.9969, 2042.1071, 2107.2236, 2117.0906, 2138.1675, 2139.1616, 2185.0957, 2194.0574, 2196.0725, 2202.1440, 2204.1467, 2219.1174, 2225.1460, 2228.1523, 2235.2080, 2251.1221, 2268.1711, 2282.1650, 2286.2263, 2286.2263, 2325.2527, 2327.2517, 2330.1921, 2332.1497, 2501.2480, 2566.2903, 2705.4258, 2726.3000, 2925.5957, 3052.6226, 3136.4932, 3153.4534, 3206.5691, 3211.4873, 3224.5742

8. CASA1_CAPHI **Mass:** 24331 **Score:** 72 **Expect:** 0.037 **Matches:** 4

Alpha-S1-casein OS=Capra hircus GN=CSN1S1 PE=1 SV=2

Observed	Mr(expt)	Mr(calc)	ppm	Start		End	Miss	Ions	Peptide
1267.6982	1266.6909	1266.6972	-4.95	106	-	115	0	---	R.YLGYLEQLLR.L
1267.6982	1266.6909	1266.6972	-4.95	106	-	115	0	54	R.YLGYLEQLLR.L
2194.0574	2193.0501	2193.0219	12.9	74	-	94	1	---	K.QMKAGSSSSSEEIVPNSAEQK.Y
2726.3000	2725.2927	2725.4592	-61.07	25	-	48	1	---	R.GLSPEVPNENLLRFVVAPFPEVFR.K

No match to: 1034.1162, 1050.0874, 1139.5815, 1139.5815, 1274.7203, 1274.7317, 1318.6443, 1318.6443, 1384.7184, 1384.7184, 1445.7601, 1479.7841, 1529.7374, 1563.7852, 1563.7852, 1567.7542, 1575.8081, 1638.8492, 1759.9475, 1759.9475, 1787.9674, 1790.9246, 1790.9246, 1815.9518, 1858.9628, 1877.0386, 1877.0386, 1881.0592, 1905.0511, 1906.8796, 1951.9500, 1971.0089, 1993.0059, 1993.0059, 1994.0392, 1998.9767, 2008.9969, 2042.1071, 2074.9429, 2107.2236, 2117.0906, 2138.1675, 2139.1616, 2185.0957, 2196.0725, 2202.1440, 2204.1467, 2219.1174, 2225.1460, 2228.1523, 2235.2080, 2251.1221, 2268.1711, 2282.1650, 2286.2263, 2286.2263, 2314.1519, 2325.2527, 2327.2517, 2330.1921, 2332.1497, 2501.2480, 2566.2903, 2586.2400, 2612.1943, 2705.4258, 2925.5957, 3052.6226, 3136.4932, 3153.4534, 3206.5691, 3211.4873, 3224.5742

9. HBB_GORGO **Mass:** 16074 **Score:** 70 **Expect:** 0.062 **Matches:** 7

Hemoglobin subunit beta OS=Gorilla gorilla gorilla GN=HBB PE=1 SV=2

Observed	Mr(expt)	Mr(calc)	ppm	Start		End	Miss	Ions	Peptide
1274.7203	1273.7130	1273.7183	-4.14	32	-	41	0	---	R.LLVVYPWTQR.F
1274.7317	1273.7244	1273.7183	4.81	32	-	41	0	25	R.LLVVYPWTQR.F
2074.9429	2073.9356	2073.9354	0.12	42	-	60	0	---	R.FFESFGDLSTPDAVMGNPK.V + Oxidation (M)
2228.1523	2227.1450	2227.1597	-6.59	10	-	31	1	---	K.SAVTALWGKVNVDEVGGEALGR.L
2286.2263	2285.2190	2285.1038	50.4	42	-	62	1	---	R.FFESFGDLSTPDAVMGNPKVK.A
2286.2263	2285.2190	2285.1038	50.4	42	-	62	1	---	R.FFESFGDLSTPDAVMGNPKVK.A
3136.4932	3135.4859	3135.6692	-58.45	106	-	133	1	---	K.LLGNVLVCVLAHHFGKEFTPPVQAAYQK.V

No match to: 1034.1162, 1050.0874, 1139.5815, 1139.5815, 1267.6982, 1267.6982, 1318.6443, 1318.6443, 1384.7184, 1384.7184, 1445.7601, 1479.7841, 1529.7374, 1563.7852, 1563.7852, 1567.7542, 1575.8081, 1638.8492, 1759.9475, 1759.9475, 1787.9674, 1790.9246, 1790.9246, 1815.9518, 1858.9628, 1877.0386, 1877.0386, 1881.0592, 1905.0511, 1906.8796, 1951.9500, 1971.0089, 1993.0059, 1993.0059, 1994.0392, 1998.9767, 2008.9969, 2042.1071, 2107.2236, 2117.0906, 2138.1675, 2139.1616, 2185.0957, 2194.0574, 2196.0725, 2202.1440, 2204.1467, 2219.1174, 2225.1460, 2235.2080, 2251.1221, 2268.1711, 2282.1650, 2314.1519, 2325.2527, 2327.2517, 2330.1921, 2332.1497, 2501.2480, 2566.2903, 2586.2400, 2612.1943, 2705.4258, 2726.3000, 2925.5957, 3052.6226, 3153.4534, 3206.5691, 3211.4873, 3224.5742

Search Parameters

```
Type of search          :Sequence Query
Enzyme                  :Trypsin
Fixed modifications     :Carbamidomethyl (C)
Variable modifications : Oxidation (M)
Mass values             :Monoisotopic
Protein Mass            :Unrestricted
Peptide Mass Tolerance : ± 80 ppm
Fragment Mass Tolerance: ± 0.3 Da
Max Missed Cleavages    :1
Instrument type         :MALDI-TOF-TOF
Query1 (1034.1162,1+)   :<no title>
Query2 (1050.0874,1+)   :<no title>
Query3 (1139.5815,1+)   :<no title>
Query4 (1139.5815,1+)   :MaldiWellID: 66567, SpectrumID: 144412,
Query5 (1267.6982,1+)   :<no title>
Query6 (1267.6982,1+)   :MaldiWellID: 66567, SpectrumID: 144406,
Query7 (1274.7203,1+)   :<no title>
Query8 (1274.7317,1+)   :MaldiWellID: 66567, SpectrumID: 144409,
Query9 (1318.6443,1+)   :<no title>
 Query10 (1318.6443,1+) :MaldiWellID: 66567, SpectrumID: 144413,
 Query11 (1384.7184,1+) :<no title>
 Query12 (1384.7184,1+) :MaldiWellID: 66567, SpectrumID: 144405,
 Query13 (1445.7601,1+) :<no title>
 Query14 (1479.7841,1+) :MaldiWellID: 66567, SpectrumID: 144416,
 Query15 (1529.7374,1+) :<no title>
 Query16 (1563.7852,1+) :<no title>
 Query17 (1563.7852,1+) :MaldiWellID: 66567, SpectrumID: 144410,
 Query18 (1567.7542,1+) :<no title>
 Query19 (1575.8081,1+) :<no title>
 Query20 (1638.8492,1+) :<no title>
 Query21 (1759.9475,1+) :<no title>
 Query22 (1759.9475,1+) :MaldiWellID: 66567, SpectrumID: 144407,
 Query23 (1787.9674,1+) :<no title>
 Query24 (1790.9246,1+) :<no title>
 Query25 (1790.9246,1+) :MaldiWellID: 66567, SpectrumID: 144414,
 Query26 (1815.9518,1+) :<no title>
 Query27 (1858.9628,1+) :<no title>
 Query28 (1877.0386,1+) :<no title>
 Query29 (1877.0386,1+) :MaldiWellID: 66567, SpectrumID: 144411,
 Query30 (1881.0592,1+) :<no title>
 Query31 (1905.0511,1+) :<no title>
 Query32 (1906.8796,1+) :<no title>
 Query33 (1951.9500,1+) :<no title>
 Query34 (1971.0089,1+) :<no title>
 Query35 (1993.0059,1+) :<no title>
 Query36 (1993.0059,1+) :MaldiWellID: 66567, SpectrumID: 144415,
 Query37 (1994.0392,1+) :<no title>
 Query38 (1998.9767,1+) :<no title>
 Query39 (2008.9969,1+) :<no title>
 Query40 (2042.1071,1+) :<no title>
 Query41 (2074.9429,1+) :<no title>
 Query42 (2107.2236,1+) :<no title>
 Query43 (2117.0906,1+) :<no title>
 Query44 (2138.1675,1+) :<no title>
 Query45 (2139.1616,1+) :<no title>
 Query46 (2185.0957,1+) :<no title>
 Query47 (2194.0574,1+) :<no title>
 Query48 (2196.0725,1+) :<no title>
 Query49 (2202.1440,1+) :<no title>
 Query50 (2204.1467,1+) :<no title>
 Query51 (2219.1174,1+) :<no title>
 Query52 (2225.1460,1+) :<no title>
 Query53 (2228.1523,1+) :<no title>
 Query54 (2235.2080,1+) :<no title>
 Query55 (2251.1221,1+) :<no title>
 Query56 (2268.1711,1+) :<no title>
 Query57 (2282.1650,1+) :<no title>
 Query58 (2286.2263,1+) :<no title>
 Query59 (2286.2263,1+) :MaldiWellID: 66567, SpectrumID: 144408,
 Query60 (2314.1519,1+) :<no title>
 Query61 (2325.2527,1+) :<no title>
 Query62 (2327.2517,1+) :<no title>
 Query63 (2330.1921,1+) :<no title>
 Query64 (2332.1497,1+) :<no title>
 Query65 (2501.2480,1+) :<no title>
 Query66 (2566.2903,1+) :<no title>
```

```
Query67 (2586.2400,1+) : <no title>
Query68 (2612.1943,1+) : <no title>
Query69 (2705.4258,1+) : <no title>
Query70 (2726.3000,1+) : <no title>
Query71 (2925.5957,1+) : <no title>
Query72 (3052.6226,1+) : <no title>
Query73 (3136.4932,1+) : <no title>
Query74 (3153.4534,1+) : <no title>
Query75 (3206.5691,1+) : <no title>
Query76 (3211.4873,1+) : <no title>
Query77 (3224.5742,1+) : <no title>
```

Mascot Search Results

Protein View

Band: 1.1

Match to: **B3AT_HUMAN** Score: **130** Expect: **5.5e-008**
Band 3 anion transport protein OS=Homo sapiens GN=SLC4A1 PE=1 SV=3

Nominal mass (Mr): **102013**; Calculated pI value: **5.08**

NCBI BLAST search of B3AT_HUMAN against nr
Unformatted sequence string for pasting into other applications

Taxonomy: Homo sapiens

Fixed modifications: Carbamidomethyl (C)
Variable modifications: Oxidation (M)
Cleavage by Trypsin: cuts C-term side of KR unless next residue is P
Sequence Coverage: **11%**

Matched peptides shown in **Bold Red**

```
1 MEELQDDYED MMEENLEQEE YEDPDIPESQ MEEPAAHDTE ATATDYHTTS
51 HPGTHKVYVE LQELVMDEKN QELRWMEAAR WVQLEENLGE NGAWGRPHLS
101 HLTFWSLLEL RRVFTKGTVL LDLQETSLAG VANQLLDRFI FEDQIRPQDR
151 EELLRALLLK HSHAGELEAL GGVKPAVLTR SGDPSQPLLP QHSSLETQLF
201 CEQGDGGTEG HSPSGILEKI PPDSEATLVL VGRADFLEQP VLGFVRLQEA
251 AELEAVELPV PIRFLFVLLG PEAPHIDYTQ LGRAAATLMS ERVFRIDAYM
301 AQSRGELLHS LEGFLDCSLV LPPTDAPSEQ ALLSLVPVQR ELLRRRYQSS
351 PAKPDSSFYK GLDLNGGPDD PLQQTGQLFG GLVRDIRRRY PYYLSDITDA
401 FSPQVLAAVI FIYFAALSPA ITFGGLLGEK TRNQMGVSEL LISTAVQGIL
451 FALLGAQPLL VVGFSGPLLV FEEAFFSFCE TNGLEYIVGR VWIGFWLILL
501 VVLVVAFEGS FLVRFISRYT QEIFSFLISL IFIYETFSKL IKIFQDHPLQ
551 KTYNYNVLMV PKPQGPLPNT ALLSLVLMAG TFFFAMMLRK FKNSSYFPGK
601 LRRVIGDFGV PISILIMVLV DFFIQDTYTQ KLSVPDGFKV SNSSARGWVI
651 HPLGLRSEFP IWMMFASALP ALLVFILIFL ESQITTLIVS KPERKMVKGS
701 GFHLDLLLVV GMGGVAALFG MPWLSATTVR SVTHANALTV MGKASTPGAA
751 AQIQEVKEQR ISGLLVAVLV GLSILMEPIL SRIPLAVLFG IFLYMGVTSL
801 SGIQLFDRIL LLFKPPKYHP DVPYVKRVKT WRMHLFTGIQ IICLAVLWVV
851 KSTPASLALP FVLILTVPLR RVLLPLIFRN VELQCLDADD AKATFDEEEG
901 RDEYDEVAMP V
```

Show predicted peptides also

Sort Peptides By ◉ Residue Number ○ Increasing Mass ○ Decreasing Mass

Start - End	Observed	Mr(expt)	Mr(calc)	ppm	Miss	Sequence	
57 - 74	2235.2080	2234.2007	2234.1252	34	1	K.VYVELQELVMDEKNQELR.W	(No match)
57 - 74	2251.1221	2250.1148	2250.1202	-2	1	K.VYVELQELVMDEKNQELR.W	Oxidation (M) (No match)
139 - 150	1563.7852	1562.7779	1562.7841	-4	0	R.FIFEDQIRPQDR.E	(No match)
139 - 150	1563.7852	1562.7779	1562.7841	-4	0	R.FIFEDQIRPQDR.E	(Ions score 21)
139 - 155	2204.1467	2203.1394	2203.1385	0	1	R.FIFEDQIRPQDREELLR.A	(No match)
161 - 180	2042.1071	2041.0998	2041.1069	-3	0	K.HSHAGELEALGGVKPAVLTR.S	(No match)
247 - 263	1877.0386	1876.0313	1876.0305	0	0	R.LQEAAELEAVELPVPIR.F	(No match)
247 - 263	1877.0386	1876.0313	1876.0305	0	0	R.LQEAAELEAVELPVPIR.F	(Ions score 50)
264 - 283	2286.2263	2285.2190	2285.2208	-1	0	R.FLFVLLGPEAPHIDYTQLGR.A	(No match)
264 - 283	2286.2263	2285.2190	2285.2208	-1	0	R.FLFVLLGPEAPHIDYTQLGR.A	(Ions score 41)
540 - 551	1479.7841	1478.7768	1478.8609	-57	1	K.LIKIFQDHPLQK.T	(No match)

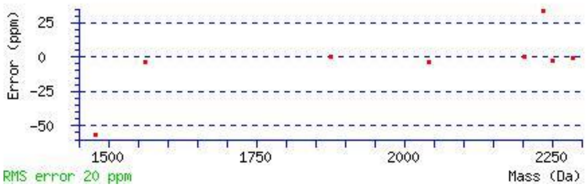

Mascot: http://www.matrixscience.com/

Mascot Search Results

Protein View

Band: 1.1

Match to: **HBB_HUMAN** Score: **82** Expect: **0.0033**
Hemoglobin subunit beta OS=Homo sapiens GN=HBB PE=1 SV=2

Nominal mass (Mr): **16102**; Calculated pI value: **6.75**

NCBI BLAST search of HBB_HUMAN against nr
Unformatted sequence string for pasting into other applications

Taxonomy: Homo sapiens

Fixed modifications: Carbamidomethyl (C)
Variable modifications: Oxidation (M)
Cleavage by Trypsin: cuts C-term side of KR unless next residue is P
Sequence Coverage: **70%**

Matched peptides shown in **Bold Red**

1 MVHLTPEEK**S AVTALWGKVN VDEVGGEALG RLLVVYPWTQ RFFESFGDLS**
51 TPDAVMGNPK VKAHGKKVLG AFSDGLAHLD NLK**GTFATLS ELHCDKLHVD**
101 PENFRLLGNV LVCVLAHHFG KEFTPPVQAA YQKVVAGVAN ALAHKYH

Show predicted peptides also

Sort Peptides By ◉ Residue Number Increasing Mass ○ Decreasing Mass

Start - End	Observed	Mr(expt)	Mr(calc)	ppm	Miss	Sequence	
10 - 31	2228.1523	2227.1450	2227.1597	-7	1	K.SAVTALWGKVNVDEVGGEALGR.L	(No match)
32 - 41	1274.7203	1273.7130	1273.7183	-4	0	R.LLVVYPWTQR.F	(No match)
32 - 41	1274.7317	1273.7244	1273.7183	5	0	R.LLVVYPWTQR.F	(Ions score 25)
42 - 60	2074.9429	2073.9356	2073.9354	0	0	R.FFESFGDLSTPDAVMGNPK.V Oxidation (M)	(No match)
42 - 62	2286.2263	2285.2190	2285.1038	50	1	R.FFESFGDLSTPDAVMGNPKVK.A	(No match)
42 - 62	2286.2263	2285.2190	2285.1038	50	1	R.FFESFGDLSTPDAVMGNPKVK.A	(No match)
84 - 105	2586.2400	2585.2327	2585.2333	-0	1	K.GTFATLSELHCDKLHVDPENFR.L	(No match)
106 - 133	3136.4932	3135.4859	3135.6692	-58	1	R.LLGNVLVCVLAHHFGKEFTPPVQAAYQK.V	(No match)

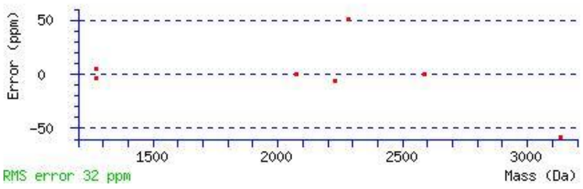

Mascot: http://www.matrixscience.com/

Mascot Search Results

Band : 1.2
Search title : SampleSetID: 759, AnalysisID: 6312, MaldiWellID: 66568, SpectrumID: 144417, Path=\171004\MSMS\17-112
Database : SwissProt 20170116 (553231 sequences; 197953409 residues)
Timestamp : 30 Nov 2017 at 07:07:19 GMT
Warning : A Peptide summary report will usually give a much clearer picture of MS/MS search results.
Top Score : 274 for B3AT_HUMAN, Band 3 anion transport protein OS=Homo sapiens GN=SLC4A1 PE=1 SV=3

SP combined run 79 y 72

Mascot Score Histogram

Protein score is -10*Log(P), where P is the probability that the observed match is a random event.
Protein scores greater than 70 are significant (p<0.05).
Protein scores are derived from ions scores as a non-probabilistic basis for ranking protein hits.

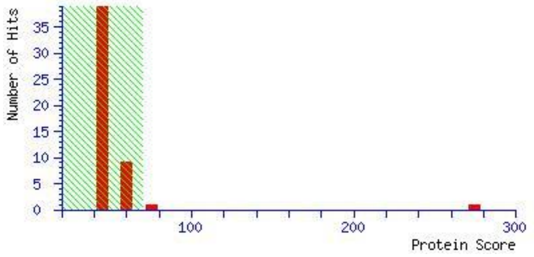

Protein Summary Report

Format As | Protein Summary (deprecated) | Help

Significance threshold p< 0.05 Max. number of hits 20

Re-Search All | Search Unmatched

Index

	Accession	Mass	Score	Description
1.	B3AT_HUMAN	102013	274	Band 3 anion transport protein OS=Homo sapiens GN=SLC4A1 PE=1 SV=3
2.	CASA1_BOVIN	2457080		Alpha-S1-casein OS=Bos taurus GN=CSN1S1 PE=1 SV=2
3.	CASA1_BUBBU	2436865		Alpha-S1-casein OS=Bubalus bubalis GN=CSN1S1 PE=2 SV=2
4.	YNI2_BACSX	29146	64	Uncharacterized protein in nit 5'region OS=Bacillus sp. (strain OxB-1) PE=4 SV=1
5.	MDV1_CANGA	80505	63	Mitochondrial division protein 1 OS=Candida glabrata (strain ATCC 2001 / CBS 138 / JCM 3761 / NBRC 0622 / NRRL Y-65) GN=MD
6.	END4_MYCPN	32212	58	Probable endonuclease 4 OS=Mycoplasma pneumoniae (strain ATCC 29342 / M129) GN=nfo PE=3 SV=1
7.	PPR1_ARATH	46732	58	Pentatricopeptide repeat-containing protein At1g01970 OS=Arabidopsis thaliana GN=At1g01970 PE=2 SV=1
8.	HIS6_TERTT	27613	56	Imidazole glycerol phosphate synthase subunit HisF OS=Teredinibacter turnerae (strain ATCC 39867 / T7901) GN=hisF PE=3 SV=
9.	PURO_METM5	22766	56	IMP cyclohydrolase OS=Methanococcus maripaludis (strain C5 / ATCC BAA-1333) GN=purO PE=3 SV=1
10.	SUCC_BART1	43131	55	Succinate--CoA ligase [ADP-forming] subunit beta OS=Bartonella tribocorum (strain CIP 105476 / IBS 506) GN=sucC PE=3 SV=1
11.	ATPB2_RHOFT	5644455		ATP synthase subunit beta 2 OS=Rhodoferax ferrireducens (strain ATCC BAA-621 / DSM 15236 / T118) GN=atpD2 PE=3 SV=1
12.	CPT1A_MOUSE	8893652		Carnitine O-palmitoyltransferase 1, liver isoform OS=Mus musculus GN=Cpt1a PE=1 SV=4
13.	MUTS_FRATN	95981	52	DNA mismatch repair protein MutS OS=Francisella tularensis subsp. novicida (strain U112) GN=mutS PE=3 SV=1
14.	XB31_ARATH	50775	52	Putative E3 ubiquitin-protein ligase XBAT31 OS=Arabidopsis thaliana GN=XBAT31 PE=2 SV=1
15.	SYR_KLEP7	64430	51	Arginine--tRNA ligase OS=Klebsiella pneumoniae subsp. pneumoniae (strain ATCC 700721 / MGH 78578) GN=argS PE=1 SV=1
16.	TRUB_PEPD6	34469	50	tRNA pseudouridine synthase B OS=Peptoclostridium difficile (strain 630) GN=truB PE=3 SV=1
17.	TOB1B_XENLA	17106650		DNA topoisomerase 2-binding protein 1-B OS=Xenopus laevis GN=topbp1-B PE=2 SV=1
18.	TRUB_GEOTN	33418	50	tRNA pseudouridine synthase B OS=Geobacillus thermodenitrificans (strain NG80-2) GN=truB PE=3 SV=1
19.	KPYR_RAT	62504	50	Pyruvate kinase PKLR OS=Rattus norvegicus GN=Pklr PE=1 SV=2
20.	BXL2_ARATH	83845	50	Probable beta-D-xylosidase 2 OS=Arabidopsis thaliana GN=BXL2 PE=2 SV=1

Results List

1. B3AT_HUMAN **Mass:** 102013 **Score:** 274 **Expect:** 2.2e-022 **Matches:** 13

Band 3 anion transport protein OS=Homo sapiens GN=SLC4A1 PE=1 SV=3

Observed	Mr(expt)	Mr(calc)	ppm	Start		End	Miss	Ions	Peptide
1466.8042	1465.7969	1465.8140	-11.68	220	-	233	0	---	K.IPPDSEATLVLVGR.A
1563.7766	1562.7693	1562.7841	-9.48	139	-	150	0	---	R.FIFEDQIRPQDR.E
1563.7766	1562.7693	1562.7841	-9.48	139	-	150	0	41	R.FIFEDQIRPQDR.E
1877.0319	1876.0246	1876.0305	-3.15	247	-	263	0	---	R.LQEAAELEAVELPVPIR.F
1877.0319	1876.0246	1876.0305	-3.15	247	-	263	0	121	R.LQEAAELEAVELPVPIR.F
2042.1002	2041.0929	2041.1069	-6.83	161	-	180	0	---	K.HSHAGELEALGGVKPAVLTR.S
2042.1002	2041.0929	2041.1069	-6.83	161	-	180	0	78	K.HSHAGELEALGGVKPAVLTR.S
2204.1377	2203.1304	2203.1385	-3.68	139	-	155	1	---	R.FIFEDQIRPQDREELLR.A
2235.1809	2234.1736	2234.1252	21.7	57	-	74	1	---	K.VYVELQELVMDEKNQELR.W
2251.1287	2250.1214	2250.1202	0.56	57	-	74	1	---	K.VYVELQELVMDEKNQELR.W + Oxidation (M)
2257.1682	2256.1609	2256.2527	-40.67	833	-	851	0	---	R.MHLFTGIQIICLAVLWVVK.S + Oxidation (M)
2286.2207	2285.2134	2285.2208	-3.25	264	-	283	0	---	R.FLFVLLGPEAPHIDYTQLGR.A
2326.2573	2325.2500	2325.2540	-1.70	117	-	138	0	---	K.GTVLLDLQETSLAGVANQLLDR.F

No match to: 1034.1259, 1050.1039, 1267.6926, 1274.7139, 1383.7844, 1384.7263, 1384.7263, 1442.7651, 1445.7534, 1475.7468, 1518.8203, 1529.7251, 1575.7903, 1638.8424, 1648.8182, 1716.8835, 1759.9320, 1762.9080, 1786.8893, 1790.9202, 1831.9440, 1859.0093, 1881.0465, 1905.0568, 1950.9700, 1970.9786, 1994.0464, 2070.0525, 2074.9507, 2107.2280, 2107.2280, 2117.0906, 2178.1001, 2186.1506, 2194.0940, 2225.1450, 2228.1609, 2250.1626, 2263.1658, 2268.2048, 2282.1624, 2289.2249, 2292.1792, 2308.1628, 2313.1716, 2314.1470, 2325.2048, 2332.1472, 2367.2200, 2468.2456, 2526.2200, 2549.3303, 2611.2832, 3052.6353, 3135.5813, 3153.5376, 3211.5759

2. CASA1_BOVIN **Mass:** 24570 **Score:** 80 **Expect:** 0.0059 **Matches:** 6

Alpha-S1-casein OS=Bos taurus GN=CSN1S1 PE=1 SV=2

Observed	Mr(expt)	Mr(calc)	ppm	Start		End	Miss	Ions	Peptide
1267.6926	1266.6853	1266.6972	-9.37	106	-	115	0	---	R.YLGYLEQLLR.L
1384.7263	1383.7190	1383.7227	-2.68	38	-	49	0	---	R.FFVAPFPEVFGK.E
1384.7263	1383.7190	1383.7227	-2.68	38	-	49	0	49	R.FFVAPFPEVFGK.E
1759.9320	1758.9247	1758.9377	-7.35	23	-	37	0	---	K.HQGLPQEVLNENLLR.F
2235.1809	2234.1736	2234.2283	-24.49	19	-	37	1	---	K.HPIKHQGLPQEVLNENLLR.F
2332.1472	2331.1399	2331.1245	6.60	148	-	166	0	---	K.EPMIGVNQELAYFYPELFR.Q + Oxidation (M)

No match to: 1034.1259, 1050.1039, 1274.7139, 1383.7844, 1442.7651, 1445.7534, 1466.8042, 1475.7468, 1518.8203, 1529.7251, 1563.7766, 1563.7766, 1575.7903, 1638.8424, 1648.8182, 1716.8835, 1762.9080, 1786.8893, 1790.9202, 1831.9440, 1859.0093, 1877.0319, 1877.0319, 1881.0465, 1905.0568, 1950.9700, 1970.9786, 1994.0464, 2042.1002, 2042.1002, 2070.0525, 2074.9507, 2107.2280, 2107.2280, 2117.0906, 2178.1001, 2186.1506, 2194.0940, 2204.1377, 2225.1450, 2228.1609, 2250.1626, 2251.1287, 2257.1682, 2263.1658, 2268.2048, 2282.1624, 2286.2207, 2289.2249, 2292.1792, 2308.1628, 2313.1716, 2314.1470, 2325.2048, 2326.2573, 2367.2200, 2468.2456, 2526.2200, 2549.3303, 2611.2832, 3052.6353, 3135.5813, 3153.5376, 3211.5759

Search Parameters

Type of search	:Sequence Query
Enzyme	:Trypsin
Fixed modifications	:Carbamidomethyl (C)
Variable modifications	: Oxidation (M)
Mass values	:Monoisotopic
Protein Mass	:Unrestricted
Peptide Mass Tolerance	: ± 80 ppm
Fragment Mass Tolerance	: ± 0.3 Da
Max Missed Cleavages	:1
Instrument type	:MALDI-TOF-TOF
Query1 (1034.1259,1+)	:<no title>
Query2 (1050.1039,1+)	:<no title>
Query3 (1267.6926,1+)	:<no title>
Query4 (1274.7139,1+)	:<no title>
Query5 (1383.7844,1+)	:<no title>
Query6 (1384.7263,1+)	:<no title>
Query7 (1384.7263,1+)	:MaldiWellID: 66568, SpectrumID: 144422,
Query8 (1442.7651,1+)	:<no title>
Query9 (1445.7534,1+)	:<no title>
Query10 (1466.8042,1+)	:<no title>
Query11 (1475.7468,1+)	:<no title>
Query12 (1518.8203,1+)	:<no title>
Query13 (1529.7251,1+)	:<no title>
Query14 (1563.7766,1+)	:<no title>
Query15 (1563.7766,1+)	:MaldiWellID: 66568, SpectrumID: 144421,
Query16 (1575.7903,1+)	:<no title>
Query17 (1638.8424,1+)	:<no title>
Query18 (1648.8182,1+)	:<no title>
Query19 (1716.8835,1+)	:<no title>
Query20 (1759.9320,1+)	:<no title>
Query21 (1762.9080,1+)	:<no title>
Query22 (1786.8893,1+)	:<no title>
Query23 (1790.9202,1+)	:<no title>
Query24 (1831.9440,1+)	:<no title>
Query25 (1859.0093,1+)	:<no title>
Query26 (1877.0319,1+)	:<no title>
Query27 (1877.0319,1+)	:MaldiWellID: 66568, SpectrumID: 144420,
Query28 (1881.0465,1+)	:<no title>
Query29 (1905.0568,1+)	:<no title>
Query30 (1950.9700,1+)	:<no title>
Query31 (1970.9786,1+)	:<no title>
Query32 (1994.0464,1+)	:<no title>
Query33 (2042.1002,1+)	:<no title>
Query34 (2042.1002,1+)	:MaldiWellID: 66568, SpectrumID: 144419,
Query35 (2070.0525,1+)	:<no title>
Query36 (2074.9507,1+)	:<no title>
Query37 (2107.2280,1+)	:<no title>
Query38 (2107.2280,1+)	:MaldiWellID: 66568, SpectrumID: 144418,
Query39 (2117.0906,1+)	:<no title>
Query40 (2178.1001,1+)	:<no title>
Query41 (2186.1506,1+)	:<no title>
Query42 (2194.0940,1+)	:<no title>
Query43 (2204.1377,1+)	:<no title>
Query44 (2225.1450,1+)	:<no title>
Query45 (2228.1609,1+)	:<no title>
Query46 (2235.1809,1+)	:<no title>
Query47 (2250.1626,1+)	:<no title>
Query48 (2251.1287,1+)	:<no title>
Query49 (2257.1682,1+)	:<no title>
Query50 (2263.1658,1+)	:<no title>
Query51 (2268.2048,1+)	:<no title>
Query52 (2282.1624,1+)	:<no title>
Query53 (2286.2207,1+)	:<no title>
Query54 (2289.2249,1+)	:<no title>
Query55 (2292.1792,1+)	:<no title>
Query56 (2308.1628,1+)	:<no title>
Query57 (2313.1716,1+)	:<no title>
Query58 (2314.1470,1+)	:<no title>
Query59 (2325.2048,1+)	:<no title>
Query60 (2326.2573,1+)	:<no title>
Query61 (2332.1472,1+)	:<no title>
Query62 (2367.2200,1+)	:<no title>
Query63 (2468.2456,1+)	:<no title>
Query64 (2526.2200,1+)	:<no title>
Query65 (2549.3303,1+)	:<no title>
Query66 (2611.2832,1+)	:<no title>
Query67 (3052.6353,1+)	:<no title>
Query68 (3135.5813,1+)	:<no title>
Query69 (3153.5376,1+)	:<no title>

Query70 (3211.5759,1+) :<no title>

Mascot: http://www.matrixscience.com/

Mascot Search Results

Protein View

Band: 1.2

Match to: **B3AT_HUMAN** Score: **274** Expect: **2.2e-022**
Band 3 anion transport protein OS=Homo sapiens GN=SLC4A1 PE=1 SV=3

Nominal mass (Mr): **102013**; Calculated pI value: **5.08**

NCBI BLAST search of B3AT_HUMAN against nr
Unformatted sequence string for pasting into other applications

Taxonomy: Homo sapiens

Fixed modifications: Carbamidomethyl (C)
Variable modifications: Oxidation (M)
Cleavage by Trypsin: cuts C-term side of KR unless next residue is P
Sequence Coverage: **16%**

Matched peptides shown in **Bold Red**

```
  1 MEELQDDYED MMEENLEQEE YEDPDIPESQ MEEPAAHDTE ATATDYHTTS
 51 HPGTHKVYVE LQELVMDEKN QELRWMEAAR WVQLEENLGE NGAWGRPHLS
101 HLTFWSLLEL RRVFTKGTVL LDLQETSLAG VANQLLDRFI FEDQIRPQDR
151 EELLRALLLK HSHAGELEAL GGVKPAVLTR SGDPSQPLLP QHSSLETQLF
201 CEQGDGGTEG HSPSGILEKI PPDSEATLVL VGRADFLEQP VLGFVRLQEA
251 AELEAVELPV PIRFLFVLLG PEAPHIDYTQ LGRAAATLMS ERVFRIDAYM
301 AQSRGELLHS LEGFLDCSLV LPPTDAPSEQ ALLSLVPVQR ELLRRRYQSS
351 PAKPDSSFYK GLDLNGGPDD PLQQTGQLFG GLVRDIRRRY PYYLSDITDA
401 FSPQVLAAVI FIYFAALSPA ITFGGLLGEK TRNQMGVSEL LISTAVQGIL
451 FALLGAQPLL VVGFSGPLLV FEEAFFSFCE TNGLEYIVGR VWIGFWLILL
501 VVLVVAFEGS FLVRFISRYT QEIFSFLISL IFIYETFSKL IKIFQDHPLQ
551 KTYNYNVLMV PKPQGPLPNT ALLSLVLMAG TFFFAMMLRK FKNSSYFPGK
601 LRRVIGDFGV PISILIMVLV DFFIQDTYTQ KLSVPDGFKV SNSSARGWVI
651 HPLGLRSEFP IWMMFASALP ALLVFILIFL ESQITTLIVS KPERKMVKGS
701 GFHLDLLLVV GMGGVAALFG MPWLSATTVR SVTHANALTV MGKASTPGAA
751 AQIQEVKEQR ISGLLVAVLV GLSILMEPIL SRIPLAVLFG IFLYMGVTSL
801 SGIQLFDRIL LLFKPPKYHP DVPYVKRVKT WRMHLFTGIQ IICLAVLWVV
851 KSTPASLALP FVLILTVPLR RVLLPLIFRN VELQCLDADD AKATFDEEEG
901 RDEYDEVAMP V
```

Show predicted peptides also

Sort Peptides By ◉ Residue Number ○ Increasing Mass ○ Decreasing Mass

Start - End	Observed	Mr(expt)	Mr(calc)	ppm	Miss	Sequence	
57 - 74	2235.1809	2234.1736	2234.1252	22	1	K.VYVELQELVMDEKNQELR.W	(No match)
57 - 74	2251.1287	2250.1214	2250.1202	1	1	K.VYVELQELVMDEKNQELR.W	Oxidation (M) (No match)
117 - 138	2326.2573	2325.2500	2325.2540	-2	0	K.GTVLLDLQETSLAGVANQLLDR.F	(No match)
139 - 150	1563.7766	1562.7693	1562.7841	-9	0	R.FIFEDQIRPQDR.E	(No match)
139 - 150	1563.7766	1562.7693	1562.7841	-9	0	R.FIFEDQIRPQDR.E	(Ions score 41)
139 - 155	2204.1377	2203.1304	2203.1385	-4	1	R.FIFEDQIRPQDREELLR.A	(No match)
161 - 180	2042.1002	2041.0929	2041.1069	-7	0	K.HSHAGELEALGGVKPAVLTR.S	(No match)
161 - 180	2042.1002	2041.0929	2041.1069	-7	0	K.HSHAGELEALGGVKPAVLTR.S	(Ions score 78)
220 - 233	1466.8042	1465.7969	1465.8140	-12	0	K.IPPDSEATLVLVGR.A	(No match)
247 - 263	1877.0319	1876.0246	1876.0305	-3	0	R.LQEAAELEAVELPVPIR.F	(No match)
247 - 263	1877.0319	1876.0246	1876.0305	-3	0	R.LQEAAELEAVELPVPIR.F	(Ions score 121)
264 - 283	2286.2207	2285.2134	2285.2208	-3	0	R.FLFVLLGPEAPHIDYTQLGR.A	(No match)
833 - 851	2257.1682	2256.1609	2256.2527	-41	0	R.MHLFTGIQIICLAVLWVVK.S	Oxidation (M) (No match)

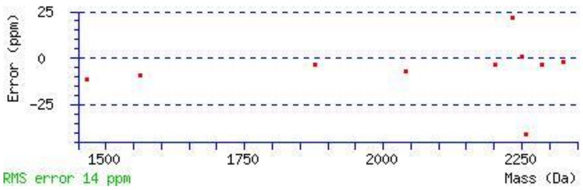

Mascot: http://www.matrixscience.com/

Mascot Search Results

**Band**	: **2**
User	:
Email	:
Search title	: SampleSetID: 759, AnalysisID: 6312, MaldiWellID: 66572, SpectrumID: 144455, Path=\171004\MSMS\17-112 SP combined run 79 y 72
Database	: SwissProt 20170116 (553231 sequences; 197953409 residues)
Timestamp	: 30 Nov 2017 at 07:08:34 GMT
Warning	: A Peptide summary report will usually give a much clearer picture of MS/MS search results.
Top Score	: 201 for HBB_HUMAN, Hemoglobin subunit beta OS=Homo sapiens GN=HBB PE=1 SV=2

Mascot Score Histogram

Protein score is -10*Log(P), where P is the probability that the observed match is a random event.
Protein scores greater than 70 are significant (p<0.05).
Protein scores are derived from ions scores as a non-probabilistic basis for ranking protein hits.

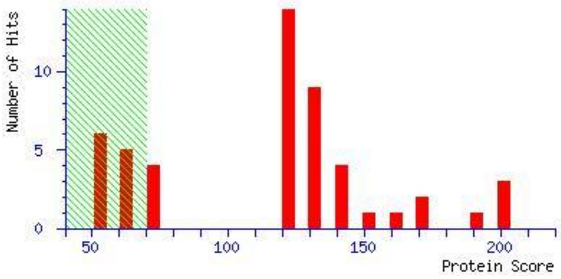

Protein Summary Report

Format As | Protein Summary (deprecated) ▼ | Help

Significance threshold p< 0.05 Max. number of hits 20

Re-Search All | Search Unmatched

Index

	Accession	Mass	Score	Description
1.	HBB_HUMAN	16102	201	Hemoglobin subunit beta OS=Homo sapiens GN=HBB PE=1 SV=2
2.	HBB_PANPA	16102	201	Hemoglobin subunit beta OS=Pan paniscus GN=HBB PE=1 SV=2
3.	HBB_PANTR	16102	201	Hemoglobin subunit beta OS=Pan troglodytes GN=HBB PE=1 SV=2
4.	HBB_GORGO	16074	188	Hemoglobin subunit beta OS=Gorilla gorilla gorilla GN=HBB PE=1 SV=2
5.	HBB_HYLLA	16029	168	Hemoglobin subunit beta OS=Hylobates lar GN=HBB PE=1 SV=1
6.	HBD_SAISC	15858	167	Hemoglobin subunit delta OS=Saimiri sciureus GN=HBD PE=1 SV=1
7.	CASA1_BOVIN	24570164		Alpha-S1-casein OS=Bos taurus GN=CSN1S1 PE=1 SV=2
8.	HBD_SAGMY	15912	153	Hemoglobin subunit delta OS=Saguinus mystax GN=HBD PE=1 SV=1
9.	HBD_SAGNI	15926	141	Hemoglobin subunit delta OS=Saguinus nigricollis GN=HBD PE=1 SV=1
10.	HBB_ATEBE	16148	141	Hemoglobin subunit beta OS=Ateles belzebuth GN=HBB PE=1 SV=2
11.	HBB_ATEGE	16148	141	Hemoglobin subunit beta OS=Ateles geoffroyi GN=HBB PE=1 SV=2
12.	HBB_LAGLA	16120	140	Hemoglobin subunit beta OS=Lagothrix lagotricha GN=HBB PE=2 SV=3
13.	HBD_AOTTR	15867	130	Hemoglobin subunit delta OS=Aotus trivirgatus GN=HBD PE=1 SV=1
14.	HBB_AOTTR	16002	128	Hemoglobin subunit beta OS=Aotus trivirgatus GN=HBB PE=1 SV=1
15.	HBB_SEMEN	15999	128	Hemoglobin subunit beta OS=Semnopithecus entellus GN=HBB PE=1 SV=1
16.	HBD_ATEFU	15882	128	Hemoglobin subunit delta OS=Ateles fusciceps GN=HBD PE=1 SV=1
17.	HBB_ALOBE	16161	128	Hemoglobin subunit beta OS=Alouatta belzebul GN=HBB PE=2 SV=3
18.	HBB_ATEPA	16150	128	Hemoglobin subunit beta OS=Ateles paniscus GN=HBB PE=1 SV=3
19.	HBB_PITPI	16147	128	Hemoglobin subunit beta OS=Pithecia pithecia GN=HBB PE=2 SV=3
20.	HBB_SAISC	16147	128	Hemoglobin subunit beta OS=Saimiri sciureus GN=HBB PE=1 SV=2

Results List

1. HBB_HUMAN **Mass:** 16102 **Score:** 201 **Expect:** 4.4e-015 **Matches:** 13

Hemoglobin subunit beta OS=Homo sapiens GN=HBB PE=1 SV=2

Observed	Mr(expt)	Mr(calc)	ppm	Start		End	Miss	Ions	Peptide
1126.5620	1125.5547	1125.5567	-1.76	97	-	105	0	---	K.LHVDPENFR.L
1274.7185	1273.7112	1273.7183	-5.55	32	-	41	0	---	R.LLVVYPWTQR.F
1314.6691	1313.6618	1313.6575	3.26	19	-	31	0	---	K.VNVDEVGGEALGR.L
1378.6844	1377.6771	1377.6929	-11.42	122	-	133	0	---	K.EFTPPVQAAYQK.V
1378.6844	1377.6771	1377.6929	-11.42	122	-	133	0	20	K.EFTPPVQAAYQK.V
1669.8772	1668.8699	1668.8835	-8.14	68	-	83	0	---	K.VLGAFSDGLAHLDNLK.G
1669.8772	1668.8699	1668.8835	-8.14	68	-	83	0	(77)	K.VLGAFSDGLAHLDNLK.G
1669.8772	1668.8699	1668.8835	-8.14	68	-	83	0	91	K.VLGAFSDGLAHLDNLK.G
1797.9766	1796.9693	1796.9785	-5.09	67	-	83	1	---	K.KVLGAFSDGLAHLDNLK.G
2058.9866	2057.9793	2057.9405	18.9	42	-	60	0	---	R.FFESFGDLSTPDAVMGNPK.V
2074.9434	2073.9361	2073.9354	0.36	42	-	60	0	---	R.FFESFGDLSTPDAVMGNPK.V + Oxidation (M)
2228.1665	2227.1592	2227.1597	-0.21	10	-	31	1	---	K.SAVTALWGKVNVDEVGGEALGR.L

2286.1941	2285.1868	2285.1038	36.3	42	62	1	---	R.FFESFGDLSTPDAVMGNPKVK.A

No match to: 979.5460, 1034.1182, 1147.5027, 1195.6837, 1195.6837, 1195.6837, 1205.6095, 1205.6095, 1219.6508, 1219.6508, 1219.6508, 1252.6511, 1267.7006, 1337.6794,1337.6794, 1342.6943, 1360.6827, 1365.6962, 1367.6936, 1383.7865, 1384.7319, 1475.7609, 1529.7290, 1557.7811, 1563.7914, 1572.7805,1572.7805, 1575.7814, 1585.7865, 1641.8524, 1648.8374, 1742.8954, 1759.9335, 1763.8998, 1771.9310, 1787.9608, 1811.9208, 1816.9521,1853.9644, 1868.9490, 1871.9698, 1877.0233, 1881.0533, 1881.0533, 1929.9923, 1951.9395, 1994.1302, 2009.0477, 2042.1072, 2042.1072,2107.2268, 2138.1482, 2184.1758, 2202.1494, 2225.1511, 2235.2302, 2235.2302, 2268.2280, 2291.1104, 2314.1516, 2326.2490, 2330.1890,2332.1421, 2360.1626, 3223.6489

2. HBB_PANPA **Mass:** 16102 **Score:** 201 **Expect:** 4.4e-015 **Matches:** 13

Hemoglobin subunit beta OS=Pan paniscus GN=HBB PE=1 SV=2

Observed	Mr(expt)	Mr(calc)	ppm	Start	End	Miss	Ions	Peptide
1126.5620	1125.5547	1125.5567	-1.76	97	105	0	---	K.LHVDPENFR.L
1274.7185	1273.7112	1273.7183	-5.55	32	41	0	---	R.LLVVYPWTQR.F
1314.6691	1313.6618	1313.6575	3.26	19	31	0	---	K.VNVDEVGGEALGR.L
1378.6844	1377.6771	1377.6929	-11.42	122	133	0	---	K.EFTPPVQAAYQK.V
1378.6844	1377.6771	1377.6929	-11.42	122	133	0	20	K.EFTPPVQAAYQK.V
1669.8772	1668.8699	1668.8835	-8.14	68	83	0	---	K.VLGAFSDGLAHLDNLK.G
1669.8772	1668.8699	1668.8835	-8.14	68	83	0	(77)	K.VLGAFSDGLAHLDNLK.G
1669.8772	1668.8699	1668.8835	-8.14	68	83	0	91	K.VLGAFSDGLAHLDNLK.G
1797.9766	1796.9693	1796.9785	-5.09	67	83	1	---	K.KVLGAFSDGLAHLDNLK.G
2058.9866	2057.9793	2057.9405	18.9	42	60	0	---	R.FFESFGDLSTPDAVMGNPK.V
2074.9434	2073.9361	2073.9354	0.36	42	60	0	---	R.FFESFGDLSTPDAVMGNPK.V + Oxidation (M)
2228.1665	2227.1592	2227.1597	-0.21	10	31	1	---	K.SAVTALWGKVNVDEVGGEALGR.L
2286.1941	2285.1868	2285.1038	36.3	42	62	1	---	R.FFESFGDLSTPDAVMGNPKVK.A

No match to: 979.5460, 1034.1182, 1147.5027, 1195.6837, 1195.6837, 1195.6837, 1205.6095, 1205.6095, 1219.6508, 1219.6508, 1219.6508, 1252.6511, 1267.7006, 1337.6794, 1337.6794, 1342.6943, 1360.6827, 1365.6962, 1367.6936, 1383.7865, 1384.7319, 1475.7609, 1529.7290, 1557.7811, 1563.7914, 1572.7805, 1572.7805, 1575.7814, 1585.7865, 1641.8524, 1648.8374, 1742.8954, 1759.9335, 1763.8998, 1771.9310, 1787.9608, 1811.9208, 1816.9521, 1853.9644, 1868.9490, 1871.9698, 1877.0233, 1881.0533, 1881.0533, 1929.9923, 1951.9395, 1994.1302, 2009.0477, 2042.1072, 2042.1072, 2107.2268, 2138.1482, 2184.1758, 2202.1494, 2225.1511, 2235.2302, 2235.2302, 2268.2280, 2291.1104, 2314.1516, 2326.2490, 2330.1890, 2332.1421, 2360.1626, 3223.6489

3. HBB_PANTR **Mass:** 16102 **Score:** 201 **Expect:** 4.4e-015 **Matches:** 13

Hemoglobin subunit beta OS=Pan troglodytes GN=HBB PE=1 SV=2

Observed	Mr(expt)	Mr(calc)	ppm	Start	End	Miss	Ions	Peptide
1126.5620	1125.5547	1125.5567	-1.76	97	105	0	---	K.LHVDPENFR.L
1274.7185	1273.7112	1273.7183	-5.55	32	41	0	---	R.LLVVYPWTQR.F
1314.6691	1313.6618	1313.6575	3.26	19	31	0	---	K.VNVDEVGGEALGR.L
1378.6844	1377.6771	1377.6929	-11.42	122	133	0	---	K.EFTPPVQAAYQK.V
1378.6844	1377.6771	1377.6929	-11.42	122	133	0	20	K.EFTPPVQAAYQK.V
1669.8772	1668.8699	1668.8835	-8.14	68	83	0	---	K.VLGAFSDGLAHLDNLK.G
1669.8772	1668.8699	1668.8835	-8.14	68	83	0	(77)	K.VLGAFSDGLAHLDNLK.G
1669.8772	1668.8699	1668.8835	-8.14	68	83	0	91	K.VLGAFSDGLAHLDNLK.G
1797.9766	1796.9693	1796.9785	-5.09	67	83	1	---	K.KVLGAFSDGLAHLDNLK.G
2058.9866	2057.9793	2057.9405	18.9	42	60	0	---	R.FFESFGDLSTPDAVMGNPK.V
2074.9434	2073.9361	2073.9354	0.36	42	60	0	---	R.FFESFGDLSTPDAVMGNPK.V + Oxidation (M)
2228.1665	2227.1592	2227.1597	-0.21	10	31	1	---	K.SAVTALWGKVNVDEVGGEALGR.L
2286.1941	2285.1868	2285.1038	36.3	42	62	1	---	R.FFESFGDLSTPDAVMGNPKVK.A

No match to: 979.5460, 1034.1182, 1147.5027, 1195.6837, 1195.6837, 1195.6837, 1205.6095, 1205.6095, 1219.6508, 1219.6508, 1219.6508, 1252.6511, 1267.7006, 1337.6794, 1337.6794, 1342.6943, 1360.6827, 1365.6962, 1367.6936, 1383.7865, 1384.7319, 1475.7609, 1529.7290, 1557.7811, 1563.7914, 1572.7805, 1572.7805, 1575.7814, 1585.7865, 1641.8524, 1648.8374, 1742.8954, 1759.9335, 1763.8998, 1771.9310, 1787.9608, 1811.9208, 1816.9521, 1853.9644, 1868.9490, 1871.9698, 1877.0233, 1881.0533, 1881.0533, 1929.9923, 1951.9395, 1994.1302, 2009.0477, 2042.1072, 2042.1072, 2107.2268, 2138.1482, 2184.1758, 2202.1494, 2225.1511, 2235.2302, 2235.2302, 2268.2280, 2291.1104, 2314.1516, 2326.2490, 2330.1890, 2332.1421, 2360.1626, 3223.6489

4. HBB_GORGO **Mass:** 16074 **Score:** 188 **Expect:** 8.8e-014 **Matches:** 12

Hemoglobin subunit beta OS=Gorilla gorilla gorilla GN=HBB PE=1 SV=2

Observed	Mr(expt)	Mr(calc)	ppm	Start	End	Miss	Ions	Peptide
1274.7185	1273.7112	1273.7183	-5.55	32	41	0	---	R.LLVVYPWTQR.F
1314.6691	1313.6618	1313.6575	3.26	19	31	0	---	K.VNVDEVGGEALGR.L
1378.6844	1377.6771	1377.6929	-11.42	122	133	0	---	K.EFTPPVQAAYQK.V
1378.6844	1377.6771	1377.6929	-11.42	122	133	0	20	K.EFTPPVQAAYQK.V
1669.8772	1668.8699	1668.8835	-8.14	68	83	0	---	K.VLGAFSDGLAHLDNLK.G
1669.8772	1668.8699	1668.8835	-8.14	68	83	0	(77)	K.VLGAFSDGLAHLDNLK.G
1669.8772	1668.8699	1668.8835	-8.14	68	83	0	91	K.VLGAFSDGLAHLDNLK.G
1797.9766	1796.9693	1796.9785	-5.09	67	83	1	---	K.KVLGAFSDGLAHLDNLK.G
2058.9866	2057.9793	2057.9405	18.9	42	60	0	---	R.FFESFGDLSTPDAVMGNPK.V
2074.9434	2073.9361	2073.9354	0.36	42	60	0	---	R.FFESFGDLSTPDAVMGNPK.V + Oxidation (M)
2228.1665	2227.1592	2227.1597	-0.21	10	31	1	---	K.SAVTALWGKVNVDEVGGEALGR.L
2286.1941	2285.1868	2285.1038	36.3	42	62	1	---	R.FFESFGDLSTPDAVMGNPKVK.A

No match to: 979.5460, 1034.1182, 1126.5620, 1147.5027, 1195.6837, 1195.6837, 1195.6837, 1205.6095, 1205.6095, 1219.6508, 1219.6508, 1219.6508, 1252.6511, 1267.7006, 1337.6794, 1337.6794, 1342.6943, 1360.6827, 1365.6962, 1367.6936, 1383.7865, 1384.7319, 1475.7609, 1529.7290, 1557.7811, 1563.7914, 1572.7805, 1572.7805, 1575.7814, 1585.7865, 1641.8524, 1648.8374, 1742.8954, 1759.9335, 1763.8998, 1771.9310, 1787.9608, 1811.9208, 1816.9521, 1853.9644, 1868.9490, 1871.9698, 1877.0233, 1881.0533, 1881.0533, 1929.9923, 1951.9395, 1994.1302, 2009.0477, 2042.1072, 2042.1072, 2107.2268, 2138.1482, 2184.1758, 2202.1494, 2225.1511, 2235.2302, 2235.2302, 2268.2280, 2291.1104, 2314.1516, 2326.2490, 2330.1890, 2332.1421, 2360.1626, 3223.6489

5. HBB_HYLLA **Mass:** 16029 **Score:** 168 **Expect:** 8.8e-012 **Matches:** 11

Hemoglobin subunit beta OS=Hylobates lar GN=HBB PE=1 SV=1

Observed	Mr(expt)	Mr(calc)	ppm	Start	End	Miss	Ions	Peptide

1126.5620	1125.5547	1125.5567	-1.76	96	104	0	---	K.LHVDPENFR.L
1274.7185	1273.7112	1273.7183	-5.55	31	40	0	---	R.LLVVYPWTQR.F
1314.6691	1313.6618	1313.6575	3.26	18	30	0	---	K.VNVDEVGGEALGR.L
1669.8772	1668.8699	1668.8835	-8.14	67	82	0	---	K.VLGAFSDGLAHLDNLK.G
1669.8772	1668.8699	1668.8835	-8.14	67	82	0	(77)	K.VLGAFSDGLAHLDNLK.G
1669.8772	1668.8699	1668.8835	-8.14	67	82	0	91	K.VLGAFSDGLAHLDNLK.G
1797.9766	1796.9693	1796.9785	-5.09	66	82	1	---	K.KVLGAFSDGLAHLDNLK.G
2058.9866	2057.9793	2057.9405	18.9	41	59	0	---	R.FFESFGDLSTPDAVMGNPK.V
2074.9434	2073.9361	2073.9354	0.36	41	59	0	---	R.FFESFGDLSTPDAVMGNPK.V + Oxidation (M)
2228.1665	2227.1592	2227.1597	-0.21	9	30	1	---	K.SAVTALWGKVNVDEVGGEALGR.L
2286.1941	2285.1868	2285.1038	36.3	41	61	1	---	R.FFESFGDLSTPDAVMGNPKVK.A

No match to: 979.5460, 1034.1182, 1147.5027, 1195.6837, 1195.6837, 1195.6837, 1205.6095, 1205.6095, 1219.6508, 1219.6508, 1219.6508, 1252.6511, 1267.7006, 1337.6794, 1337.6794, 1342.6943, 1360.6827, 1365.6962, 1367.6936, 1378.6844, 1378.6844, 1383.7865, 1384.7319, 1475.7609, 1529.7290, 1557.7811, 1563.7914, 1572.7805, 1572.7805, 1575.7814, 1585.7865, 1641.8524, 1648.8374, 1742.8954, 1759.9335, 1763.8998, 1771.9310, 1787.9608, 1811.9208, 1816.9521, 1853.9644, 1868.9490, 1871.9698, 1877.0233, 1881.0533, 1881.0533, 1929.9923, 1951.9395, 1994.1302, 2009.0477, 2042.1072, 2042.1072, 2107.2268, 2138.1482, 2184.1758, 2202.1494, 2225.1511, 2235.2302, 2235.2302, 2268.2280, 2291.1104, 2314.1516, 2326.2490, 2330.1890, 2332.1421, 2360.1626, 3223.6489

6. HBD_SAISC **Mass:** 15858 **Score:** 167 **Expect:** 1.1e-011 **Matches:** 10

Hemoglobin subunit delta OS=Saimiri sciureus GN=HBD PE=1 SV=1

Observed	Mr(expt)	Mr(calc)	ppm	Start	End	Miss	Ions	Peptide
1126.5620	1125.5547	1125.5567	-1.76	96	104	0	---	K.LHVDPENFR.L
1274.7185	1273.7112	1273.7183	-5.55	31	40	0	---	R.LLVVYPWTQR.F
1314.6691	1313.6618	1313.6575	3.26	18	30	0	---	K.VNVDEVGGEALGR.L
1669.8772	1668.8699	1668.8835	-8.14	67	82	0	---	K.VLGAFSDGLAHLDNLK.G
1669.8772	1668.8699	1668.8835	-8.14	67	82	0	(77)	K.VLGAFSDGLAHLDNLK.G
1669.8772	1668.8699	1668.8835	-8.14	67	82	0	91	K.VLGAFSDGLAHLDNLK.G
1797.9766	1796.9693	1796.9785	-5.09	66	82	1	---	K.KVLGAFSDGLAHLDNLK.G
1811.9208	1810.9135	1810.9577	-24.41	1	17	1	---	-.VHLTGDEKSAVAALWSK.V
2202.1494	2201.1421	2201.0827	27.0	41	61	1	---	R.FFESFGALSSADAVMGNPKVK.A
2228.1665	2227.1592	2227.1597	-0.21	9	30	1	---	K.SAVAALWSKVNVDEVGGEALGR.L

No match to: 979.5460, 1034.1182, 1147.5027, 1195.6837, 1195.6837, 1195.6837, 1205.6095, 1205.6095, 1219.6508, 1219.6508, 1219.6508, 1252.6511, 1267.7006, 1337.6794, 1337.6794, 1342.6943, 1360.6827, 1365.6962, 1367.6936, 1378.6844, 1378.6844, 1383.7865, 1384.7319, 1475.7609, 1529.7290, 1557.7811, 1563.7914, 1572.7805, 1572.7805, 1575.7814, 1585.7865, 1641.8524, 1648.8374, 1742.8954, 1759.9335, 1763.8998, 1771.9310, 1787.9608, 1816.9521, 1853.9644, 1868.9490, 1871.9698, 1877.0233, 1881.0533, 1881.0533, 1929.9923, 1951.9395, 1994.1302, 2009.0477, 2042.1072, 2042.1072, 2058.9866, 2074.9434, 2107.2268, 2138.1482, 2184.1758, 2225.1511, 2235.2302, 2235.2302, 2268.2280, 2286.1941, 2291.1104, 2314.1516, 2326.2490, 2330.1890, 2332.1421, 2360.1626, 3223.6489

7. CASA1_BOVIN **Mass:** 24570 **Score:** 164 **Expect:** 2.2e-011 **Matches:** 11

Alpha-S1-casein OS=Bos taurus GN=CSN1S1 PE=1 SV=2

Observed	Mr(expt)	Mr(calc)	ppm	Start	End	Miss	Ions	Peptide
1267.7006	1266.6933	1266.6972	-3.05	106	115	0	---	R.YLGYLEQLLR.L
1337.6794	1336.6721	1336.6735	-1.04	95	105	1	---	K.HIQKEDVPSER.Y
1337.6794	1336.6721	1336.6735	-1.04	95	105	1	42	K.HIQKEDVPSER.Y
1384.7319	1383.7246	1383.7227	1.37	38	49	0	---	R.FFVAPFPEVFGK.E
1641.8524	1640.8451	1640.8603	-9.23	38	51	1	---	R.FFVAPFPEVFGKEK.V
1759.9335	1758.9262	1758.9377	-6.50	23	37	0	---	K.HQGLPQEVLNENLLR.F
1871.9698	1870.9625	1870.9788	-8.72	119	134	1	---	K.YKVPQLEIVPNSAEER.L
2235.2302	2234.2229	2234.2283	-2.43	19	37	1	---	K.HPIKHQGLPQEVLNENLLR.F
2235.2302	2234.2229	2234.2283	-2.43	19	37	1	51	K.HPIKHQGLPQEVLNENLLR.F
2332.1421	2331.1348	2331.1245	4.42	148	166	0	---	K.EPMIGVNQELAYFYPELFR.Q + Oxidation (M)
3223.6489	3222.6416	3222.5808	18.9	140	166	1	---	K.EGIHAQQKEPMIGVNQELAYFYPELFR.Q + Oxidation (M)

No match to: 979.5460, 1034.1182, 1126.5620, 1147.5027, 1195.6837, 1195.6837, 1195.6837, 1205.6095, 1205.6095, 1219.6508, 1219.6508, 1219.6508, 1252.6511, 1274.7185, 1314.6691, 1342.6943, 1360.6827, 1365.6962, 1367.6936, 1378.6844, 1378.6844, 1383.7865, 1475.7609, 1529.7290, 1557.7811, 1563.7914, 1572.7805, 1572.7805, 1575.7814, 1585.7865, 1648.8374, 1669.8772, 1669.8772, 1669.8772, 1742.8954, 1763.8998, 1771.9310, 1787.9608, 1797.9766, 1811.9208, 1816.9521, 1853.9644, 1868.9490, 1877.0233, 1881.0533, 1881.0533, 1929.9923, 1951.9395, 1994.1302, 2009.0477, 2042.1072, 2042.1072, 2058.9866, 2074.9434, 2107.2268, 2138.1482, 2184.1758, 2202.1494, 2225.1511, 2228.1665, 2268.2280, 2286.1941, 2291.1104, 2314.1516, 2326.2490, 2330.1890, 2360.1626

8. HBD_SAGMY **Mass:** 15912 **Score:** 153 **Expect:** 2.8e-010 **Matches:** 9

Hemoglobin subunit delta OS=Saguinus mystax GN=HBD PE=1 SV=1

Observed	Mr(expt)	Mr(calc)	ppm	Start	End	Miss	Ions	Peptide
1126.5620	1125.5547	1125.5567	-1.76	96	104	0	---	K.LHVDPENFR.L
1274.7185	1273.7112	1273.7183	-5.55	31	40	0	---	R.LLVVYPWTQR.F
1314.6691	1313.6618	1313.6575	3.26	18	30	0	---	K.VNVDEVGGEALGR.L
1669.8772	1668.8699	1668.8835	-8.14	67	82	0	---	K.VLGAFSDGLAHLDNLK.G
1669.8772	1668.8699	1668.8835	-8.14	67	82	0	(77)	K.VLGAFSDGLAHLDNLK.G
1669.8772	1668.8699	1668.8835	-8.14	67	82	0	91	K.VLGAFSDGLAHLDNLK.G
1797.9766	1796.9693	1796.9785	-5.09	66	82	1	---	K.KVLGAFSDGLAHLDNLK.G
1811.9208	1810.9135	1810.9577	-24.41	1	17	1	---	-.VHLTGDEKSAVAALWSK.V
2228.1665	2227.1592	2227.1597	-0.21	9	30	1	---	K.SAVAALWSKVNVDEVGGEALGR.L

No match to: 979.5460, 1034.1182, 1147.5027, 1195.6837, 1195.6837, 1195.6837, 1205.6095, 1205.6095, 1219.6508, 1219.6508, 1219.6508, 1252.6511, 1267.7006, 1337.6794, 1337.6794, 1342.6943, 1360.6827, 1365.6962, 1367.6936, 1378.6844, 1378.6844, 1383.7865, 1384.7319, 1475.7609, 1529.7290, 1557.7811, 1563.7914, 1572.7805, 1572.7805, 1575.7814, 1585.7865, 1641.8524, 1648.8374, 1742.8954, 1759.9335, 1763.8998, 1771.9310, 1787.9608, 1816.9521, 1853.9644, 1868.9490, 1871.9698, 1877.0233, 1881.0533, 1881.0533, 1929.9923, 1951.9395, 1994.1302, 2009.0477, 2042.1072, 2042.1072, 2058.9866, 2074.9434, 2107.2268, 2138.1482, 2184.1758, 2202.1494, 2225.1511, 2235.2302, 2235.2302, 2268.2280, 2286.1941, 2291.1104, 2314.1516, 2326.2490, 2330.1890, 2332.1421, 2360.1626, 3223.6489

9. HBD_SAGNI **Mass:** 15926 **Score:** 141 **Expect:** 4.4e-009 **Matches:** 8

Hemoglobin subunit delta OS=Saguinus nigricollis GN=HBDPE=1 SV=1

Observed	Mr(expt)	Mr(calc)	ppm	Start	End	Miss	Ions	Peptide
1126.5620	1125.5547	1125.5567	-1.76	96 -	104	0	---	K.LHVDPENFR.L
1274.7185	1273.7112	1273.7183	-5.55	31 -	40	0	---	R.LLVVYPWTQR.F
1314.6691	1313.6618	1313.6575	3.26	18 -	30	0	---	K.VNVDEVGGEALGR.L
1669.8772	1668.8699	1668.8835	-8.14	67 -	82	0	---	K.VLGAFSDGLAHLDNLK.G
1669.8772	1668.8699	1668.8835	-8.14	67 -	82	0	(77)	K.VLGAFSDGLAHLDNLK.G
1669.8772	1668.8699	1668.8835	-8.14	67 -	82	0	91	K.VLGAFSDGLAHLDNLK.G
1797.9766	1796.9693	1796.9785	-5.09	66 -	82	1	---	K.KVLGAFSDGLAHLDNLK.G
2228.1665	2227.1592	2227.1597	-0.21	9 -	30	1	---	K.SAVAALWSKVNVDEVGGEALGR.L

No match to: 979.5460, 1034.1182, 1147.5027, 1195.6837, 1195.6837, 1195.6837, 1205.6095, 1205.6095, 1219.6508, 1219.6508, 1219.6508, 1252.6511, 1267.7006, 1337.6794, 1337.6794, 1342.6943, 1360.6827, 1365.6962, 1367.6936, 1378.6844, 1378.6844, 1383.7865, 1384.7319, 1475.7609, 1529.7290, 1557.7811, 1563.7914, 1572.7805, 1572.7805, 1575.7814, 1585.7865, 1641.8524, 1648.8374, 1742.8954, 1759.9335, 1763.8998, 1771.9310, 1787.9608, 1811.9208, 1816.9521, 1853.9644, 1868.9490, 1871.9698, 1877.0233, 1881.0533, 1881.0533, 1929.9923, 1951.9395, 1994.1302, 2009.0477, 2042.1072, 2042.1072, 2058.9866, 2074.9434, 2107.2268, 2138.1482, 2184.1758, 2202.1494, 2225.1511, 2235.2302, 2235.2302, 2268.2280, 2286.1941, 2291.1104, 2314.1516, 2326.2490, 2330.1890, 2332.1421, 2360.1626, 3223.6489

10. HBB_ATEBE **Mass:** 16148 **Score:** 141 **Expect:** 4.4e-009 **Matches:** 8

Hemoglobin subunit beta OS=Ateles belzebuth GN=HBB PE=1 SV=2

Observed	Mr(expt)	Mr(calc)	ppm	Start	End	Miss	Ions	Peptide
1126.5620	1125.5547	1125.5567	-1.76	97 -	105	0	---	K.LHVDPENFR.L
1274.7185	1273.7112	1273.7183	-5.55	32 -	41	0	---	R.LLVVYPWTQR.F
1314.6691	1313.6618	1313.6575	3.26	19 -	31	0	---	K.VNVDEVGGEALGR.L
1669.8772	1668.8699	1668.8835	-8.14	68 -	83	0	---	K.VLGAFSDGLAHLDNLK.G
1669.8772	1668.8699	1668.8835	-8.14	68 -	83	0	(77)	K.VLGAFSDGLAHLDNLK.G
1669.8772	1668.8699	1668.8835	-8.14	68 -	83	0	91	K.VLGAFSDGLAHLDNLK.G
1797.9766	1796.9693	1796.9785	-5.09	67 -	83	1	---	K.KVLGAFSDGLAHLDNLK.G
2332.1421	2331.1348	2331.1093	10.9	42 -	62	1	---	R.FFESFGDLSTPDAVMSNPKVK.A + Oxidation (M)

No match to: 979.5460, 1034.1182, 1147.5027, 1195.6837, 1195.6837, 1195.6837, 1205.6095, 1205.6095, 1219.6508, 1219.6508, 1219.6508, 1252.6511, 1267.7006, 1337.6794, 1337.6794, 1342.6943, 1360.6827, 1365.6962, 1367.6936, 1378.6844, 1378.6844, 1383.7865, 1384.7319, 1475.7609, 1529.7290, 1557.7811, 1563.7914, 1572.7805, 1572.7805, 1575.7814, 1585.7865, 1641.8524, 1648.8374, 1742.8954, 1759.9335, 1763.8998, 1771.9310, 1787.9608, 1811.9208, 1816.9521, 1853.9644, 1868.9490, 1871.9698, 1877.0233, 1881.0533, 1881.0533, 1929.9923, 1951.9395, 1994.1302, 2009.0477, 2042.1072, 2042.1072, 2058.9866, 2074.9434, 2107.2268, 2138.1482, 2184.1758, 2202.1494, 2225.1511, 2228.1665, 2235.2302, 2235.2302, 2268.2280, 2286.1941, 2291.1104, 2314.1516, 2326.2490, 2330.1890, 2360.1626, 3223.6489

11. HBB_ATEGE **Mass:** 16148 **Score:** 141 **Expect:** 4.4e-009 **Matches:** 8

Hemoglobin subunit beta OS=Ateles geoffroyi GN=HBB PE=1 SV=2

Observed	Mr(expt)	Mr(calc)	ppm	Start	End	Miss	Ions	Peptide
1126.5620	1125.5547	1125.5567	-1.76	97 -	105	0	---	K.LHVDPENFR.L
1274.7185	1273.7112	1273.7183	-5.55	32 -	41	0	---	R.LLVVYPWTQR.F
1314.6691	1313.6618	1313.6575	3.26	19 -	31	0	---	K.VNVDEVGGEALGR.L
1669.8772	1668.8699	1668.8835	-8.14	68 -	83	0	---	K.VLGAFSDGLAHLDNLK.G
1669.8772	1668.8699	1668.8835	-8.14	68 -	83	0	(77)	K.VLGAFSDGLAHLDNLK.G
1669.8772	1668.8699	1668.8835	-8.14	68 -	83	0	91	K.VLGAFSDGLAHLDNLK.G
1797.9766	1796.9693	1796.9785	-5.09	67 -	83	1	---	K.KVLGAFSDGLAHLDNLK.G
2332.1421	2331.1348	2331.1093	10.9	42 -	62	1	---	R.FFESFGDLSTPDAVMSNPKVK.A + Oxidation (M)

No match to: 979.5460, 1034.1182, 1147.5027, 1195.6837, 1195.6837, 1195.6837, 1205.6095, 1205.6095, 1219.6508, 1219.6508, 1219.6508, 1252.6511, 1267.7006, 1337.6794, 1337.6794, 1342.6943, 1360.6827, 1365.6962, 1367.6936, 1378.6844, 1378.6844, 1383.7865, 1384.7319, 1475.7609, 1529.7290, 1557.7811, 1563.7914, 1572.7805, 1572.7805, 1575.7814, 1585.7865, 1641.8524, 1648.8374, 1742.8954, 1759.9335, 1763.8998, 1771.9310, 1787.9608, 1811.9208, 1816.9521, 1853.9644, 1868.9490, 1871.9698, 1877.0233, 1881.0533, 1881.0533, 1929.9923, 1951.9395, 1994.1302, 2009.0477, 2042.1072, 2042.1072, 2058.9866, 2074.9434, 2107.2268, 2138.1482, 2184.1758, 2202.1494, 2225.1511, 2228.1665, 2235.2302, 2235.2302, 2268.2280, 2286.1941, 2291.1104, 2314.1516, 2326.2490, 2330.1890, 2360.1626, 3223.6489

12. HBB_LAGLA **Mass:** 16120 **Score:** 140 **Expect:** 5.5e-009 **Matches:** 8

Hemoglobin subunit beta OS=Lagothrix lagotricha GN=HBB PE=2 SV=3

Observed	Mr(expt)	Mr(calc)	ppm	Start	End	Miss	Ions	Peptide
1126.5620	1125.5547	1125.5567	-1.76	97 -	105	0	---	K.LHVDPENFR.L
1274.7185	1273.7112	1273.7183	-5.55	32 -	41	0	---	R.LLVVYPWTQR.F
1314.6691	1313.6618	1313.6575	3.26	19 -	31	0	---	K.VNVDEVGGEALGR.L
1669.8772	1668.8699	1668.8835	-8.14	68 -	83	0	---	K.VLGAFSDGLAHLDNLK.G
1669.8772	1668.8699	1668.8835	-8.14	68 -	83	0	(77)	K.VLGAFSDGLAHLDNLK.G
1669.8772	1668.8699	1668.8835	-8.14	68 -	83	0	91	K.VLGAFSDGLAHLDNLK.G
1797.9766	1796.9693	1796.9785	-5.09	67 -	83	1	---	K.KVLGAFSDGLAHLDNLK.G
2074.9434	2073.9361	2073.9354	0.36	42 -	60	0	---	R.FFDSFGDLSTPDAVMSNPK.V

No match to: 979.5460, 1034.1182, 1147.5027, 1195.6837, 1195.6837, 1195.6837, 1205.6095, 1205.6095, 1219.6508, 1219.6508, 1219.6508, 1252.6511, 1267.7006, 1337.6794, 1337.6794, 1342.6943, 1360.6827, 1365.6962, 1367.6936, 1378.6844, 1378.6844, 1383.7865, 1384.7319, 1475.7609, 1529.7290, 1557.7811, 1563.7914, 1572.7805, 1572.7805, 1575.7814, 1585.7865, 1641.8524, 1648.8374, 1742.8954, 1759.9335, 1763.8998, 1771.9310, 1787.9608, 1811.9208, 1816.9521, 1853.9644, 1868.9490, 1871.9698, 1877.0233, 1881.0533, 1881.0533, 1929.9923, 1951.9395, 1994.1302, 2009.0477, 2042.1072, 2042.1072, 2058.9866, 2107.2268, 2138.1482, 2184.1758, 2202.1494, 2225.1511, 2228.1665, 2235.2302, 2235.2302, 2268.2280, 2286.1941, 2291.1104, 2314.1516, 2326.2490, 2330.1890, 2332.1421, 2360.1626, 3223.6489

13. HBD_AOTTR **Mass:** 15867 **Score:** 130 **Expect:** 5.5e-008 **Matches:** 7

Hemoglobin subunit delta OS=Aotus trivirgatus GN=HBD PE=1 SV=1

Observed	Mr(expt)	Mr(calc)	ppm	Start	End	Miss	Ions	Peptide
1126.5620	1125.5547	1125.5567	-1.76	96 -	104	0	---	K.LHVDPENFR.L
1274.7185	1273.7112	1273.7183	-5.55	31 -	40	0	---	R.LLVVYPWTQR.F
1669.8772	1668.8699	1668.8835	-8.14	67 -	82	0	---	K.VLGAFSDGLAHLDNLK.G
1669.8772	1668.8699	1668.8835	-8.14	67 -	82	0	(77)	K.VLGAFSDGLAHLDNLK.G
1669.8772	1668.8699	1668.8835	-8.14	67 -	82	0	91	K.VLGAFSDGLAHLDNLK.G
1797.9766	1796.9693	1796.9785	-5.09	66 -	82	1	---	K.KVLGAFSDGLAHLDNLK.G

2228.1665	2227.1592	2227.0983	27.3	41	61	1	---	R.FFESFGALSSPDAVMGNPKVK.A

No match to: 979.5460, 1034.1182, 1147.5027, 1195.6837, 1195.6837, 1195.6837, 1205.6095, 1205.6095, 1219.6508, 1219.6508, 1219.6508, 1252.6511, 1267.7006, 1314.6691, 1337.6794, 1337.6794, 1342.6943, 1360.6827, 1365.6962, 1367.6936,1378.6844, 1378.6844, 1383.7865, 1384.7319, 1475.7609, 1529.7290, 1557.7811, 1563.7914, 1572.7805, 1572.7805, 1575.7814, 1585.7865,1641.8524, 1648.8374, 1742.8954, 1759.9335, 1763.8998, 1771.9310, 1787.9608, 1811.9208, 1816.9521, 1853.9644, 1868.9490, 1871.9698,1877.0233, 1881.0533, 1881.0533, 1929.9923, 1951.9395, 1994.1302, 2009.0477, 2042.1072, 2042.1072, 2058.9866, 2074.9434, 2107.2268,2138.1482, 2184.1758, 2202.1494, 2225.1511, 2235.2302, 2235.2302, 2268.2280, 2286.1941, 2291.1104, 2314.1516, 2326.2490, 2330.1890,2332.1421, 2360.1626, 3223.6489

14. HBB_AOTTR **Mass:** 16002 **Score:** 128**Expect:** 8.8e-008 **Matches:** 7

Hemoglobin subunit beta OS=Aotus trivirgatus GN=HBB PE=1 SV=1

Observed	Mr(expt)	Mr(calc)	ppm	Start	End	Miss	Ions	Peptide
1126.5620	1125.5547	1125.5567	-1.76	96	104	0	---	K.LHVDPENFR.L
1274.7185	1273.7112	1273.7183	-5.55	31	40	0	---	R.LLVVYPWTQR.F
1314.6691	1313.6618	1313.6575	3.26	18	30	0	---	K.VNVDEVGGEALGR.L
1669.8772	1668.8699	1668.8835	-8.14	67	82	0	---	K.VLGAFSDGLAHLDNLK.G
1669.8772	1668.8699	1668.8835	-8.14	67	82	0	(77)	K.VLGAFSDGLAHLDNLK.G
1669.8772	1668.8699	1668.8835	-8.14	67	82	0	91	K.VLGAFSDGLAHLDNLK.G
1797.9766	1796.9693	1796.9785	-5.09	66	82	1	---	K.KVLGAFSDGLAHLDNLK.G

No match to: 979.5460, 1034.1182, 1147.5027, 1195.6837, 1195.6837, 1195.6837, 1205.6095, 1205.6095, 1219.6508, 1219.6508, 1219.6508, 1252.6511, 1267.7006, 1337.6794, 1337.6794, 1342.6943, 1360.6827, 1365.6962, 1367.6936, 1378.6844, 1378.6844, 1383.7865, 1384.7319, 1475.7609, 1529.7290, 1557.7811, 1563.7914, 1572.7805, 1572.7805, 1575.7814, 1585.7865, 1641.8524, 1648.8374, 1742.8954, 1759.9335, 1763.8998, 1771.9310, 1787.9608, 1811.9208, 1816.9521, 1853.9644, 1868.9490, 1871.9698, 1877.0233, 1881.0533, 1881.0533, 1929.9923, 1951.9395, 1994.1302, 2009.0477, 2042.1072, 2042.1072, 2058.9866, 2074.9434, 2107.2268, 2138.1482, 2184.1758, 2202.1494, 2225.1511, 2228.1665, 2235.2302, 2235.2302, 2268.2280, 2286.1941, 2291.1104, 2314.1516, 2326.2490, 2330.1890, 2332.1421, 2360.1626, 3223.6489

15. HBB_SEMEN**Mass:** 15999 **Score:** 128 **Expect:** 8.8e-008 **Matches:** 7

Hemoglobin subunit beta OS=Semnopithecus entellus GN=HBB PE=1 SV=1

Observed	Mr(expt)	Mr(calc)	ppm	Start	End	Miss	Ions	Peptide
1126.5620	1125.5547	1125.5567	-1.76	96	104	0	---	K.LHVDPENFR.L
1274.7185	1273.7112	1273.7183	-5.55	31	40	0	---	R.LLVVYPWTQR.F
1314.6691	1313.6618	1313.6575	3.26	18	30	0	---	K.VNVDEVGGEALGR.L
1669.8772	1668.8699	1668.8835	-8.14	67	82	0	---	K.VLGAFSDGLAHLDNLK.G
1669.8772	1668.8699	1668.8835	-8.14	67	82	0	(77)	K.VLGAFSDGLAHLDNLK.G
1669.8772	1668.8699	1668.8835	-8.14	67	82	0	91	K.VLGAFSDGLAHLDNLK.G
1797.9766	1796.9693	1796.9785	-5.09	66	82	1	---	K.KVLGAFSDGLAHLDNLK.G

No match to: 979.5460, 1034.1182, 1147.5027, 1195.6837, 1195.6837, 1195.6837, 1205.6095, 1205.6095, 1219.6508, 1219.6508, 1219.6508, 1252.6511, 1267.7006, 1337.6794, 1337.6794, 1342.6943, 1360.6827, 1365.6962, 1367.6936, 1378.6844, 1378.6844, 1383.7865, 1384.7319, 1475.7609, 1529.7290, 1557.7811, 1563.7914, 1572.7805, 1572.7805, 1575.7814, 1585.7865, 1641.8524, 1648.8374, 1742.8954, 1759.9335, 1763.8998, 1771.9310, 1787.9608, 1811.9208, 1816.9521, 1853.9644, 1868.9490, 1871.9698, 1877.0233, 1881.0533, 1881.0533, 1929.9923, 1951.9395, 1994.1302, 2009.0477, 2042.1072, 2042.1072, 2058.9866, 2074.9434, 2107.2268, 2138.1482, 2184.1758, 2202.1494, 2225.1511, 2228.1665, 2235.2302, 2235.2302, 2268.2280, 2286.1941, 2291.1104, 2314.1516, 2326.2490, 2330.1890, 2332.1421, 2360.1626, 3223.6489

16. HBD_ATEFU**Mass:** 15882 **Score:** 128 **Expect:** 8.8e-008 **Matches:** 7

Hemoglobin subunit delta OS=Ateles fusciceps GN=HBD PE=1 SV=1

Observed	Mr(expt)	Mr(calc)	ppm	Start	End	Miss	Ions	Peptide
1126.5620	1125.5547	1125.5567	-1.76	96	104	0	---	K.LHVDPENFR.L
1274.7185	1273.7112	1273.7183	-5.55	31	40	0	---	R.LLVVYPWTQR.F
1314.6691	1313.6618	1313.6575	3.26	18	30	0	---	K.VNVDEVGGEALGR.L
1669.8772	1668.8699	1668.8835	-8.14	67	82	0	---	K.VLGAFSDGLAHLDNLK.G
1669.8772	1668.8699	1668.8835	-8.14	67	82	0	(77)	K.VLGAFSDGLAHLDNLK.G
1669.8772	1668.8699	1668.8835	-8.14	67	82	0	91	K.VLGAFSDGLAHLDNLK.G
1797.9766	1796.9693	1796.9785	-5.09	66	82	1	---	K.KVLGAFSDGLAHLDNLK.G

No match to: 979.5460, 1034.1182, 1147.5027, 1195.6837, 1195.6837, 1195.6837, 1205.6095, 1205.6095, 1219.6508, 1219.6508, 1219.6508, 1252.6511, 1267.7006, 1337.6794, 1337.6794, 1342.6943, 1360.6827, 1365.6962, 1367.6936, 1378.6844, 1378.6844, 1383.7865, 1384.7319, 1475.7609, 1529.7290, 1557.7811, 1563.7914, 1572.7805, 1572.7805, 1575.7814, 1585.7865, 1641.8524, 1648.8374, 1742.8954, 1759.9335, 1763.8998, 1771.9310, 1787.9608, 1811.9208, 1816.9521, 1853.9644, 1868.9490, 1871.9698, 1877.0233, 1881.0533, 1881.0533, 1929.9923, 1951.9395, 1994.1302, 2009.0477, 2042.1072, 2042.1072, 2058.9866, 2074.9434, 2107.2268, 2138.1482, 2184.1758, 2202.1494, 2225.1511, 2228.1665, 2235.2302, 2235.2302, 2268.2280, 2286.1941, 2291.1104, 2314.1516, 2326.2490, 2330.1890, 2332.1421, 2360.1626, 3223.6489

17. HBB_ALOBE**Mass:** 16161 **Score:** 128 **Expect:** 8.8e-008 **Matches:** 7

Hemoglobin subunit beta OS=Alouatta belzebul GN=HBB PE=2 SV=3

Observed	Mr(expt)	Mr(calc)	ppm	Start	End	Miss	Ions	Peptide
1126.5620	1125.5547	1125.5567	-1.76	97	105	0	---	K.LHVDPENFR.L
1274.7185	1273.7112	1273.7183	-5.55	32	41	0	---	R.LLVVYPWTQR.F
1314.6691	1313.6618	1313.6575	3.26	19	31	0	---	K.VNVDEVGGEALGR.L
1669.8772	1668.8699	1668.8835	-8.14	68	83	0	---	K.VLGAFSDGLAHLDNLK.G
1669.8772	1668.8699	1668.8835	-8.14	68	83	0	(77)	K.VLGAFSDGLAHLDNLK.G
1669.8772	1668.8699	1668.8835	-8.14	68	83	0	91	K.VLGAFSDGLAHLDNLK.G
1797.9766	1796.9693	1796.9785	-5.09	67	83	1	---	K.KVLGAFSDGLAHLDNLK.G

No match to: 979.5460, 1034.1182, 1147.5027, 1195.6837, 1195.6837, 1195.6837, 1205.6095, 1205.6095, 1219.6508, 1219.6508, 1219.6508, 1252.6511, 1267.7006, 1337.6794, 1337.6794, 1342.6943, 1360.6827, 1365.6962, 1367.6936, 1378.6844, 1378.6844, 1383.7865, 1384.7319, 1475.7609, 1529.7290, 1557.7811, 1563.7914, 1572.7805, 1572.7805, 1575.7814, 1585.7865, 1641.8524, 1648.8374, 1742.8954, 1759.9335, 1763.8998, 1771.9310, 1787.9608, 1811.9208, 1816.9521, 1853.9644, 1868.9490, 1871.9698, 1877.0233, 1881.0533, 1881.0533, 1929.9923, 1951.9395, 1994.1302, 2009.0477, 2042.1072, 2042.1072, 2058.9866, 2074.9434, 2107.2268, 2138.1482, 2184.1758, 2202.1494, 2225.1511, 2228.1665, 2235.2302, 2235.2302, 2268.2280, 2286.1941, 2291.1104, 2314.1516, 2326.2490, 2330.1890, 2332.1421, 2360.1626, 3223.6489

18. HBB_ATEPA **Mass:** 16150 **Score:** 128 **Expect:** 8.8e-008 **Matches:** 7

Hemoglobin subunit beta OS=Ateles paniscus GN=HBB PE=1 SV=3

Observed	Mr(expt)	Mr(calc)	ppm	Start	End	Miss	Ions	Peptide

1126.5620	1125.5547	1125.5567	-1.76	97 -	105	0	---	K.LHVDPENFR.L
1274.7185	1273.7112	1273.7183	-5.55	32 -	41	0	---	R.LLVVYPWTQR.F
1314.6691	1313.6618	1313.6575	3.26	19 -	31	0	---	K.VNVDEVGGEALGR.L
1669.8772	1668.8699	1668.8835	-8.14	68 -	83	0	---	K.VLGAFSDGLAHLDNLK.G
1669.8772	1668.8699	1668.8835	-8.14	68 -	83	0	(77)	K.VLGAFSDGLAHLDNLK.G
1669.8772	1668.8699	1668.8835	-8.14	68 -	83	0	91	K.VLGAFSDGLAHLDNLK.G
1797.9766	1796.9693	1796.9785	-5.09	67 -	83	1	---	K.KVLGAFSDGLAHLDNLK.G

No match to: 979.5460, 1034.1182, 1147.5027, 1195.6837, 1195.6837, 1195.6837, 1205.6095, 1205.6095, 1219.6508, 1219.6508, 1219.6508, 1252.6511, 1267.7006, 1337.6794, 1337.6794, 1342.6943, 1360.6827, 1365.6962, 1367.6936, 1378.6844, 1378.6844, 1383.7865, 1384.7319, 1475.7609, 1529.7290, 1557.7811, 1563.7914, 1572.7805, 1572.7805, 1575.7814, 1585.7865, 1641.8524, 1648.8374, 1742.8954, 1759.9335, 1763.8998, 1771.9310, 1787.9608, 1811.9208, 1816.9521, 1853.9644, 1868.9490, 1871.9698, 1877.0233, 1881.0533, 1881.0533, 1929.9923, 1951.9395, 1994.1302, 2009.0477, 2042.1072, 2042.1072, 2058.9866, 2074.9434, 2107.2268, 2138.1482, 2202.1494, 2225.1511, 2228.1665, 2235.2302, 2235.2302, 2268.2280, 2286.1941, 2291.1104, 2314.1516, 2326.2490, 2330.1890, 2332.1421, 2360.1626, 3223.6489

19. HBB_PITPI **Mass:** 16147 **Score:** 128 **Expect:** 8.8e-008 **Matches:** 7

Hemoglobin subunit beta OS=Pithecia pithecia GN=HBB PE=2 SV=3

Observed	Mr(expt)	Mr(calc)	ppm	Start	End	Miss	Ions	Peptide
1126.5620	1125.5547	1125.5567	-1.76	97 -	105	0	---	K.LHVDPENFR.L
1274.7185	1273.7112	1273.7183	-5.55	32 -	41	0	---	R.LLVVYPWTQR.F
1314.6691	1313.6618	1313.6575	3.26	19 -	31	0	---	K.VNVDEVGGEALGR.L
1669.8772	1668.8699	1668.8835	-8.14	68 -	83	0	---	K.VLGAFSDGLAHLDNLK.G
1669.8772	1668.8699	1668.8835	-8.14	68 -	83	0	(77)	K.VLGAFSDGLAHLDNLK.G
1669.8772	1668.8699	1668.8835	-8.14	68 -	83	0	91	K.VLGAFSDGLAHLDNLK.G
1797.9766	1796.9693	1796.9785	-5.09	67 -	83	1	---	K.KVLGAFSDGLAHLDNLK.G

No match to: 979.5460, 1034.1182, 1147.5027, 1195.6837, 1195.6837, 1195.6837, 1205.6095, 1205.6095, 1219.6508, 1219.6508, 1219.6508, 1252.6511, 1267.7006, 1337.6794, 1337.6794, 1342.6943, 1360.6827, 1365.6962, 1367.6936, 1378.6844, 1378.6844, 1383.7865, 1384.7319, 1475.7609, 1529.7290, 1557.7811, 1563.7914, 1572.7805, 1572.7805, 1575.7814, 1585.7865, 1641.8524, 1648.8374, 1742.8954, 1759.9335, 1763.8998, 1771.9310, 1787.9608, 1811.9208, 1816.9521, 1853.9644, 1868.9490, 1871.9698, 1877.0233, 1881.0533, 1881.0533, 1929.9923, 1951.9395, 1994.1302, 2009.0477, 2042.1072, 2042.1072, 2058.9866, 2074.9434, 2107.2268, 2138.1482, 2184.1758, 2202.1494, 2225.1511, 2228.1665, 2235.2302, 2235.2302, 2268.2280, 2286.1941, 2291.1104, 2314.1516, 2326.2490, 2330.1890, 2332.1421, 2360.1626, 3223.6489

20. HBB_SAISC **Mass:** 16147 **Score:** 128 **Expect:** 8.8e-008 **Matches:** 7

Hemoglobin subunit beta OS=Saimiri sciureus GN=HBB PE=1 SV=2

Observed	Mr(expt)	Mr(calc)	ppm	Start	End	Miss	Ions	Peptide
1126.5620	1125.5547	1125.5567	-1.76	97 -	105	0	---	K.LHVDPENFR.L
1274.7185	1273.7112	1273.7183	-5.55	32 -	41	0	---	R.LLVVYPWTQR.F
1314.6691	1313.6618	1313.6575	3.26	19 -	31	0	---	K.VNVEDVGGEALGR.L
1669.8772	1668.8699	1668.8835	-8.14	68 -	83	0	---	K.VLGAFSDGLAHLDNLK.G
1669.8772	1668.8699	1668.8835	-8.14	68 -	83	0	(77)	K.VLGAFSDGLAHLDNLK.G
1669.8772	1668.8699	1668.8835	-8.14	68 -	83	0	91	K.VLGAFSDGLAHLDNLK.G
1797.9766	1796.9693	1796.9785	-5.09	67 -	83	1	---	K.KVLGAFSDGLAHLDNLK.G

No match to: 979.5460, 1034.1182, 1147.5027, 1195.6837, 1195.6837, 1195.6837, 1205.6095, 1205.6095, 1219.6508, 1219.6508, 1219.6508, 1252.6511, 1267.7006, 1337.6794, 1337.6794, 1342.6943, 1360.6827, 1365.6962, 1367.6936, 1378.6844, 1378.6844, 1383.7865, 1384.7319, 1475.7609, 1529.7290, 1557.7811, 1563.7914, 1572.7805, 1572.7805, 1575.7814, 1585.7865, 1641.8524, 1648.8374, 1742.8954, 1759.9335, 1763.8998, 1771.9310, 1787.9608, 1811.9208, 1816.9521, 1853.9644, 1868.9490, 1871.9698, 1877.0233, 1881.0533, 1881.0533, 1929.9923, 1951.9395, 1994.1302, 2009.0477, 2042.1072, 2042.1072, 2058.9866, 2074.9434, 2107.2268, 2138.1482, 2184.1758, 2202.1494, 2225.1511, 2228.1665, 2235.2302, 2235.2302, 2268.2280, 2286.1941, 2291.1104, 2314.1516, 2326.2490, 2330.1890, 2332.1421, 2360.1626, 3223.6489

Search Parameters

Type of search :Sequence Query
Enzyme :Trypsin
Fixed modifications :Carbamidomethyl (C)
Variable modifications : Oxidation (M)
Mass values :Monoisotopic
Protein Mass :Unrestricted
Peptide Mass Tolerance : ± 80 ppm
Fragment Mass Tolerance: ± 0.3 Da
Max Missed Cleavages :1
Instrument type :MALDI-TOF-TOF
Query1 (979.5460,1+) :<no title>
Query2 (1034.1182,1+) :<no title>
Query3 (1126.5620,1+) :<no title>
Query4 (1147.5027,1+) :<no title>
Query5 (1195.6837,1+) :<no title>
Query6 (1195.6837,1+) :MaldiWellID: 66572, SpectrumID: 144466,
Query7 (1195.6837,1+) :MaldiWellID: 66572, SpectrumID: 144458,
Query8 (1205.6095,1+) :<no title>
Query9 (1205.6095,1+) :MaldiWellID: 66572, SpectrumID: 144459,
Query10 (1219.6508,1+) :<no title>
Query11 (1219.6508,1+) :MaldiWellID: 66572, SpectrumID: 144467,
Query12 (1219.6508,1+) :MaldiWellID: 66572, SpectrumID: 144460,
Query13 (1252.6511,1+) :<no title>
Query14 (1267.7006,1+) :<no title>
Query15 (1274.7185,1+) :<no title>
Query16 (1314.6691,1+) :<no title>
Query17 (1337.6794,1+) :<no title>
Query18 (1337.6794,1+) :MaldiWellID: 66572, SpectrumID: 144465,
Query19 (1342.6943,1+) :<no title>
Query20 (1360.6827,1+) :<no title>
Query21 (1365.6962,1+) :<no title>
Query22 (1367.6936,1+) :<no title>

```
Query23 (1378.6844,1+) : <no title>
Query24 (1378.6844,1+) : MaldiWellID: 66572, SpectrumID: 144457,
Query25 (1383.7865,1+) : <no title>
Query26 (1384.7319,1+) : <no title>
Query27 (1475.7609,1+) : <no title>
Query28 (1529.7290,1+) : <no title>
Query29 (1557.7811,1+) : <no title>
Query30 (1563.7914,1+) : <no title>
Query31 (1572.7805,1+) : <no title>
Query32 (1572.7805,1+) : MaldiWellID: 66572, SpectrumID: 144464,
Query33 (1575.7814,1+) : <no title>
Query34 (1585.7865,1+) : <no title>
Query35 (1641.8524,1+) : <no title>
Query36 (1648.8374,1+) : <no title>
Query37 (1669.8772,1+) : <no title>
Query38 (1669.8772,1+) : MaldiWellID: 66572, SpectrumID: 144468,
Query39 (1669.8772,1+) : MaldiWellID: 66572, SpectrumID: 144461,
Query40 (1742.8954,1+) : <no title>
Query41 (1759.9335,1+) : <no title>
Query42 (1763.8998,1+) : <no title>
Query43 (1771.9310,1+) : <no title>
Query44 (1787.9608,1+) : <no title>
Query45 (1797.9766,1+) : <no title>
Query46 (1811.9208,1+) : <no title>
Query47 (1816.9521,1+) : <no title>
Query48 (1853.9644,1+) : <no title>
Query49 (1868.9490,1+) : <no title>
Query50 (1871.9698,1+) : <no title>
Query51 (1877.0233,1+) : <no title>
Query52 (1881.0533,1+) : <no title>
Query53 (1881.0533,1+) : MaldiWellID: 66572, SpectrumID: 144456,
Query54 (1929.9923,1+) : <no title>
Query55 (1951.9395,1+) : <no title>
Query56 (1994.1302,1+) : <no title>
Query57 (2009.0477,1+) : <no title>
Query58 (2042.1072,1+) : <no title>
Query59 (2042.1072,1+) : MaldiWellID: 66572, SpectrumID: 144463,
Query60 (2058.9866,1+) : <no title>
Query61 (2074.9434,1+) : <no title>
Query62 (2107.2268,1+) : <no title>
Query63 (2138.1482,1+) : <no title>
Query64 (2184.1758,1+) : <no title>
Query65 (2202.1494,1+) : <no title>
Query66 (2225.1511,1+) : <no title>
Query67 (2228.1665,1+) : <no title>
Query68 (2235.2302,1+) : <no title>
Query69 (2235.2302,1+) : MaldiWellID: 66572, SpectrumID: 144462,
Query70 (2268.2280,1+) : <no title>
Query71 (2286.1941,1+) : <no title>
Query72 (2291.1104,1+) : <no title>
Query73 (2314.1516,1+) : <no title>
Query74 (2326.2490,1+) : <no title>
Query75 (2330.1890,1+) : <no title>
Query76 (2332.1421,1+) : <no title>
Query77 (2360.1626,1+) : <no title>
Query78 (3223.6489,1+) : <no title>
```

Mascot Search Results

Protein View

Band: 2

Match to: **HBB_HUMAN** Score: **201** Expect: **4.4e-015**
Hemoglobin subunit beta OS=Homo sapiens GN=HBB PE=1 SV=2

Nominal mass (Mr): **16102**; Calculated pI value: **6.75**

NCBI BLAST search of HBB_HUMAN against nr
Unformatted sequence string for pasting into other applications

Taxonomy: Homo sapiens

Fixed modifications: Carbamidomethyl (C)
Variable modifications: Oxidation (M)
Cleavage by Trypsin: cuts C-term side of KR unless next residue is P
Sequence Coverage: **61%**

Matched peptides shown in **Bold Red**

```
  1 MVHLTPEEKS AVTALWGKVN VDEVGGEALG RLLVVYPWTQ RFFESFGDLS
 51 TPDAVMGNPK VKAHGKKVLG AFSDGLAHLD NLKGTFATLS ELHCDKLHVD
101 PENFRLLGNV LVCVLAHHFG KEFTPPVQAA YQKVVAGVAN ALAHKYH
```

Show predicted peptides also

Sort Peptides By Residue Number Increasing Mass Decreasing Mass

Start - End	Observed	Mr(expt)	Mr(calc)	ppm	Miss	Sequence	
10 - 31	2228.1665	2227.1592	2227.1597	-0	1	K.SAVTALWGKVNVDEVGGEALGR.L	(No match)
19 - 31	1314.6691	1313.6618	1313.6575	3	0	K.VNVDEVGGEALGR.L	(No match)
32 - 41	1274.7185	1273.7112	1273.7183	-6	0	R.LLVVYPWTQR.F	(No match)
42 - 60	2058.9866	2057.9793	2057.9405	19	0	R.FFESFGDLSTPDAVMGNPK.V	(No match)
42 - 60	2074.9434	2073.9361	2073.9354	0	0	R.FFESFGDLSTPDAVMGNPK.V Oxidation (M)	(No match)
42 - 62	2286.1941	2285.1868	2285.1038	36	1	R.FFESFGDLSTPDAVMGNPKVK.A	(No match)
67 - 83	1797.9766	1796.9693	1796.9785	-5	1	K.KVLGAFSDGLAHLDNLK.G	(No match)
68 - 83	1669.8772	1668.8699	1668.8835	-8	0	K.VLGAFSDGLAHLDNLK.G	(No match)
68 - 83	1669.8772	1668.8699	1668.8835	-8	0	K.VLGAFSDGLAHLDNLK.G	(Ions score 77)
68 - 83	1669.8772	1668.8699	1668.8835	-8	0	K.VLGAFSDGLAHLDNLK.G	(Ions score 91)
97 - 105	1126.5620	1125.5547	1125.5567	-2	0	K.LHVDPENFR.L	(No match)
122 - 133	1378.6844	1377.6771	1377.6929	-11	0	K.EFTPPVQAAYQK.V	(No match)
122 - 133	1378.6844	1377.6771	1377.6929	-11	0	K.EFTPPVQAAYQK.V	(Ions score 20)

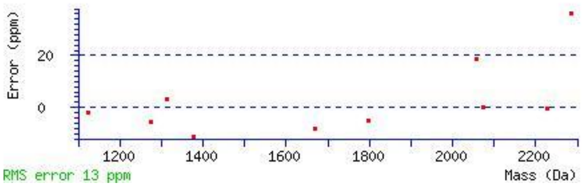

Mascot Search Results

**Band**	: **2**
Search title	: SampleSetID: 759, AnalysisID: 6291, MaldiWellID: 66572, SpectrumID: 143906, Path=\171004\MSMS\17-112 NCBI alveolata
Database	: NCBInr 20120508 (17919084 sequences; 6150218869 residues)
Taxonomy	: Plasmodium falciparum (malaria parasite) (19026 sequences)
Timestamp	: 29 Nov 2017 at 15:05:52 GMT
Warning	: A Peptide summary report will usually give a much clearer picture of MS/MS search results.
Top Score	: 64 for gi|160537, surface protein [Plasmodium falciparum]

Mascot Score Histogram

Protein score is -10*Log(P), where P is the probability that the observed match is a random event.
Protein scores greater than 55 are significant (p<0.05).
Protein scores are derived from ions scores as a non-probabilistic basis for ranking protein hits.

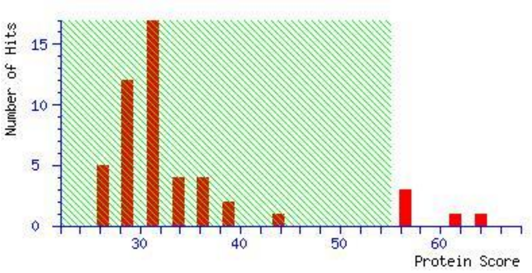

Protein Summary Report

Format As | Protein Summary (deprecated) | Help

Significance threshold p< 0.05 Max. number of hits 20

Re-Search All | Search Unmatched

Index

	Accession	Mass	Score	Description
1.	gi|160537	27813	64	surface protein [Plasmodium falciparum]
2.	gi|160112	33774	62	beta-galactosidase, partial [Plasmodium falciparum]
3.	gi|124512406	7438257		heat shock 70 kDa protein [Plasmodium falciparum 3D7]
4.	gi|226045	74741	57	heat shock protein
5.	gi|123598	74754	57	RecName: Full=Heat shock 70 kDa protein; Short=HSP70; AltName: Full=74.3 kDa protein; AltName: Full=Cytoplasmic antigen
6.	gi|78032833	14212	44	erythrocyte membrane protein [Plasmodium falciparum]
7.	gi|124801272	3693039		conserved Plasmodium protein [Plasmodium falciparum 3D7]
8.	gi|124806675	69389239		conserved Plasmodium protein [Plasmodium falciparum 3D7]
9.	gi|90193411	**23053**	**37**	**erythrocyte membrane protein 1 [Plasmodium falciparum]**
10.	gi|296005038	3057336		14-3-3 protein, putative [Plasmodium falciparum 3D7]
11.	gi|78040295	**15497**	**36**	**erythrocyte membrane protein [Plasmodium falciparum]**
12.	gi|258597288	18514635		conserved Plasmodium protein [Plasmodium falciparum 3D7]
13.	gi|124514018	4744434		aminomethyltransferase, mitochondrial precursor [Plasmodium falciparum 3D7]
14.	gi|86171727	**38013**	**33**	**conserved Plasmodium protein, unknown function [Plasmodium falciparum 3D7]**
15.	gi|124512370	12187133		2-oxoglutarate dehydrogenase E1 component [Plasmodium falciparum 3D7]
16.	gi|124804435	6056533		TCP-1/cpn60 chaperonin family [Plasmodium falciparum 3D7]
17.	gi|124802981	8244332		dynamin-like protein [Plasmodium falciparum 3D7]
18.	gi|124806323	6130831		t-complex protein 1, gamma subunit, putative [Plasmodium falciparum 3D7]
19.	gi|48639487	82415	31	dynamin homologue [Plasmodium falciparum]
20.	gi|90193419	22992	31	erythrocyte membrane protein 1 [Plasmodium falciparum]

Results List

1. gi|160537 **Mass:** 27813 **Score:** 64 **Expect:** 0.0076 **Matches:** 4

surface protein [Plasmodium falciparum]

Observed	Mr(expt)	Mr(calc)	ppm	Start	End	Miss	Ions	Peptide
1219.6508	1218.6435	1218.6509	-6.09	50 -	60	0	---	K.FHLDGIPPAPR.K
1219.6508	1218.6435	1218.6509	-6.09	50 -	60	0	51	K.FHLDGIPPAPR.K
1337.6794	1336.6721	1336.6003	53.8	189 -	200	0	---	K.EAESVCAPIMSK.I + Oxidation (M)
1365.6962	1364.6889	1364.6572	23.3	175 -	186	1	---	K.NQLAGKDEYEAK.Q

No match to: 979.5460, 1034.1182, 1126.5620, 1147.5027, 1195.6837, 1195.6837, 1205.6095, 1205.6095, 1252.6511, 1267.7006, 1274.7185, 1314.6691, 1342.6943, 1360.6827, 1367.6936, 1378.6844, 1378.6844, 1383.7865, 1384.7319, 1475.7609, 1529.7290, 1557.7811, 1563.7914, 1572.7805, 1575.7814, 1585.7865, 1641.8524, 1648.8374, 1669.8772, 1742.8954, 1759.9335, 1763.8998, 1771.9310, 1787.9608, 1797.9766, 1811.9208, 1816.9521, 1853.9644, 1868.9490, 1871.9698, 1877.0233, 1881.0533, 1881.0533, 1929.9923, 1951.9395, 1994.1302, 2009.0477, 2042.1072, 2058.9866, 2074.9434, 2107.2268, 2138.1482, 2184.1758, 2202.1494, 2225.1511, 2228.1665, 2235.2302, 2268.2280, 2286.1941, 2291.1104, 2314.1516, 2326.2490, 2330.1890, 2332.1421, 2360.1626, 3223.6489

2. gi|160112 **Mass:** 33774 **Score:** 62 **Expect:** 0.012 **Matches:** 4

beta-galactosidase, partial [Plasmodium falciparum]

Observed	Mr(expt)	Mr(calc)	ppm	Start	End	Miss	Ions	Peptide
1219.6508	1218.6435	1218.6509	-6.09	106 -	116	0	---	K.FHLDGIPPAPR.K
1219.6508	1218.6435	1218.6509	-6.09	106 -	116	0	51	K.FHLDGIPPAPR.K
1337.6794	1336.6721	1336.6003	53.8	245 -	256	0	---	K.EAESVCAPIMSK.I + Oxidation (M)

Observed	Mr(expt)	Mr(calc)	ppm	Start	End	Miss	Ions	Peptide
1365.6962	1364.6889	1364.6572	23.3	231	- 242	1	---	K.NQLAGKDEYEAK.Q

No match to: 979.5460, 1034.1182, 1126.5620, 1147.5027, 1195.6837, 1195.6837, 1205.6095, 1205.6095, 1252.6511, 1267.7006, 1274.7185, 1314.6691, 1342.6943, 1360.6827, 1367.6936, 1378.6844, 1378.6844, 1383.7865, 1384.7319, 1475.7609, 1529.7290, 1557.7811, 1563.7914, 1572.7805, 1575.7814, 1585.7865, 1641.8524, 1648.8374, 1669.8772, 1742.8954, 1759.9335, 1763.8998, 1771.9310, 1787.9608, 1797.9766, 1811.9208, 1816.9521, 1853.9644, 1868.9490, 1871.9698, 1877.0233, 1881.0533, 1881.0533, 1929.9923, 1951.9395, 1994.1302, 2009.0477, 2042.1072, 2058.9866, 2074.9434, 2107.2268, 2138.1482, 2184.1758, 2202.1494, 2225.1511, 2228.1665, 2235.2302, 2268.2280, 2286.1941, 2291.1104, 2314.1516, 2326.2490, 2330.1890, 2332.1421, 2360.1626, 3223.6489

3. gi|124512406 **Mass:** 74382 **Score:** 57 **Expect:** 0.041 **Matches:** 4

heat shock 70 kDa protein [Plasmodium falciparum 3D7]

Observed	Mr(expt)	Mr(calc)	ppm	Start	End	Miss	Ions	Peptide
1219.6508	1218.6435	1218.6509	-6.09	472	- 482	0	---	K.FHLDGIPPAPR.K
1219.6508	1218.6435	1218.6509	-6.09	472	- 482	0	51	K.FHLDGIPPAPR.K
1337.6794	1336.6721	1336.6003	53.8	611	- 622	0	---	K.EAESVCAPIMSK.I + Oxidation (M)
1365.6962	1364.6889	1364.6572	23.3	597	- 608	1	---	K.NQLAGKDEYEAK.Q

No match to: 979.5460, 1034.1182, 1126.5620, 1147.5027, 1195.6837, 1195.6837, 1205.6095, 1205.6095, 1252.6511, 1267.7006, 1274.7185, 1314.6691, 1342.6943, 1360.6827, 1367.6936, 1378.6844, 1378.6844, 1383.7865, 1384.7319, 1475.7609, 1529.7290, 1557.7811, 1563.7914, 1572.7805, 1575.7814, 1585.7865, 1641.8524, 1648.8374, 1669.8772, 1742.8954, 1759.9335, 1763.8998, 1771.9310, 1787.9608, 1797.9766, 1811.9208, 1816.9521, 1853.9644, 1868.9490, 1871.9698, 1877.0233, 1881.0533, 1881.0533, 1929.9923, 1951.9395, 1994.1302, 2009.0477, 2042.1072, 2058.9866, 2074.9434, 2107.2268, 2138.1482, 2184.1758, 2202.1494, 2225.1511, 2228.1665, 2235.2302, 2268.2280, 2286.1941, 2291.1104, 2314.1516, 2326.2490, 2330.1890, 2332.1421, 2360.1626, 3223.6489

4. gi|226045 **Mass:** 74741 **Score:** 57 **Expect:** 0.041 **Matches:** 4

heat shock protein

Observed	Mr(expt)	Mr(calc)	ppm	Start	End	Miss	Ions	Peptide
1219.6508	1218.6435	1218.6509	-6.09	472	- 482	0	---	K.FHLDGIPPAPR.K
1219.6508	1218.6435	1218.6509	-6.09	472	- 482	0	51	K.FHLDGIPPAPR.K
1337.6794	1336.6721	1336.6003	53.8	611	- 622	0	---	K.EAESVCAPIMSK.I + Oxidation (M)
1365.6962	1364.6889	1364.6572	23.3	597	- 608	1	---	K.NQLAGKDEYEAK.Q

No match to: 979.5460, 1034.1182, 1126.5620, 1147.5027, 1195.6837, 1195.6837, 1205.6095, 1205.6095, 1252.6511, 1267.7006, 1274.7185, 1314.6691, 1342.6943, 1360.6827, 1367.6936, 1378.6844, 1378.6844, 1383.7865, 1384.7319, 1475.7609, 1529.7290, 1557.7811, 1563.7914, 1572.7805, 1575.7814, 1585.7865, 1641.8524, 1648.8374, 1669.8772, 1742.8954, 1759.9335, 1763.8998, 1771.9310, 1787.9608, 1797.9766, 1811.9208, 1816.9521, 1853.9644, 1868.9490, 1871.9698, 1877.0233, 1881.0533, 1881.0533, 1929.9923, 1951.9395, 1994.1302, 2009.0477, 2042.1072, 2058.9866, 2074.9434, 2107.2268, 2138.1482, 2184.1758, 2202.1494, 2225.1511, 2228.1665, 2235.2302, 2268.2280, 2286.1941, 2291.1104, 2314.1516, 2326.2490, 2330.1890, 2332.1421, 2360.1626, 3223.6489

5. gi|123598 **Mass:** 74754 **Score:** 57 **Expect:** 0.041 **Matches:** 4

RecName: Full=Heat shock 70 kDa protein; Short=HSP70; AltName: Full=74.3 kDa protein; AltName: Full=Cytoplasmic antigen

Observed	Mr(expt)	Mr(calc)	ppm	Start	End	Miss	Ions	Peptide
1219.6508	1218.6435	1218.6509	-6.09	472	- 482	0	---	K.FHLDGIPPAPR.K
1219.6508	1218.6435	1218.6509	-6.09	472	- 482	0	51	K.FHLDGIPPAPR.K
1337.6794	1336.6721	1336.6003	53.8	611 -	622	0	---	K.EAESVCAPIMSK.I + Oxidation (M)
1365.6962	1364.6889	1364.6572	23.3	597 -	608	1	---	K.NQLAGKDEYEAK.Q

No match to: 979.5460, 1034.1182, 1126.5620, 1147.5027, 1195.6837, 1195.6837, 1205.6095, 1205.6095, 1252.6511, 1267.7006, 1274.7185, 1314.6691, 1342.6943, 1360.6827, 1367.6936, 1378.6844, 1378.6844, 1383.7865, 1384.7319, 1475.7609, 1529.7290, 1557.7811, 1563.7914, 1572.7805, 1575.7814, 1585.7865, 1641.8524, 1648.8374, 1669.8772, 1742.8954, 1759.9335, 1763.8998, 1771.9310, 1787.9608, 1797.9766, 1811.9208, 1816.9521, 1853.9644, 1868.9490, 1871.9698, 1877.0233, 1881.0533, 1881.0533, 1929.9923, 1951.9395, 1994.1302, 2009.0477, 2042.1072, 2058.9866, 2074.9434, 2107.2268, 2138.1482, 2184.1758, 2202.1494, 2225.1511, 2228.1665, 2235.2302, 2268.2280, 2286.1941, 2291.1104, 2314.1516, 2326.2490, 2330.1890, 2332.1421, 2360.1626, 3223.6489

Search Parameters

```
Type of search         :Sequence Query
Enzyme                 :Trypsin
Fixed modifications    :Carbamidomethyl (C)
Variable modifications :Oxidation (M)
Mass values            :Monoisotopic
Protein Mass           :Unrestricted
Peptide Mass Tolerance : ± 80 ppm
Fragment Mass Tolerance: ± 0.3 Da
Max Missed Cleavages   :1
Instrument type        :MALDI-TOF-TOF
Query1 (979.5460,1+)   :<no title>
Query2 (1034.1182,1+)  :<no title>
Query3 (1126.5620,1+)  :<no title>
Query4 (1147.5027,1+)  :<no title>
Query5 (1195.6837,1+)  :<no title>
Query6 (1195.6837,1+)  :MaldiWellID: 66572, SpectrumID: 143909,
Query7 (1205.6095,1+)  :<no title>
Query8 (1205.6095,1+)  :MaldiWellID: 66572, SpectrumID: 143910,
Query9 (1219.6508,1+)  :<no title>
 Query10 (1219.6508,1+) :MaldiWellID: 66572, SpectrumID: 143911,
 Query11 (1252.6511,1+) :<no title>
 Query12 (1267.7006,1+) :<no title>
 Query13 (1274.7185,1+) :<no title>
 Query14 (1314.6691,1+) :<no title>
 Query15 (1337.6794,1+) :<no title>
 Query16 (1342.6943,1+) :<no title>
 Query17 (1360.6827,1+) :<no title>
 Query18 (1365.6962,1+) :<no title>
 Query19 (1367.6936,1+) :<no title>
 Query20 (1378.6844,1+) :<no title>
 Query21 (1378.6844,1+) :MaldiWellID: 66572, SpectrumID: 143908,
 Query22 (1383.7865,1+) :<no title>
 Query23 (1384.7319,1+) :<no title>
 Query24 (1475.7609,1+) :<no title>
 Query25 (1529.7290,1+) :<no title>
 Query26 (1557.7811,1+) :<no title>
 Query27 (1563.7914,1+) :<no title>
 Query28 (1572.7805,1+) :<no title>
 Query29 (1575.7814,1+) :<no title>
 Query30 (1585.7865,1+) :<no title>
 Query31 (1641.8524,1+) :<no title>
 Query32 (1648.8374,1+) :<no title>
 Query33 (1669.8772,1+) :<no title>
 Query34 (1742.8954,1+) :<no title>
 Query35 (1759.9335,1+) :<no title>
 Query36 (1763.8998,1+) :<no title>
 Query37 (1771.9310,1+) :<no title>
 Query38 (1787.9608,1+) :<no title>
 Query39 (1797.9766,1+) :<no title>
 Query40 (1811.9208,1+) :<no title>
 Query41 (1816.9521,1+) :<no title>
 Query42 (1853.9644,1+) :<no title>
 Query43 (1868.9490,1+) :<no title>
 Query44 (1871.9698,1+) :<no title>
 Query45 (1877.0233,1+) :<no title>
 Query46 (1881.0533,1+) :<no title>
 Query47 (1881.0533,1+) :MaldiWellID: 66572, SpectrumID: 143907,
 Query48 (1929.9923,1+) :<no title>
 Query49 (1951.9395,1+) :<no title>
 Query50 (1994.1302,1+) :<no title>
 Query51 (2009.0477,1+) :<no title>
 Query52 (2042.1072,1+) :<no title>
 Query53 (2058.9866,1+) :<no title>
 Query54 (2074.9434,1+) :<no title>
 Query55 (2107.2268,1+) :<no title>
 Query56 (2138.1482,1+) :<no title>
 Query57 (2184.1758,1+) :<no title>
 Query58 (2202.1494,1+) :<no title>
 Query59 (2225.1511,1+) :<no title>
 Query60 (2228.1665,1+) :<no title>
 Query61 (2235.2302,1+) :<no title>
 Query62 (2268.2280,1+) :<no title>
 Query63 (2286.1941,1+) :<no title>
 Query64 (2291.1104,1+) :<no title>
 Query65 (2314.1516,1+) :<no title>
 Query66 (2326.2490,1+) :<no title>
 Query67 (2330.1890,1+) :<no title>
 Query68 (2332.1421,1+) :<no title>
 Query69 (2360.1626,1+) :<no title>
 Query70 (3223.6489,1+) :<no title>
```

Mascot Search Results

Protein View

Band: 2

Match to: **gi|160537** Score: **64** Expect: **0.0076**
surface protein [Plasmodium falciparum]

Nominal mass (Mr): **27813**; Calculated pI value: **5.06**

NCBI BLAST search of gi|160537 against nr
Unformatted sequence string for pasting into other applications

Taxonomy: Plasmodium falciparum

Fixed modifications: Carbamidomethyl (C)
Variable modifications: Oxidation (M)
Cleavage by Trypsin: cuts C-term side of KR unless next residue is P
Sequence Coverage: **13%**

Matched peptides shown in **Bold Red**

```
  1 MTKLIERNTT IPAKKSQIFT TYADNQPGVL IQVYEGERAL TKDNNLLGKF
 51 HLDGIPPAPR KVPQIEVTFD IDANGILNVT AVEKSTGKQN HITITNDKGR
101 LSQDEIDRMV NDAEKYKAED EENRKRIEAR NSLENYCYGV KSSLEDQKIK
151 EKLQPAEIET CMKTITTILE WLEKNQLAGK DEYEAKQKEA ESVCAPIMSK
201 IYQDAAGAAG GMPGGMPGGM PGGMPGGMNF PGGMPGAGMP GNAPAGSGPT
251 VEEVD
```

Show predicted peptides also

Sort Peptides By ◉ Residue Number ○ Increasing Mass ○ Decreasing Mass

Start - End	Observed	Mr(expt)	Mr(calc)	ppm	Miss	Sequence	
50 - 60	1219.6508	1218.6435	1218.6509	-6	0	K.FHLDGIPPAPR.K	(No match)
50 - 60	1219.6508	1218.6435	1218.6509	-6	0	K.FHLDGIPPAPR.K	(Ions score 51)
175 - 186	1365.6962	1364.6889	1364.6572	23	1	K.NQLAGKDEYEAK.Q	(No match)
189 - 200	1337.6794	1336.6721	1336.6003	54	0	K.EAESVCAPIMSK.I	Oxidation (M) (No match)

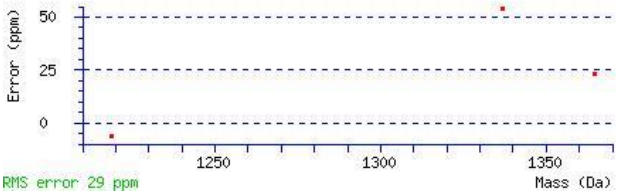

Mascot Search Results

Protein View

Band: 2

Match to: **gi|160537** Score: **64** Expect: **0.0076**
surface protein [Plasmodium falciparum]

Nominal mass (M_r): **27813**; Calculated pI value: **5.06**

NCBI BLAST search of gi|160537 against nr
Unformatted sequence string for pasting into other applications

Taxonomy: Plasmodium falciparum

Fixed modifications: Carbamidomethyl (C)
Variable modifications: Oxidation (M)
Cleavage by Trypsin: cuts C-term side of KR unless next residue is P
Sequence Coverage: **13%**

Matched peptides shown in **Bold Red**

```
  1 MTKLIERNTT IPAKKSQIFT TYADNQPGVL IQVYEGERAL TKDNNLLGKF
 51 HLDGIPPAPR KVPQIEVTFD IDANGILNVT AVEKSTGKQN HITITNDKGR
101 LSQDEIDRMV NDAEKYKAED EENRKRIEAR NSLENYCYGV KSSLEDQKIK
151 EKLQPAEIET CMKTITTILE WLEKNQLAGK DEYEAKQKEA ESVCAPIMSK
201 IYQDAAGAAG GMPGGMPGGM PGGMPGGMNF PGGMPGAGMP GNAPAGSGPT
251 VEEVD
```

Show predicted peptides also

Sort Peptides By ◉ Residue Number ○ Increasing Mass ○ Decreasing Mass

Start - End	Observed	Mr(expt)	Mr(calc)	ppm	Miss	Sequence	
50 - 60	1219.6508	1218.6435	1218.6509	-6	0	K.FHLDGIPPAPR.K	(No match)
50 - 60	1219.6508	1218.6435	1218.6509	-6	0	K.FHLDGIPPAPR.K	(Ions score 51)
175 - 186	1365.6962	1364.6889	1364.6572	23	1	K.NQLAGKDEYEAK.Q	(No match)
189 - 200	1337.6794	1336.6721	1336.6003	54	0	K.EAESVCAPIMSK.I	Oxidation (M) (No match)

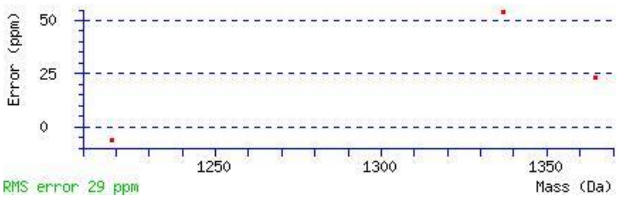

Mascot: http://www.matrixscience.com/
