# Supplementary material for: Sickle Cell Trait Induces Oxidative Damage on Plasmodium falciparum Proteome at Erythrocyte Stages
Source: Int J Mol Sci. 2019 Nov 16;20(22):5769. doi: 10.3390/ijms20225769 (PMC6888313; doi:10.3390/ijms20225769)
Supplement: Supplementary file 1 [file ijms-20-05769-s001.zip › ijms-590016 suppl for final/Supp_Mat_1 for final.pdf]

Supplementary Material 1

Sickle Cell Trait Induce Oxidative Damage on *Plasmodium falciparum* Proteome at Erythrocyte Stages

Supplementary Tables

Supplementary table 1. Summary of harvested cultures of *P. falciparum* 3D7 at high parasitaemia.

Donor	Condition	Vol. (μL)	% Parasitemia/stage		
			Rings	Trophozoites	Schizonts
NC	HbAA	750	38%	35%	37%
			38%	38%	37%
			40%	---	40%
ST	HbAS	800	34%	38%	35%
			38%	34%	36%
			37%	35%	32%
MC	HbAS	800	37%	35%	38%
			36%	37%	38%
			---	37%	38%
MJ	HbAS	800	40%	38%	38%
			40%	36%	38%
			40%	37%	38%

Triplicates of synchronous cultures at high parasitaemia were harvested for control (HbAA) and sickle cell trait carriers (HbAS) groups.

Supplementary table 2. Parasite proteins yielding obtained from synchronous cultures of *P. falciparum* 3D7 at high parasitaemias.

Donor	Condition	Yielding by Asexual Stage (μg parasite proteins/μL of iRBC)		
		Rings	Trophozoites	Schizonts
NC	HbAA	1.1 ± 0.4	0.6 ± 0.02	0.6 ± 0.3
ST	HbAS	1.2 ± 0.4	1.3 ± 0.6	2.3 ± 0.7
MC	HbAS	1.4 ± 0.2	2.2 ± 0.2	1.8 ± 0.2
MJ	HbAS	0.9 ± 0.08	1.1 ± 0.07	1.2 ± 0.2

Supplementary table 3. Data used to evaluate repeatability and reproducibility of carbonyl calibration curve.

C.I.	Repeatability - Day 1 (2 Membranes)			Day 2 (1 Membrane)	Reproducibility	
	Spot intensities*			Spot intensities*		
	Curve 1	Curve 2	Mean ± SD	Curve 3	Mean ± SD	RSD
0.76	1.24	1.36	1.30 ± 0.09	1.28	1.29 ± 0.06	0.05
1.49	1.75	1.55	1.65 ± 0.14	1.61	1.64 ± 0.10	0.06
2.03	1.66	1.75	1.71 ± 0.06	1.76	1.72 ± 0.06	0.03
2.58	1.97	2.12	2.05 ± 0.11	1.89	2.00 ± 0.12	0.06
3.49	2.12	2.13	2.12 ± 0.00	2.08	2.11 ± 0.03	0.01
4.40	2.36	2.26	2.31 ± 0.07	2.25	2.29 ± .06	0.03
6.21	2.82	2.67	2.75 ± 0.11	2.73	2.74 ± 0.08	0.03
8.03	3.25	3.26	3.25 ± 0.01	3.17	3.22 ± 0.05	0.01
13.79	4.46	4.76	4.60 ± 0.21	4.41	4.54 ± 0.19	0.04
17.68	5.85	5.55	5.70 ± 0.21	5.80	5.73 ± 0.16	0.03

C.I.: Carbonyl index in nmoles of CO/mg of protein. Three calibration curves of BSA by Dot Blot were used for the determination of carbonyls index. *The values of the intensities are expressed in pixels/μm and were adjusted to simple numbers dividing by a factor of 10^6.

Supplementary table 4. Parameters to evaluation of repeatability and reproducibility of carbonyl index calibration curves.

Parameter	Repeatability /Reproducibility Curves				
	Curve 1 (day 1)	Curve 2 (day 1)	Curve 3 (day 2)	Mean ± SD	RSD (%)
**Slope (m)**	0.2528	0.2468	0.2508	0.2501 ± 0.0031	1.22
**Intercept (b)**	1.2182	1.2493	1.181	1.2162 ± 0.0342	2.81
**Correlation coefficient (r^2)**	0.9914	0.9942	0.9939	0.9932 ± 0.0015	0.15

Expressed as means ± SD, seemed in 3 membrane independent.

Supplementary table 5. Intensities obtained for carbonylated protein bands of *P. falciparum* in Western blot assay.

Donnor	Condition	Rings	Trophozoites	Schizonts
NC	HbAA	9.09 ± 2.2	18.92 ± 3.66	5.42 ± 0.35
ST	HbAS	29.29 ± 12.76	16.42 ± 3.00	21.75 ± 3.49
MJ	HbAS	32.28 ± 18.33	34.19 ± 5.80	23.23 ± 7.03
MC	HbAS	41.4 ± 5.38	21.16 ± 7.64	32.73 ± 4.32

Analysis of intensities of *P. falciparum* protein of rings, trophozoites and schizonts obtained on control and HbAS carrier.

Supplementary table 6. Relationship of carbonylation of *P. falciparum* protein in patterns band detected on HbAS carrier with respect control

**Quotien Intensities HbAS Carrier/Control HbAA**			
HbAS/HbAA	Rings	Trophozoites	Schizonts
ST/NC	32	0.9	4.0
MJ/NC	3.6	1.8	4.3
MC/NC	4.6	1.1	6.0
Average ± SD	3.8 ± 0.69	1.3 ± 0.49	4.8 ± 1.10

Supplementary Figures

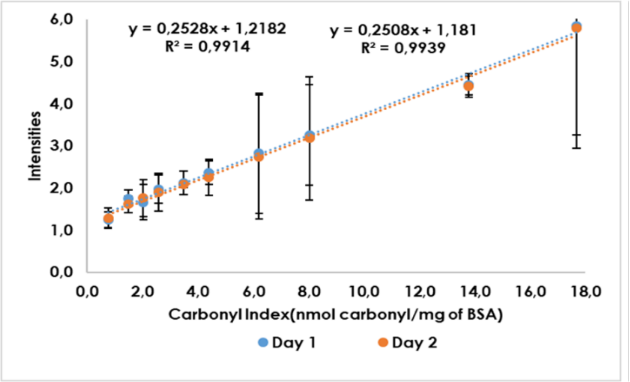

Figure S1. Repeatability of calibration curve employed to quantify carbonyl index of parasite protein. **A.** Repeatability of calibration curve. **B**. Reproducibility of calibration curve. Data were obtained from three independent calibration curves assayed in two days different. Intensities adjusted were calculated as Mean ± SD. BSA: Bovine Serum Albumin.

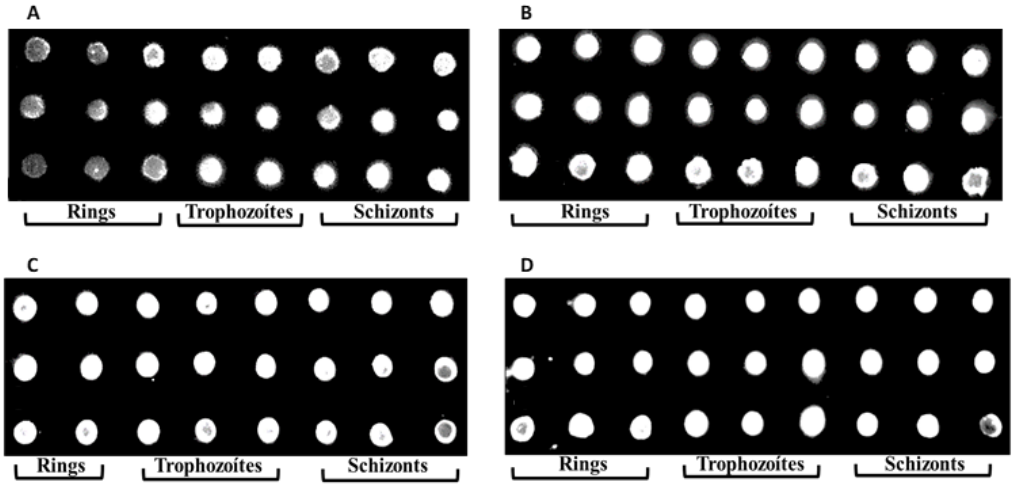

Figure S2. Dot blot membranes of *P. falciparum* carbonylated proteins across erythrocyte stages. Panels show samples of HbAA control (**A**) and HbAS carriers (**B**, **C** and **D**). 200 ng of protein of *P. falciparum* were spotted by triplicate on PVDF membranes.

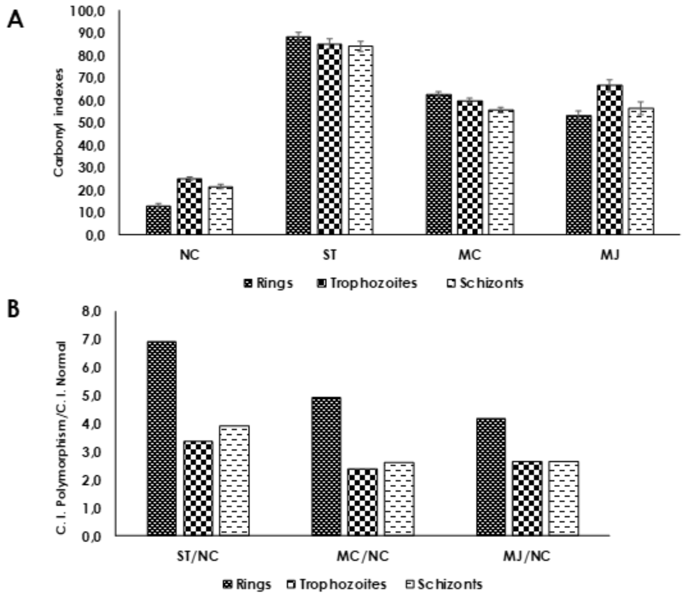

Figure S3. Behavior of carbonyl indexes on *P. falciparum* proteome at different moments.
A. Graph shows carbonyl index values (means ± SD) for proteins obtained from asexual stages of parasites cultured in HbAA (NC) and HbAS (ST, MC and MJ) erythrocytes. **B**. Relative increasing of carbonyl indexes values calculated for samples with the quotient: carbonyl index of mutated/ carbonyl index of normal control.
